# scTrans: Sparse attention powers fast and accurate cell type annotation in single-cell RNA-seq data

**DOI:** 10.1371/journal.pcbi.1012904

**Published:** 2025-04-04

**Authors:** Zhiyi Zou, Ying Liu, Yuting Bai, Jiawei Luo, Zhaolei Zhang

**Affiliations:** 1 College of Computer Science and Electronic Engineering, Hunan University, Changsha, Hunan, China; 2 Donnelly Centre for Cellular and Biomolecular Research, University of Toronto, Toronto, Ontario, Canada; 3 Department of Computer Science, University of Toronto, Toronto, Ontario, Canada; 4 Department of Molecular Genetics, University of Toronto, Toronto, Ontario, Canada; George Washington University, UNITED STATES OF AMERICA

## Abstract

Cell type annotation is crucial in single-cell RNA sequencing data analysis because it enables significant biological discoveries and deepens our understanding of tissue biology. Given the high-dimensional and highly sparse nature of single-cell RNA sequencing data, most existing annotation tools focus on highly variable genes to reduce dimensionality and computational load. However, this approach inevitably results in information loss, potentially weakening the model’s generalization performance and adaptability to novel datasets. To mitigate this issue, we developed scTrans, a **s**ingle **c**ell **Trans**former-based model, which employs sparse attention to utilize all non-zero genes, thereby effectively reducing the input data dimensionality while minimizing information loss. We validated the speed and accuracy of scTrans by performing cell type annotation on 31 different tissues within the Mouse Cell Atlas. Remarkably, even with datasets nearing a million cells, scTrans efficiently perform cell type annotation in limited computational resources. Furthermore, scTrans demonstrates strong generalization capabilities, accurately annotating cells in novel datasets and generating high-quality latent representations, which are essential for precise clustering and trajectory analysis.

## Author summary

Accurately annotating cell types from single-cell RNA-seq data is essential for exploring cellular diversity, functional differences, and gaining insights into biological processes and disease mechanisms. Advances in single-cell technologies have generated large scale data with high dimensionality and sparsity, which is difficult to feature extraction. Some existing methods apply highly variable genes selection to reduce dimension, which may overlook crucial information contained in low-variability genes and affect the accuracy of annotation. This paper introduces scTrans, a deep learning framework based on the Transformer architecture that integrates sparse attention mechanisms to efficiently process single-cell RNA-seq data. This approach focuses on non-zero gene features for cell type identification, minimizes information loss, significantly reduces computational complexity and hardware resource consumption, enhances both processing efficiency and classification accuracy. We validated the proposed model for cell type annotation tasks across a broad dataset, assessing its accuracy and efficiency. Additionally, we tested the model’s robustness and generalization ability by comparing the quality of latent representation generated on novel datasets.

## Introduction

In single-cell RNA sequencing (scRNA-seq) technology, researchers can conduct gene expression profiling at single-cell level. This allows identification and study of cellular differences [1], facilitating the study of cellular and tissue heterogeneity [2] and providing a foundation to our understanding of development and diseases [3]. Following the generation of scRNA-seq data, an essential downstream analysis step is cell type annotation [4].

Conventional cell type annotation methods require clustering cells and identifying marker genes for each cluster through differential expression analysis, which is often time-consuming [5]. As the scale of single-cell data increases, conventional annotation methods become infeasible due to the complexity and abundance of the data. Several automated cell type annotation methods have been proposed [6–8], however, the high noise level and sparsity in scRNA-seq data can affect the effectiveness of these methods.

Recently, several deep learning-based methods were developed, which can extract informative and compact features from noisy, sparse, and high-dimensional scRNA-seq datasets [9]. Pre-trained large scale model have achieved considerable success in tasks ranging from natural language processing and structure ligand interactions, which has inspired similar research on single-cell data analysis [10,11,15]. Often called foundational models, these pre-trained models start with massive collection of scRNA-seq data, followed with pre-training by using Transformer architecture for self-supervised learning to obtain embeddings, and then fine-tuned with task specific datasets for downstream analysis. scBert [10] is a large-scale pre-trained language model using BERT architecture, fine-tuned for cell type annotation. scGPT [11] is a generative foundational model, which can be fine-tuned for various downstream tasks, such as cell type annotation, multi-batch integration and gene network inference. scFoundation [12] is a large pre-trained model with 100 million parameters, trained on over 50 million human single-cell transcriptomic profiles, capable of effectively capturing complex gene relationships among cells and achieving state-of-the-art performance in various single-cell analysis tasks. CellPLM [13] is a novel single-cell pre-training model that effectively encodes cell-cell relationships by treating cells as tokens in a language model, tissues as sentences, and integrating spatial transcriptomic data along with a Gaussian mixture prior distribution, achieving fast and accurate predictions across various downstream tasks. Geneformer [14] is a pre-trained deep learning model that leverages transfer learning on large-scale single-cell transcriptomic data for pre-training and fine-tunes on specific biological tasks to enhance predictive accuracy in data-limited scenarios. GenePT [15] is a special foundation model, as it does not require expensive pre-training process. Instead, it uses ChatGPT to generate gene embeddings from text descriptions of genes, and then performs a series of downstream tasks based on these embeddings. Similar to this method, another approach combines the standard processing pipeline for single cells with the GPT-4 model [16], using prompts to guide GPT-4 in annotating cell type clusters based on differentially expressed genes. Although this method achieves certain effectiveness, it was not intended for deeper exploration of gene expression program in cells. Concerto [17] uses contrastive learning in the pre-training stage to construct a self-distillation contrastive model, this model can be applied to multiple downstream tasks. The aforementioned methods share a common feature, as they use all the genes as input for feature extraction in order to fully explore information in cells. Despite the reported success of these methods, most of them have the limitation of extreme computing complexity, long training time and demand on GPU and RAMs.

To address the challenges arising from high-dimensionalities while using only limited available computing resources, several methods adopt principal component analysis (PCA) to obtain low dimensional embedding for downstream analysis [18,19]. Despite the reduced complexity, PCA has the limitation of information loss, and sensitivity to noise that is intrinsic in single-cell data. And PCA is prone to batch effects such that the principal components found in multiple datasets may differ substantially due to sample heterogeneity. In contrast, several methods have adopted a two-step strategy by first selecting a set of highly variable genes (HVG), then learning low dimensional embedding representation on these HVGs. A common model involves leveraging autoencoder architectures, such as AutoClass [20], which use encoding and decoding processes to generate a latent representation conducive to cell type annotation. While classic autoencoder are often insufficient in addressing batch effects, modifications have been made. For example, iMap [21], combines autoencoders with generative adversarial networks (GAN) to achieve a unified latent representation distribution across diverse batches. HDMC [22] integrates domain adaption and contrastive learning within the autoencoder framework to enhance representation learning and correct batch effects. Given the limited availability of labeled data for cell type annotation tasks, several semi-supervised methods have emerged. Among these is itClust [23], a transfer learning method that harnesses pre-training on labeled data to derive low-dimensional representations, subsequently applying these representations to optimize cluster and cell type annotation on the target dataset. Similarly, scSemiGAN [24] utilizes GAN to derive latent representation, while training classification using labeled data.

Despite the advantages of using HVG to reduce dimensionality, this approach also faces some challenges. Firstly, excessive removal of genes may potentially compromise the performance of downstream analysis. Genes that are important in cell type differentiation may not necessarily fall within the HVG. Secondly, as described above, batch effect persist as an issue in HVG based approaches, as the sets of observed HVGs may differ across datasets. To overcome these issues, we develop a new method, scTrans, as described below.

In simple terms, scTrans maps genes to a high-dimensional vector space, then leveraging sparse attention based on a Transformer architecture to aggregate genes of non-zero value for representation learning, mitigate the problems of information loss and batch effects associated with HVG, and reduce computational and hardware burden. The scTrans method comprises two main stages: pre-training and fine-tuning. During the pre-training phase, scTrans fully exploits unlabeled data through unsupervised contrastive learning. In the subsequent fine-tuning phase, it utilizes labeled data for supervised learning, resulting in a robust tool for cell type annotation and feature extraction.

Compared with other methods, scTrans has the following advantages:

(1) It reduces the dimensionality of input features while minimizing information loss, allowing for sufficient representation of large datasets with limited hardware resource.
(2) It demonstrates strong robustness and generalization capabilities, achieves accurate cross batch annotation on novel datasets, and generates high-quality representations.
(3) It provides interpretability; the attention weights are informative and can identify genes that are functionally critical or have the potential to serve as biomarkers.

We benchmarked scTrans against several newly developed deep learning based methods on mouse cell atlas (MCA) [25], three peripheral blood mononuclear cell dataset (PBMC45k [26], PBMC160k [27] and scBloodNL [28]), mouse brain and mouse pancreas datasets, and T cell and dendritic cell development datasets [29,30]. The MCA dataset consists of 31 tissues, scTrans achieved strong annotation performance, even with very few labeled cells. On the PBMC160k and scBloodNL datasets, which had more cells, scTrans achieved the highest evaluation results while requiring the shortest runtime. On datasets with batch effects, including PBMC45k, mouse brain, and mouse pancreas, scTrans achieved accurate annotation results in identifying cell types, and generated high-quality latent representation for clustering analysis. On the cell development datasets of human T cells and mouse dendritic cells, scTrans accurately inferred the developmental trajectory of cells.

## Results

### Overview of scTrans

**Fig 1:**
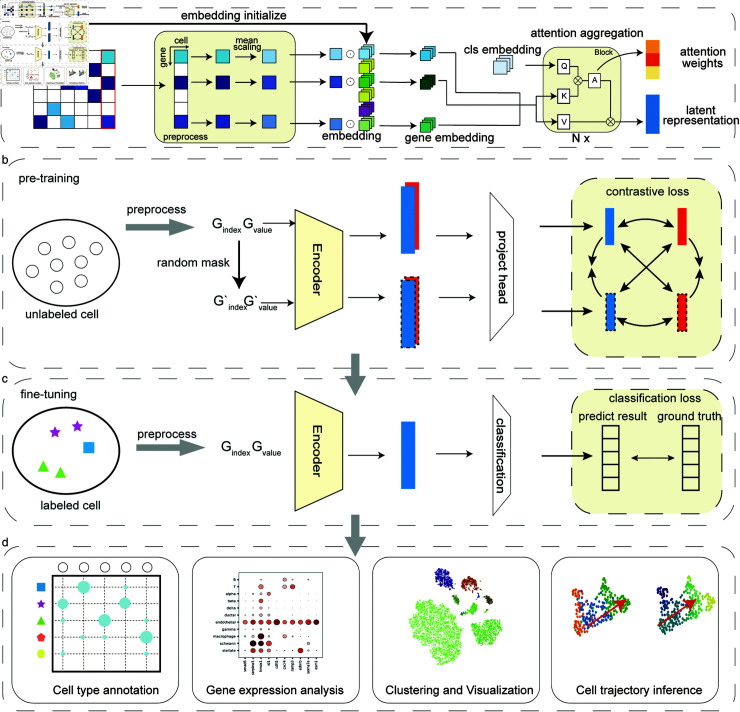
Overview of scTrans. (a) Sparse attention aggregates cellular representation: scTrans leverages a sparse attention mechanism to efficiently encode non-zero genes into cellular representations. We assign each gene an embedding based on its gene symbol and only use the embeddings corresponding to genes with non-zero expression values, aggregating these embeddings through attention weights, enabling focused learning on informative genes. (b) Contrastive pre-training strategy: During pre-training, scTrans generates augmented cells through random masking, creating positive pairs with the original cells and negative pairs with other cells in the batch. Features for contrastive learning are extracted via an encoder-projection architecture. This process pulls similar positive pairs closer and pushes negative pairs apart in the latent space, facilitating unsupervised pre-training. (c) Fine-tuning for cell type classification: In the fine-tuning phase, a classification layer is appended after the latent representation layer, enabling supervised learning for cell type classification using labeled data. Model parameters are optimized accordingly. This optimization is achieved through supervised learning using labeled data. (d) Applications in downstream tasks: Trained scTrans can be deployed for cell type annotation on novel datasets, as well as for downstream tasks such as gene expression analysis, clustering or cell trajectory inference.

scTrans utilizes sparse attention mechanism to aggregate features from genes for cell representation learning (Fig 1A). Specifically, we first extract the non-zero expressed genes from each cell, then map these genes to their corresponding gene embeddings, and use gene expression values for dot product encoding. Gene embeddings are initialized by applying PCA on the gene-cell expression matrix, and are updated during the training process. Finally, we use a trainable cls embedding to aggregate information from gene embedding through attention mechanisms to obtain cellular representations. Our attention aggregation module consists of multiple layers of blocks, the specific structure is shown in the method and S1 Fig.

The training process is divided into two main parts: pre-training, and fine-tuning (Fig 1B and 1C). In the pre-training (Fig 1B), we introduce contrastive learning based on SIMCLR [31] to enhance gene and cls embedding, since PCA-initialized embeddings are prone to the noise in the scRNA-seq data, which may not be ideal for cell classification tasks. In the fine-tuning stage (Fig 1C), a linear layer serves as the classification layer after the encoder, and we then use labeled data for supervised learning. After fine-tuning, scTrans can be used for cell type annotation in novel datasets, generating high-quality latent representation for downstream tasks such as clustering or cell trajectory analysis. And through the attention weights, we can identify critical genes for further analysis (Fig 1D).

### scTrans achieves accurate cell type annotation at datasets of different scales

Cell type annotation is an important and often the first step in scRNA-seq data analysis. In this section, we assessed the cell type annotation performance of scTrans on datasets of different scales. We tested on datasets from MCA [25]; the number of cells per sample vary from 1102 to 28658 (details in S1 Table), and also tested on two larger datasets PBMC160k and scBloodNL, which had 161,764 and 928,275 cells respectively. We compared scTrans with a pre-train fine-tuning method Concerto [17], two semi-supervised methods itclust [23] and scSemiGAN [24], and two supervised methods scDeepSort [32] and TOSICA [33].

The MCA datasets consists of 31 individual dataset corresponding to 31 mouse tissues. For each tissue, we applied stratified sampling and selected 10*τ* = 3 labeled cells for training, while set the remaining 90*α* = 1 cells for predicting. The model performance was evaluated based on accuracy and f1-macro. We ran each methods five times and presented averaged metrics in

**Fig 2:**
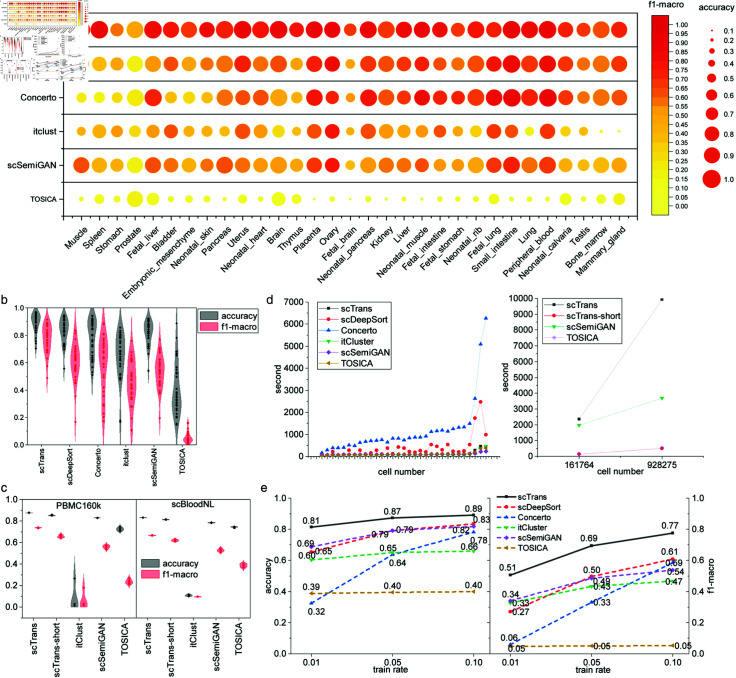
Comparison annotation performance across multiple datasets and scales. (a) The average accuracy and f1-macro of each tissue in MCA datasets. (b) The accuracy and f1-macro violin plot, including 31 tissues of MCA datasets, with each point representing the average annotation result of a tissue. (c) The accuracy and f1-macro violin plot of PBMC160k and scBloodNL datasets, with 10 percent of stratified sampling label cell, five times repeated experiments at different randomized seed. (d) The runtime performance of scTrans and comparative methods at MCA, PBMC160k and scBloodNL datasets. The figure on the left shows the running time of the 31 tissues in MCA, and the number of cells increases with the x-coordinate. The figure on the right shows the running time of four methods at PBMC160k and scBloodNL datasets. (e) The average accuracy and f1-macro under 1*r* = 1, 5*δ*, and 10*n* = 4 labeled cells in 31 tissues of MCA.

Fig 2A. The standard deviations for all the tissues are shown in Fig 2B, and S2 and S3 Tables. Fig 2A and S2 Table show that scTrans achieves the best accuracy on each datasets, except for the Fetal Lung tissue, which still achieved 86*β* = 1 accuracy and ranked third. Fig 2B shows that scTrans exhibited the highest average accuracy and f1-macro scores across 31 tissues.

On average for 31 tissues, scTrans achieved 6*γ* = 0 . 2 higher accuracy and 16*α* = 0 higher f1-macro than the second best method scDeepSort. We noted TOSICA performed poorly on all tissues, possibly due to its requirement for a sufficient amount of labeled data to train effectively, 10*β* = 1 of labeled data may not be sufficient.

For Muscle and Spleen tissues, which had fewer than 2000 cells, scTrans still achieved annotation accuracy of nearly 95*Î³* = 0 . 2, which was about 5*Î±* = 1pace>1 and 9*β* = 1 higher than the second-best method scSemiGAN, and 39*τ* = 3 and 43*α* = 0 higher than scSemiGAN on f1-macro. Although Concerto was also a pre-train fine-tuning method, it did not achieve as goods performance as scTrans on Muscle and Spleen datasets, probably it required larger training set to construct an effective model. As the number of cells increased, Concerto’s performance improved (Fig 2A). scDeepSort utilized all genes of labeled cells, achieving high accuracy even in small cell numbers, and had the second or third best performance in most tissues.

For several tissues comprising tens of thousands of cells (Testis, Bone marrow and Mammary gland), scTrans, Concerto, and scDeepSort produced comparable levels of accuracy. However, scTrans had higher f1-macro than other methods (S3 Table). Itclust got poor performance on these three datasets; this may be due to the instability of itclust when there is a large number of labeled data.

We conducted the same annotation benchmark experiments on two datasets with larger cell numbers, PBMC160k [27] (161,764 cells) and scBloodNL [28] (928,275 cells). We were unable to run Concerto and scDeepSort on these two datasets as they take all the genes as inputs and have large RAM requirement (larger than 40Gb). Thus we only compared scTrans with three other methods that only use HVG and had smaller RAM requirement, i.e., TOSICA, scSemiGAN, and itClust. We repeated the experiment five times on PBMC160k and scBloodNL datasets, each time using a different randomized seed. To expedite scTrans, we introduced a simplified version scTrans-short, which only had one pre-training epoch and ten fine-tuning epochs. scTrans-short was intended to demonstrate that scTrans can achieve better results than other methods with less runtime. Notably, scTrans-short achieved the second-best performance (Fig 2C), closely behind scTrans, while exhibiting the shortest running time among all the evaluated methods (Fig 2D).

A primary advantage and major motivation of scTrans was its short running time. We compared the running time of scTrans with other methods on each tissue of MCA (Fig 2D). scTrans had a shorter runtime than Concerto and scDeepSort, both of which utilized all the genes in MCA. Concerto was the most time-consuming in all tissues, likely due to its transformer architecture and the use of all genes. On larger datasets, PBMC160k and scBloodNL, we compared with scSemiGAN and TOSICA. As mentioned earlier, scTrans-short had the second-best evaluation results and achieved similar runtime as TOSICA, which utilized HVGs as inputs.

We next investigated how annotation performance was influenced by the number of labeled training data, which is typically scarce in these tasks. We selected 1*β* = 1 and 5*τ* = 3 of the training set as labeled data and repeated the annotation experiments on MCA, PBMC160K and scBloodNL datasets. Fig 2E shows that scTrans consistently outperformed other methods (also see S2 Fig and S4 Table). We noted that scTrans had more accurate annotation with 5*X* = *Bx* of the labeled data than all other methods with 10*U* ≈ − *ln* ⁡  *P* labeled data, even achieving higher accuracy than Concerto with only 1*t* < 0 labeled data. Concerto was greatly affected by the

number of labels, which is probably due to the possibility of overfitting in Concerto when there are too few labels.

We conducted another experiment to explore the impact of different levels of cell type annotations on the results, utilizing the three hierarchical levels provided by the PBMC160k dataset. The details of the experiment are in S1 Text. As shown in S5 Table, the annotation accuracy reached 98% at the broadest level of cell type annotation (Level 1). As the level of cell type annotation gradually became more detailed, the accuracy decreases, but even at the most detailed level of cell type annotation (Level 3), the model still achieved a high accuracy about 88%.

We incorporated two large-scale pre-training methods, CellPLM and scGPT, into our comparison. Since these two models were pre-trained on human single-cell transcriptomics datasets, we conducted comparative experiments with these two methods using the PBMC160k dataset. During the experiments, we employed both zero-shot and fine-tuning strategies for cell type annotation. As shown in S6 Table, in the zero-shot scenario, scTrans outperformed the two large models, possibly due to the fact that cell types in PBMC160k datasets are highly subdivided, down to the cellular subtype level, resulting into suboptimal results for the zero-shot approach. We then fine-tuned the two large models, and their performance significantly improved compared to the zero-shot results, yet they still fell short of scTrans. This may be because large models had pre-trained on massive datasets, which making the models robust and generalizable, may also weaken their sensitivity to identify cell subtypes. The large model had been trained on extensive data, produced informative gene embeddings. We tested using these embeddings to initialize our method on the PBMC160k dataset (results in S7 Table). However, this did not yield positive results, possibly due to the embeddings’ robustness making it harder for scTrans to capture the cell subtype differences in PBMC160k.

scTrans relied on non-zero expression genes, which heavily depends on sequencing depth in single cell sequencing experiments. To explore how sequencing depth affected the performance of scTrans, we simulated datasets with different sequencing depths using splatter [34], and evaluated the annotation performance of scTrans on these datasets. For details in simulation experiments, please refer to the S1 Text. The results were presented in the S3 Fig. It was found that with the larger sequencing depth, the annotation accuracy of the scTrans model showed a gradual increase trend. It was worth noting that when the sequencing depth was relatively low, the scTrans was more affected by the number of cell types. And when lib.loc increased to 7.5 (with a non-zero gene expression rate of about 7%), the performance of scTrans is almost not affected. However, even when the lib.loc value is 7 (with only 5% of non-zero gene expression), scTrans still achieves good results.

Based on these annotation experiments, it was observed that scTrans could accurately and efficiently annotate cells while fully utilizing a large number of genes.

### scTrans is robust to batch effects and achieves accurate cross batch annotation

Due to variations in experimental conditions, technical platform, and sample heterogeneity, scRNA-seq datasets of the same biological sample often had substantial batch effects. Batch effect could result in incorrect calibration of gene expression and inaccuracy in cell type annotation [35,36]. Most methods to reduce the impact of batch effects required both reference and query datasets as input at the same time, in order to generate a batch corrected embedding for cell type annotation. However, each time cell type annotation was executed, these methods required training the model from scratch using reference and query datasets, which was a laborious and time-consuming process. scTrans fully utilized all non-zero genes in unlabeled and labeled data, making it robust and generalized. Therefore, scTrans achieved accurate cell type annotation on novel datasets despite there was no batch information.

**Fig 3:**
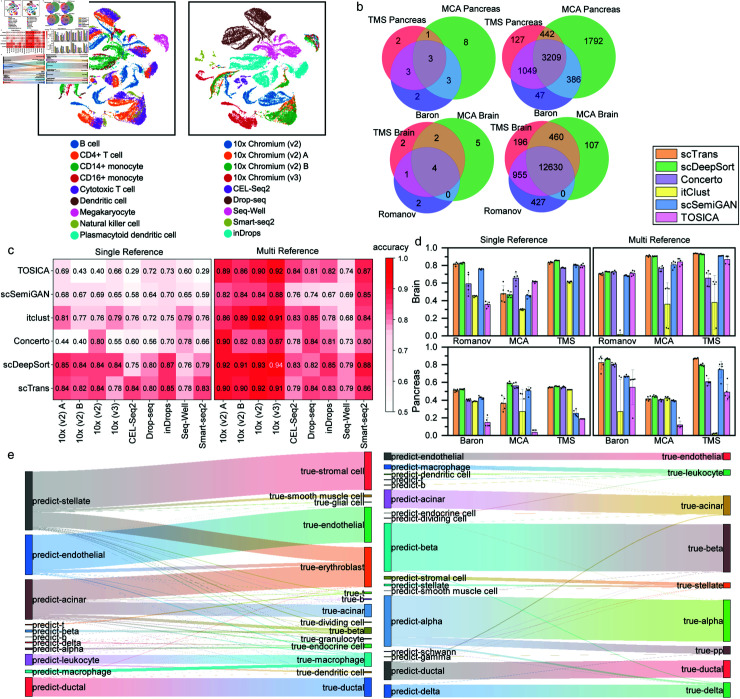
Cross batch annotation on PBMC45k, mouse brain and mouse pancreas datasets. (a) UMAP visualization of PCA embedding of PBMC45k dataset, including its cell types and technologies, showing batch effects within the dataset. (b) Venn diagrams of the mouse pancreas and mouse brain datasets, illustrating the overlaps between different datasets, indicating differences in cellular composition. The left side represents the overlap in the number of identical cells between different datasets, while the right side shows the overlap in the number of identical cell types. (c) Average accuracy heatmap of annotation task result in PBMC45K, including the results of different methods using different technical datasets on *single reference* annotation and *multi reference* annotation tasks. (d) Comparison of accuracy of annotation results for different methods in *single reference* and *multi reference* annotation task on mouse pancreas and mouse brain datasets. Error bars were based on mean and 95*%* confidence. (e) The sankey diagram showing annotation results of scTrans in *multi reference* annotation task, on the left is MCA Pancreas and on the right is TMS Pancreas.

To evaluate the performance of scTrans in cross batch annotation, we complied the following datasets for benchmark experiments: (1) PBMC45k [26], sequencing data of peripheral blood cells from two donors using seven different sequencing technologies from a single laboratory (S8 Table). Fig 3A shows significant differences among different technologies and among donors(also see S4 Fig). (2) Mouse brain, we selected mouse brain datasets from MCA [25], the Tabula Muris (TMS) dataset [37] and Romanov et al. [38]. These datasets were generated in different laboratories by different technologies from different samples, which had stronger batch effects than PBMC45k and more differences in cell type composition (Fig 3B, S9 Table). (3) Mouse Pancreas, we selected mouse pancreas datasets from MCA [25], the Tabula Muris (TMS) dataset [37] and Baron et al. [39]. These three datasets, like the mouse brain datasets, were generated from three different laboratories that utilized different samples and technologies.

As shown in Fig 3B, the mouse pancreas datasets had higher level of challenge to annotation tools, as manifested in having more sample-specific cell types than mouse brain (S9 Table). We designed two benchmark experiments: *single reference* annotation task and *multi-reference* annotation task. The *single reference* annotation task involved using one batch dataset to annotate other batch datasets. In contrast, in the *multi reference* annotation task, we evaluated the model’s ability to effectively integrate multiple reference datasets to annotate a single batch dataset. We conducted repeated experiments five times on both tasks and benchmarked scTrans against Concerto, itclust, scSemiGAN, scDeepSort and TOSICA.

**PBMC45k datasets.** We regarded different types of technology in the PBMC45k dataset as different batches and compared the performance of scTrans with other methods on both *single reference and *multi reference* annotation tasks.* As shown in Fig 3C, we compared the performance between scTrans and other methods on the PBMC45k dataset (also see S4 Fig and S10 Table). In the *single reference annotation* task, we used the dataset of one technology for model training and tested it on the datasets of other technologies. scTrans has the highest average performance, achieving the first or second place in accuracy or f1-macro among most technologies. scTrans and scDeepSort achieved similar results for most technologies, and for two datasets with the fewest cells (generated by Smart-seq2 and CEL-seq2), scTrans performed better, with an accuracy and f1-macro improvements about 4*r* = 1, 9*%* and 11*%*, 5*%* (Fig 3C and S10 Table).

In the multi-reference annotation task, we selected the dataset of one technology for testing and used the datasets of the remaining technologies for training. As shown in Fig 3C and S4 Fig, scTrans achieved the second-best average accuracy and f1-macro. TOSICA achieved the higher average accuracy in *multi reference* annotation tasks, however, it did not perform well in *single reference*. One possible reason was that TOSICA relied heavily on the availability of labeled data, while there were fewer available labels in *single reference* benchmark experiments. We also compared our model with the two large models, scGPT and CellPLM, the comparison results are shown in S6 Table. Our performance is slightly inferior to the two large models, which may be because the large models have been trained on vast datasets and thus possess stronger generalization capabilities. We also tried large model gene embeddings instead of PCA for initialization. As shown in S7 Table, large model gene embeddings had a positive effect in most datasets, likely due to the generalization ability of gene embeddings learned by the large model.

**Mouse Brain datasets.** We next conducted the *single reference* and *multi reference* annotation tasks again on mouse brain datasets. In the *single reference* annotation task, we used one dataset from one laboratory for training and tested on datasets from other laboratories. The annotation results are shown in Fig 3D and S11 Table, scTrans had the second-best performance in average accuracy on TMS Brain and Romanov datasets, and achieved best or second-best average f1-macro on all the three datasets (S5 Fig). In the *multi reference* annotation task, we tested on one dataset from one laboratory while using datasets from the remaining laboratories for training. As demonstrated in S11 Table, scTrans achieved the highest average accuracy and f1-macro on the TMS Brain and MCA Brain datasets. As shown in the sankey diagram (Fig 3E and S6 Fig), some cell clusters were almost annotated as another cell type. Some annotation errors were resulted because scTrans recognized more precise cell subtypes. Astroglial cells in MCA Brain were most annotated as Bergmann glial cell by scTrans (S6 Fig), we next visualized the expression of Bergmann glial marker gene Tnc [40] [41], and observed a high degree of overlap between the expression of marker gene *Tnc* with the distribution of astroglial in latent representation (S7 Fig). It is worth noting that there were only 40 Bergmann glial cells during the training process, accounting for 0.4*%* of the total number of cells during the training process (S9 Table). And some annotation errors were due to the similarity between cell types. Vascular smooth muscle (VSM) cells were annotated as brain pericyte cells in Romanov dataset, and on the TMS Brain dataset, scTrans annotated most of the brain pericyte cells as VSM (S6 Fig). It can be explained by the fact that both brain pericyte and VSM belong to mural cell.

**Mouse Pancreas datasets.** We conducted the same experiments on the mouse pancreas datasets as we did on the mouse brain dataset. As shown in Fig 3D, scTrans and scDeepSort still had better performance than other methods on most datasets (also see S5 Fig). For *single reference* task, scTrans achieved second-best average accuracy in TMS Pancreas and Baron datasets, and got best or second-best average f1-macro on these two datasets (S11 Fig). For *multi reference* task, scTrans was significantly better than the second-best method scDeepSort on TMS Pancreas, with a 7*%* improvement in accuracy. On Baron datasets, scTrans achieved second-best average accuracy and f1-macro (S11 Table). We also analyzed the annotation results in *multi reference* task. Stromal cells and smooth muscle cells were annotated as stellate cells by scTrans in MCA Pancreas datasets (Fig 3E). And scTrans also annotated some stellate cells as stromal cells in TMS Pancreas. These can be explained by the fact that stellate cells are also a type of stromal cell, which is an important cell in the pancreatic cancer stroma and plays a crucial role in pancreatic health and disease [42]. And activated stellate cell express *α*-smooth muscle actin, which is a marker typically associated with smooth muscle cells [42,43]. Moreover, some macrophage and most dendritic cells were annotated by scTrans as leukocyte cells in TMS Pancreas (Fig 3E). And in the Baron datasets, scTrans annotated some T cells and B cells as leukocyte (S6 Fig). It is noted that T cells, B cells, macrophages, and dendritic cells are all subtypes of leukocyte.

### scTrans identifies informative genes via attention mechanism

We identified genes that distinguish cell types or are potentially important in cell type specific biological processes in mouse brain and mouse pancreas datasets via attention mechanism. For each datasets, we calculated the attention weights of all genes and selected them with higher attention weights in each cluster for analysis (More details in methods), which we called critical genes.

**Fig 4:**
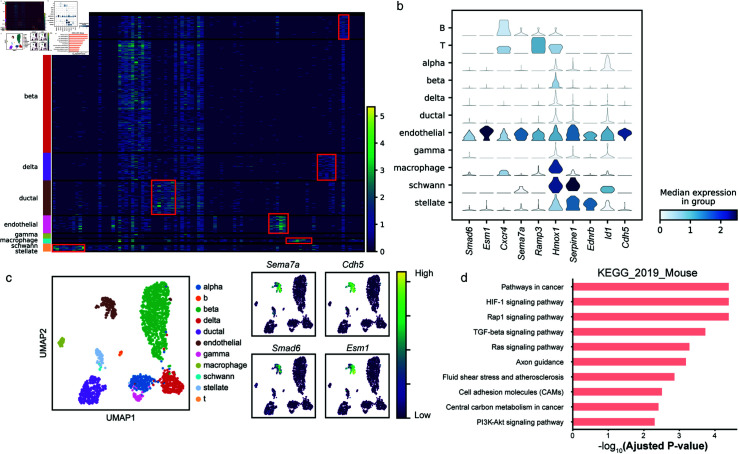
Critical gene analysis on Baron datasets. (a) Gene expression heatmaps of top 10 critical genes on Baron datasets. Each row represents a cell, and the colored bars on the left correspond to different cell types. Each column represents one critical gene, these critical genes are top 10 critical genes of each predict cell type results. (b) Dot plot of gene expression value for top 10 critical genes in endothelial on Baron datasets. (c) UMAP visualization of Baron datasets based on latent representation generated by scTrans, including cell type and gene expression of four critical genes. (d) Top 10 KEGG analysis results among top 100 critical genes of endothelial in Baron datasets.

As shown in Fig 4A, the heatmap visualizes the expression of top 10 critical genes in endothelial cells as predicted in the Baron dataset, which revealed the association between these critical genes and specific cell types. We also observed the same results on other five datasets (MCA Brain, TMS Brain, Romanov, TMS Pancreas and MCA Pancreas), as detailed in the S8A Fig. We analyzed the top 10 critical genes of predicted endothelial cell (Fig 4B and 4C). Among these genes, *Cdh5* is a marker gene for endothelial cell [44], Esm1 (Endothelial Cell-Specific Molecule 1) represents a distinct endothelial marker [45], whereas *Sema7a* is implicated in processes such as differentiation and signaling within endothelial cells [46]. Furthermore, *Smad6* plays a crucial role in maintaining the barrier function of endothelial cells [47].

We conducted an in-depth analysis of the top 10 critical genes in alpha, beta, and delta cells within the Baron dataset, and visualized these top 10 critical genes across the three cell types using stacked violin plots, as shown in S9 Fig. Through this visualization, we were able to observe the correlations between cell types and their critical genes. Specifically, the top 10 critical genes of alpha and delta cells included their respective markers, *Irx1* and *Hhex*. However, within the top 10 critical genes of beta cells, we did not identify a specific beta cell marker. Therefore, we conducted a more in-depth exploration of the critical genes in beta cells. We utilized the gene weights of beta cell markers calculated by scTrans to further compute the ranking scores of these markers, and performed randomization tests to assess the importance of these markers. As shown in S10 Fig, the results of the randomization tests indicated that the markers in beta cells generally had higher ranking scores, and exhibited extremely significant differences compared to the ranking scores obtained through random assignment (with p<0.001).

To further explore the biological relevance of critical genes, we first conducted KEGG enrichment analysis to the top 100 critical genes of endothelial cells in Baron dataset. Among the most significant top 10 pathways, three of them were related to endothelial cell function (Fig 4D). Specifically, Rap1 signaling pathway is crucial for maintaining endothelial barrier function [48], TGF-*β* signaling pathway and fluid shear stress in endothelial cells are key factors that regulate atherosclerosis [49,50]. Similarly, we applied pathway enrichment analysis to endothelial cells in MCA Pancreas and TMS Pancreas datasets, top 10 pathways were shown in S8B Fig. The Rap1 signaling pathway was one of the top 10 important pathways TMS Pancreas datasets. Additionally, we observed the expression of focal adhesion in MCA Pancreas and TMS Pancreas, which also played an important role in endothelial barrier function [51]. In TMS Pancreas, we also observed regulation of actin cytoskeleton, which significantly contributes to the regulation of endothelial barrier function [52]. We screened the endothelial cell enrichment analysis results from each dataset, selecting pathways with significant p-values less than 0.05 for comparison. By plotting Venn diagrams, as shown in S8C Fig, we demonstrated the overlapping signal pathways present in the same endothelial cells from the KEGG analysis results. The results showed that there were up to 5 signal pathways that were common across at least two datasets. Additionally, across all datasets, we identified a commonly expressed signal pathway, Focal adhesion, which had been previously mentioned in relation to endothelial barrier function.

To further explore the role of critical genes in endothelial cells, we conducted Reactome pathway enrichment analysis and GO enrichment analysis on the endothelial cells in the Baron dataset, GO enrichment analysis covered three categories: biological processes, cellular components, and molecular functions. As shown in S11A Fig, in the enrichment analysis results from Reactome, we found pathways associated with endothelial cells, such as Response of endothelial cells to shear stress.s And in all three categories of GO enrichment analysis results, we found signaling pathways closely related to endothelial cell activities. For instance, in biological processes, we identified regulation of endothelial cell migration and blood vessel endothelial cell migration; in cellular components, we found collagen-containing extracellular matrix; and in molecular functions, we discovered vascular endothelial growth factor receptor activity.

In conclusion, sTrans identified informative genes via attention mechanisms, which can be marker genes for distinguishing cell types or participate in important signal pathways of cells.

### scTrans generates high quality latent representation for clustering and trajectory analysis

We investigated the ability of scTrans in generating latent representations of single cells, which is crucial for downstream tasks such as clustering analysis and trajectory inference. The latent representations were extracted using scTrans trained on reference datasets. We applied K-Means to cluster latent representation, and used three metrics,the average silhouette width (ASW), adjusted rand index (ARI) and normalized mutual information (NMI), to evaluate the cluster results. Higher values in these three evaluations indicate better clustering effects. We compared scTrans with scSemiGAN [24] and scDeepSort [32], and two unsupervised clustering methods, scDeepCluster [53] and DESC [54], one semi-supervised clustering method, scDCC [55].

**Fig 5:**
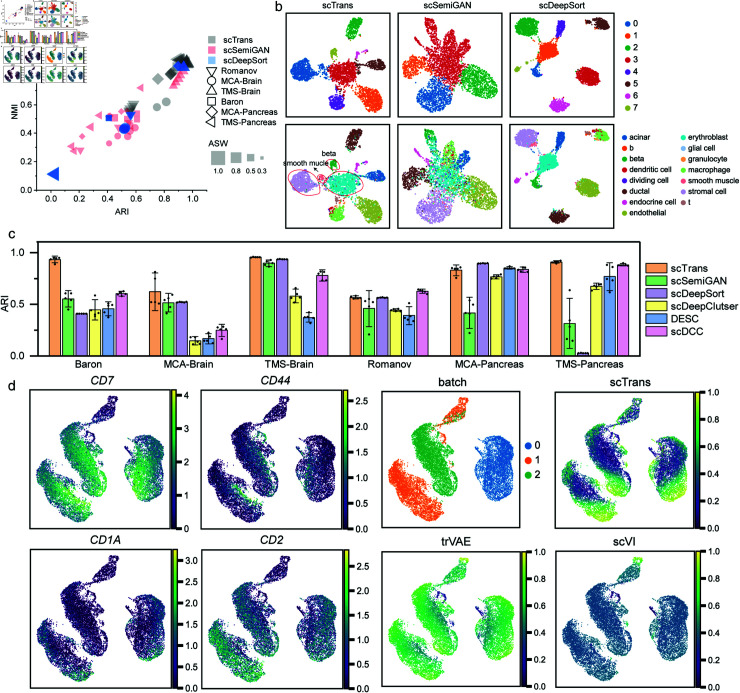
Latent representation quality analysis. (a) The ARI, NMI and ASW evaluation metrics were calculated for the clustering results of latent representation generated by scTrans, scSemiGAN and scDeepSort, with each datasets running 5 times. The y-axis represents NMI, the x-axis represents ARI, different shapes represent different datasets, and shape size represents ASW. (b) The six graphs showed the UMAP visualization of latent representation on MCA Pancreas dataset generated by scTrans, scSemiGAN, and scDeepSort, including K-Means clustering results and true cell type. (c) Comparison of the clustering results ARI of all methods in mouse brain and mouse pancreas datasets. Error bars were based on mean and 95*%* confidence. (d) UMAP visualization results showed T cell development dataset, including gene expression variations at different developmental stages and batch information of three donors in the datasets. And pseudo time inference results were shown based on latent representations generated by scTrans, trVAE, and scVI methods.

As shown in Fig 5A, in comparison with scSemiGAN and scDeepSort, scTrans achieved better performance in most datasets (also see in S12 Table). Visualization results in Fig 5B and S12 Fig also demonstrated scTrans’ ability to construct a high-quality latent representation, effectively clustering and distinguishing different cell types. Specifically, in MCA Pancreas, erythroblast was an unknown cell type for model trained based on TMS Pancreas and Baron datasets, but scTrans clustered erythroblast and achieved a clear separation. scSemiGAN mixed erythroblast with other cell. scDeepSort clustered erythroblast cells well, but struggled to separate smooth muscle cells from stromal cells. In other datasets, scSemiGAN intermixed brain pericyte and endothelial cells in TMS Brain, pp and alpha cells in TMS Pancreas, while scTrans could distinguish these cell types (S12C and S12D Figs). In MCA Brain, TMS Pancreas, and Baron datasets, scDeepSort generated notably non-compact cell clusters, and only divided cells into two cluster even under the best ASW conditions (S12B, S12D and S12E Figs). scTrans produced a compact latent representation, and achieved better ARI and NMI compared to scDeepSort in these three datasets (S12 Table).

When comparing clustering methods, scDeepCluster, DESC, and scDCC achieved higher scores in ASW, while scTrans demonstrated better performance in ARI and NMI (Fig 5B and S13 Fig). As Fig 5B shows, scTrans achieved the highest ARI on 4 out of 6 datasets and the best NMI on 3 out of 6 datasets. Notably, scTrans outperformed the second-best method scDCC by 55*%* in ARI and 11.3*%* in NMI on Baron datasets, achieving ARI and NMI of 0.93 and 0.89, respectively (S12 Table).

scDeepCluster and DESC tended to divide a single cell type into multiple clusters and mixed different cell types across most datasets (S14A and S14B Figs). Although scDCC benefited from using true labels for 10*%* of cells and had prior knowledge of the number of clusters, it struggled to accurately separate cells types with low counts (S14C Fig). scTrans generated a better latent representation, maintaining compact and well-separated clusters for each cell type, and effectively identified cell types with small counts in the latent representation, such as GABAergic, hypothalamic ependymal, and schwann cells in the MCA Brain datasets (S12B Fig).

To further demonstrate the strong feature extraction ability of scTrans, we used two cell development datasets: human T cells and mouse dendritic cell (DC) cells for trajectory analysis [29,30]. We trained scTrans on the PBMC160k dataset, then extracted latent representation from T cell development datasets, which consisted of three batches of Thymus. The datasets provided differentially expressed genes (DEG) at different developmental stages (S13 Table). Fig 5D shows batch effects and the expression of DEG in this datasets.

We compared scTrans with two batch correct methods: scVI and trVAE. All the three methods effectively intergrated data from different batches and preserved one unique cell clusters in batch 1 (S15 Fig). Leiden algorithms were performed for clustering the latent representation generated by various methods, and then the diffusion pseudotime method was used to infer cell trajectories. As shown in Fig 5D, and S15B and S15C Figs, scVI and trVAE erroneously identified the unique cell clusters in batch 1 as the final developmental stage, which did not match the true development stage of T cells. The pseudo time inferred by scTrans was more consistent with the variation of DEG (S15A Fig). We plotted the variations of DEG based on pseudo time inferred by three methods, and the variations of DEG in scTrans were most consistent with the stage of cell development (S15D Fig).

We also conducted cell trajectory analysis on the mouse DC development datasets. The developmental stage within this dataset were delineated as follows: macrophage dendritic cell progenitor (MDP), common DC progenitor (CDP), and pre-DC. We utilized datasets derived from 31 tissues in the MCA, encompassing total of 194,399 cells for training, and generated latent representation of the DC development dataset for cell trajectory analysis. We compared with two unsupervised clustering methods, DESC and scDeepCluster.

Compared with the other two methods, scTrans presented correct pseudotime result and a linear cell development trajectory (S16A Fig). scDeepCluster separated cells from different developmental stages and mixed some CDP and pre-DC cells in latent representation, thereby incorrect inference of pseudo time (S16B Fig). DESC inferred incorrect pseudo time because it mixed cells from various developmental stages (S16C Fig). Pre-dendritic cells (pre-DC) can be classified into three distinct types based on the differential expression of the genes *Ly6c2* and *Siglec-H*: *Siglec-H*-negative cells, *Ly6c*-positive *Siglec-H*-negative cells, and *Ly6c*-negative *Siglec-H*-negative cells [30]. Notably, only the scTrans method was able to observe the differences in the expression of these two genes within pre-DC.

In summary, we used scTrans trained on datasets with over 100,000 cells to extract latent representation from human T cell development and mouse DC cell development datasets, and based on these latent representation, we inferred accurate cell development trajectories. Through these two trajectory analysis experiments, we demonstrated the powerful feature extraction ability of scTrans.

### scTrans corrects batch effects and identifies cell subtypes in PBMC45k

To further evaluate the performance of scTrans, we utilized the PBMC160k datasets, which include cell subtype annotation results, as our reference dataset for training. Then we performed trained scTrans on PBMC45k datasets.

**Fig 6:**
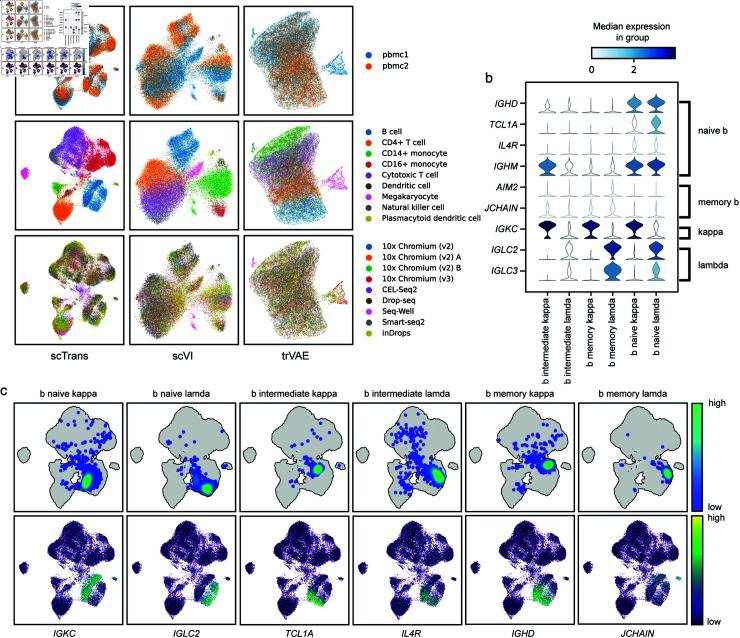
Batch correction and cell subtypes identification in PBMC45k. (a) Umap visualization results of latent representation generated by scTrans, scVI and trVAE, including donors, cell types and sequencing technology. (b) The expression of marker genes in six B cell subtypes. (c) The density of B cell subtypes and the expression of marker genes in latent representation generated by scTrans.

As Fig 6A shows, scTrans corrected batch effects in PBMC45k. We compared with two batch correction methods, scVI [56] and trVAE [57] by using cell type ASW and batch ASW evaluation metrics. Cell type ASW is used to measure biological conservation, and batch ASW is used to measure batch correction, higher values indicate better biological conservation and batch correction. scVI and trVAE achieved higher batch ASW than scTrans, but lower on cell type ASW (S17A Fig). Compares with these two methods, scTrans performed better in biological conservation, which was more clear in distinguishing different cell types when correct batch effects.

We compared scTrans with scSemiGAN and TOSICA in annotation task, both of which can be run on large-scale datasets in limited RAM. The heat map showed that the annotation results of scTrans are more accurate than TOSICA and scSemiGAN in PBMC45k (S17B Fig). For example, plasmacytoid dendritic cell, CD14+ and CD16+ monocyte were accurately identified by scTrans, with 93*%*, 81*%*, and 90*%* of cells correctly annotated. TOSICA and scSemiGAN displayed a lower rate in these tree cell types, TOSICA had 74*%*, 74*%* and 82*%*, and scSemiGAN had 53*%*, 71*%* and 71*%*. scTrans annotated 99*%* of megakaryocyte as platelet, a possible reason is that platelets are anucleate cytoplasmic discs derived from megakaryocytes [58], they have similarities in gene expression.

To demonstrate that scTrans correctly identified cell subtypes in PBMC45K, we took B cells as an example. According to developmental stages and immunoglobulin types, B cells were annotated into six subtypes of cells: naive kappa, intermediate kappa, memory kappa, naive lamda, intermediate lamda and memory lamda. We applied attention mechanism to extract top 10 critical genes in these 6 subtypes cell clusters for analysis. We found two genes, *IGHD* and *TCL1A* (Fig 6B and 6C), were highly expressed in naive B cells, which are marker gene of naive B cell [59,60]. The specific genes for immunoglobulin lamda and kappa are *IGLC* and *IGKC*, respectively. We further analyzed 6 cell subtypes based on kappa and lambda specific genes, as shown in Fig 6B, B cells annotated as kappa showed significant high expression of *IGKC*, while B cells annotated as lambda showed high expression of *IGLC2* and *IGLC3*. According to the expression of these three genes, B cell were divided into multi clusters, as shown in Fig 6C. For different developmental stages of B cell subtypes, we analyzed marker genes *TCL1A*, *IL4R*, *IGHD* and *IGHM* for naive B cell, and *AIM2* and *JCHAIN* for memory B cell [59,60]. As shown in Fig 6B and 6C, *TCL1A*, *IGHD* and *IGHM* were highly expressed in naive B cells, while *AIM2* and *JCHAIN* in memory B cells were also highly expressed compared to naive B cells, and intermediate B cells connected naive and memory B cells in the latent representation.

Dendritic cells in PBMC45K were also identified by scTrans as two subtypes of cells, cdc1 and cdc2. We extracted the top 10 critical genes in the cdc1 and cdc2 cell clusters, and found two critical genes, *CLEC10A* and *FCER1A*, which were marker genes for the cdc2 cell type (S17D Fig). Then we analyzed marker gene *THBD* and *CLEC9A* in cdc1, and *CD1C*, *FCER1A* and *CLEC10A* in cdc2 [61,62]. As S17E Fig shows, the marker genes *CD1C*, *FCER1A*, and *CLEC10A* were highly expressed in cdc2, while cdc1 shows relatively higher expression of the *CLEC9A* marker gene compared to cdc2. The distribution differences of these marker genes indicate that dendritic cells in PBMC45k are consist of two cell subtypes, cdc1 and cdc2.

In summary, we demonstrated that scTrans can effectively utilize high-precision cell type annotation results to identify cell subtypes from other datasets.

## Discussion

In this study, we developed scTrans, a transformer-based cell type annotation tool. scTrans maps genes to informative embeddings and uses sparse attention to extract features from non-zero gene embeddings, enabling accurate and rapid cell type annotation. Firstly, we applied scTrans on 31 mouse tissues from Mouse Cell Atlas, and scTrans achieved the highest accuracy on 30 tissues. Secondly, scTrans reduced computational burden and RAM consumption, driven by only using non-zero genes, allowing us to quickly run datasets of nearly one million cells with only 40GB of memory. Thirdly, scTrans converted genes into informative embeddings and included all genes during training, ensuring maximum overlap between query and reference datasets, achieving batch insensitive cell type annotation. Meanwhile, scTrans extracted high-quality latent representation from novel datasets, and achieved accurate cell clustering, which demonstrated the powerful feature extraction ability of scTrans. We also applied scTrans for cell trajectory analysis of human T cells and mouse DC cells, scTrans accurately inferred pseudo time consistent with T cell and DC cell development processes. Additionally, scTrans can identify informative genes via attention mechanism, which can be marker genes corresponding to cell types or play important roles in cellular processes.

There are still areas for improvement and further research directions for this study. Enhancing the quality of gene representation and improving the model’s generalization ability remain key challenges. we have not yet considered the impact of hierarchical relationships in cell development on misclassification loss, we plan to address this issue by introducing solutions such as weighted cross-entropy loss or constructing a hierarchical classification network in the future. Additionally, scTrans could be applied to the analysis of other omics data, such as spatial transcriptomics or multi-omics integration.

## Materials and methods

### Preprocess

In preprocess step, we first obtain the gene index ($g^{index}$) and gene expression values ($g^{value}$) of non-zero genes within each cell through the following description:


$$\begin{array}{ccc} g_{i}^{index}=filter(I_{i},M_{i}) & & \end{array}$$ (1)

$$\begin{array}{ccc} g_{i}^{value}=filter(E_{i},M_{i}) & & \end{array}$$ (2)

$I_{i}$ is all gene index of cell i, $E_{i}$ is all gene expression of cell i, $M_{i}$ is the mask of cell i, where $M_{i}=(E_{i}!=0)$. *filter* is a function that extracts the masked index and value from $I_{i}$ and $E_{i}$ based on the given mask $M_{i}$, and stores them as two lists $g_{i}^{index}$ and $g_{i}^{value}$.

Then we process the gene expression of each cell by using mean scaling, which is dividing each gene expression by the average non-zero gene expression within the cell, as shown in Equation 3.


$$\begin{array}{ccc} g_{i}^{value}=g_{i}^{value}∕Mean(g_{i}^{value}) & & \end{array}$$ (3)

*Mean* represents calculating the average expression of **$g_{i}^{value}$**.

### Gene embedding

Due to the varying number of non-zero expressed genes in each cell, we pad $g^{index}$ within a batch into a matrix as model inputs.


$$\begin{array}{ccc} G_{index} & =Padding(g_{1}^{index},...,g_{B}^{index}) & \end{array}$$ (4)

$$\begin{array}{ccc} G_{value} & =Padding(g_{1}^{value},...,g_{B}^{value}) & \end{array}$$ (5)

$G_{index}\in R^{B\times M}$ and $G_{value}\in R^{B\times M}$ are the matrix in a batch obtained from the padding operation, which is to add padding symbols at the end of the $g^{index}$ and $g^{value}$ list. Specifically, we add the padding index in $G^{index}$, and add zero value in $G^{value}$. $g_{i}^{index}$ and $g_{i}^{value}$ is the non-zero gene index and value of i-th cell. *B* is the batch size, and *M* is the longest length of the gene index list in a batch

Then we map these gene index to embedding by an embedding layer, and encoded these gene embeddings with gene value through dot multiplication. In embedding layer, we add an optional dropout layer, which is used only when there is a strong batch effect between datasets.


$$\begin{array}{ccc} G_{embedding}=Embedding(G_{index})\cdot G_{value} & & \end{array}$$ (6)

$G_{embedding}\in R^{B\times M\times D}$ stores the non-zero gene embeddings of all cells within a batch, *D* is the length of gene embedding.

### Embedding initialize

In the embedding initialization phase, we start with matrix $X\in {\mathbb{R}}^{⋗\times ⋉}$, which has been normalized, where m represents the number of cells and n represents the number of genes. We then transpose *X* to obtain the gene-cell matrix $G\in {\mathbb{R}}^{n\times m}$. Following this, we perform PCA on matrix *G*. Through this process, we extract the top *d* principal components, resulting in a dimension-reduced matrix, known as the gene embedding $G_{embedding}\in {\mathbb{R}}^{n\times d}$. In the subsequent pre-training process, all embeddings will be updated. For large-scale datasets, we will sample some cells to construct a small gene-cell matrix for initialization embedding.

### Attention aggregation

The attention aggregation consists of multiple attention blocks, each with $X_{q}$, $X_{k}$, and $X_{v}$ embedding as query, key and value inputs and one output *Z*, as described $Z=Block(X_{q},X_{k},X_{v})$.

The input of the first block aggregates gene information by using a $CLS_{embedding}$ as the query and $G_{embedding}$ as the key and value. $CLS_{embedding}$ is a trainable vector, of the same size d as the gene embedding, which is randomly initialized using a normal distribution. During the backward propagation process, $CLS_{embedding}$ will be updated based on the computed gradients.


$$\begin{array}{ccc} Z^{1}=Block^{1}(CLS_{embedding},G_{embedding},G_{embedding}) & & \end{array}$$ (7)

The subsequent block aggregates gene information again by using the output of previous block as a query and $G_{embedding}$ as a key and value. The output of last block is the output of encoder, which is also the latent representation of cells.


$$\begin{array}{ccc} Z^{N}=Block^{N}(Z^{N-1},G_{embedding},G_{embedding});N>1 & & \end{array}$$ (8)

The specific information aggregation process within each block is as follows.

First, perform linear transformation on the $X_{q}$, $X_{k}$ and $X_{v}$ matrices to obtain three matrices *Q*, *K*, and *V*, the formula is as follows:


$$\begin{array}{ccc} Q_{h}=X_{q}W_{h}^{Q},K_{h}=X_{k}W_{h}^{K},V_{h}=X_{v}W_{h}^{V};h=1,...,H & & \end{array}$$ (9)

where *H* is the number of heads, *h* represents the h-th head, $W^{Q}$, $W^{K}$ and $W^{V}$ are trainable parameter matrices.

Aggregating information of *Q*, *K*, and *V* through multi head attention mechanism. The formula is as follows:


$$\begin{array}{ccc} hidden_{h}=softmax\left(\frac{Q_{h}K_{h}^{T}}{\sqrt{d}}\right)V_{h},h=1,...,H & & \end{array}$$ (10)

$hidden_{h}$ represents the aggregation result of i-th head, **d** is the length of embedding. We concatenated the outputs of each attention head together and perform a dense layer to further extract the features of each attention head.


$$\begin{array}{ccc} O=Concact(hidden_{1},...,hidden_{H})W^{O} & & \end{array}$$ (11)

*O* is the concatenation of the aggregation results of all attention heads, $W^{O}$ is trainable parameters in dense layer.

Next, non-linear features are extracted through feedforward layer (FFN). Specifically, it is a two-layer MLP, which maps data to high-dimensional space and then to low-dimensional space, extracting deeper features *Z*, describe as *Z* = *MLP* ( *O* ) .

Add&Norm(*AN*) combines residual connections with LayerNorm to accelerate model convergence and solve gradient vanishing problems. This module is used after both multi head attention and FFN, the calculations are as follows:


$$\begin{array}{ccc} O & =AN(X_{q},O)=X_{q}+LayerNorm(O) & \end{array}$$ (12)

$$\begin{array}{ccc} Z & =AN(O,Z)=O+LayerNorm(Z) & \end{array}$$ (13)

where $X_{q}$ is the input of block, *O* is the result of attention aggregation, and *Z* is the output of FFN layer and block.

### Contrastive pre-training

We use gene-cell matrix for PCA to obtain an initialized gene embedding, but the embedding obtained through PCA may not necessarily be applicable to the target task. And in large-scale data, we will sample part of cells for initialization embedding, which possibly obtained gene embedding not suitable for model. Therefore, we introduce contrastive learning for pre-training to continue optimizing gene embedding.

We perform random mask on $G_{index}$ of each cell in a batch for data augmentation, constructed positive samples $G_{index}^{{}^{\prime }}$. The random mask is to randomly mask gene index, all gene index have a 15*%* chance to be masked. The original sample $G_{index}$ and its positive samples $G_{index}^{{}^{\prime }}$ within a batch are positive pairs, while the original sample and other samples within a batch are negative pairs. Latent representation of positive and negative sample pairs are extracted via shared weights encoder. Then, a project head layer is applied to extract nonlinear features of latent representation, which consists of two-layer MLP. The formula is $s_{i}=MLP(z_{i})$, $z_{i}$ is output of encoder for the i-th cell, $s_{i}$ is the nonlinear feature obtained from $z_{i}$ through the project head.

Then, we apply infoNCEloss to constrain the output of project head layer, maximizing the similarity between positive pairs while minimizing the similarity between negative pairs.


$$\begin{array}{ccc} sim(s_{i},s_{j})=s_{i}^{T}s_{j}∕∥s_{i}∥∥s_{j}∥ & & \end{array}$$ (14)

$sim(s_{i},s_{j})$ calculates the cosine similarity between $s_{i}$ and $s_{j}$.


$$\begin{array}{ccc} L_{i,i^{+}}=log\frac{exp(sim(s_{i},s_{i^{+}})∕\tau )}{\overset{2B}{\underset{k=0}{\sum }}I(k\neq i)exp(sim(s_{i},s_{k}∕\tau ))} & & \end{array}$$ (15)

$L_{i,i^{+}}$ represents the contrastive loss in i-th cell in a batch, *B* is the batch size, $s_{i}$ and $s_{i^{+}}$ are positive pairs,  ∥  is a hyperparameter with a value of 0.1.


$$\begin{array}{ccc} L_{CL}=\frac{1}{2B}\overset{B}{\underset{i=0}{\sum }}[L_{i,i^{+}}+L_{i^{+},i}] & & \end{array}$$ (16)

$L_{CL}$ is the average contrastive loss of all cells in a batch, during the pre-training, the model is trained based on $L_{CL}$ loss function.

### Fine-tuning

In the fine-tuning stage, the pre-trained encoder is used to extract latent representation of cells, and a dense layer is used to map latent representation to cell types, *p* = *Dense* ( *Z* ) , *p* is predicted probability of cell type. During fine-tuning, we use cross entropy loss as the optimization objective.


$$\begin{array}{ccc} L_{CE}=-\frac{1}{B}\overset{B}{\underset{i=0}{\sum }}y_{i}log(p_{i}) & & \end{array}$$ (17)

*y* represents the ground truth and *p* is the predicted probability, $L_{CE}$ is the cross entropy loss within a batch.

### Ablation experiment

To highlight the importance of each step in scTrans, we conducted ablation experiments to assess the impact of PCA embedding initialization, pre-training, embedding dropout, mean scaling, and model structure. Details see in S1 Text.

### Hardware conditions

All methods are run on the same machine and calculate the running time. The hardware configuration is as follows: GPU: RTX2080 Ti(11GB), CPU: 12 vCPU Intel(R) Xeon(R) Platinum 8255C CPU @ 2.50GHz, RAM:40GB.

### Evaluation metric

Accuracy and f1-macro are calculated for comparing annotation performance. Accuracy is used to measure the proportion of correctly annotated cell types by the model. Considering the imbalance of cell numbers between cell types, f1-macro is introduced. F1-macro is a weighted average f1-score based on categories, with the same weight for each category. Compared to accuracy, F1-macro better reflects the model’s performance in imbalanced cell type datasets.

ARI, NMI, and ASW are used to evaluate clustering results. ARI and NMI is used to measure the similarity between clustering results and real categories, with larger values indicating greater similarity between clustering results and real categories. The range of ARI values is -1 to 1, and the range of NMI is 0 to 1, with larger values indicating better clustering results. ASW is used to measure the compactness of cells in their respective clusters and their separation from other clusters. The range of contour coefficient values is between -1 and 1, and the larger the value, the better the clustering effect. The five evaluation metrics: accuracy, f1-macro, ARI, NMI and ASW all calculated by using implementation of scikit-learn.

Cell type ASW and batch ASW are used to evaluate biological conservation and batch correction. Cell type ASW is calculated as the equation:


$$\begin{array}{ccc} cell\;type\;ASW=(ASW_{C}+1)∕2 & & \end{array}$$ (18)

$ASW_{C}$ is an ASW calculated based on cell type cluster. The cell type ASW range is from 0 to 1, and a larger value indicates a better biological conservation. Batch ASW is to evaluate batch mixing and calculate as the equation:


$$\begin{array}{ccc} & s_{batch}(i)=|s(i)| & \end{array}$$ (19)

$$\begin{array}{ccc} & batch\;ASW=\frac{1}{\left|M\right|}\underset{j\in M}{\sum }\frac{1}{\left|C_{j}\right|}\underset{i\in C_{j}}{\sum }1-s_{batch}(i) & \end{array}$$ (20)

$s_{batch}(i)$ is the absolute silhouette width on batch labels of each cell i. M is the set of unique cell labels. $C_{j}$ is the set of cells with label j and $\left|C_{j}\right|$ is the number of cells in the set. The range of Batch ASW is from 0 to 1, higher batch ASW indicates better batch correction results. We used the scib implementation of to compute cell type ASW and batch ASW.

### Critical gene analysis

We first obtain the attention weights of each gene in each cell through the attention mechanism in scTrans. Then, based on the predicted cell type clusters, sum up the gene weights in each cluster, calculate as follow:


$$\begin{array}{ccc} w_{j}^{k}=\underset{i\in C_{k}}{\sum }attn_{ij} & & \end{array}$$ (21)

$w_{j}^{k}$ is the attention weight of the j-th gene in the k-th cell cluster. $C_{k}$ is the set of cells in cluster k. $attn_{ij}$ is the attention weight of the j-th gene in the i-th cell. Finally, genes are sorted from large to small based on the calculated weights in each cell cluster, and selected for further analysis.

**Top critical gene analysis.** For mouse pancreas and mouse brain, we selected top 10 critical genes for drawing heatmaps to show the different of genes and cells.

**KEGG Enrichment Analysis.** Kyoto Encyclopedia of Genes and Genomes (KEGG) signaling pathway enrichment analysis is performed by using the gseapy package in Python. We first use the attention mechanism in scTrans to extract the attention weights of cell clusters, then selected top 100 critical genes as input of KEGG enrichment analysis.

### Clustering methods

In clustering analysis, the latent representation generated by all methods are clustered by using the K-Means algorithm implemented by python package scikit-learn. We searched for the optimal number of clusters for best ASW through grid search from 2 to 15, and use it as the final clustering parameter for K-Means. In human T cell development datasets analysis, we use the Leiden algorithm in the scanpy package for clustering, with all parameters set to default values.

### Methods comparison

We compared scTrans with pre-trained model Concerto, two semi supervised methods scSemiGAN, itclust, two supervised methods scDeepSort and TOSICA in cell type annotation task. And in clustering analysis, we compared with scSemiGAN, scDeepSort, tow unsupervised clustering methods scDeepCluster, DESC and one semi-supervised clustering method scDCC. For cell trajectory inference task, we compared scTrans with two batch correct methods scVI and trVAE using the human T cell development dataset, and with scDeepCluster and DESC using the mouse DC cell development dataset. The description and parameter details of each methods are provided in the S1 Text.

## Supporting information

S1 Fig The details of encoder architecture.
(DOCX)

S2 Fig Accuracy and f1-macro in large scale annotation task at different train rate.
(DOCX)

S3 Fig The variation in annotation performance of scTrans across simulated datasets with different sequencing depths.
(DOCX)

S4 Fig Batch effects between two donors and cross technology annotation task results in PBMC45k datasets.
(DOCX)

S5 Fig F1-macro of cross batch annotation results in mouse brain and mouse pancreas datasets.
(DOCX)

S6 Fig Sankey diagram of annotation results in cross datasets multi reference annotation task.
(DOCX)

S7 Fig UMAP visualization results of Cell types and Tnc gene expression in MCA Brain dataset.
(DOCX)

S8 Fig Critical gene analysis results.
(**A**) Critical genes heatmap of MCA Brain, TMS Brain, Romanov, MCA Pancreas and TMS Pancreas datasets. (**B**) KEGG analysis results of endothelial cells in MCA Pancreas and TMS Pancreas datasets. (**C**) Venn diagrams illustrating the enrichment analysis results of endothelial cells across three datasets.
(DOCX)

S9 Fig This figure presents the stacked violin plots of the top 10 critical genes for six cell types in the Baron dataset.
(**A–C**) Respectively, the stacked violin plots for alpha, beta, delta.
(DOCX)

S10 Fig This figure presents a comparison of marker weight ranking scores extracted by scTrans versus those generated randomly through randomization testing.
(DOCX)

S11 Fig Reactome and Go enrichment analysis results of top 100 critical genes of endothelial cells in Baron dataset.
(DOCX)

S12 Fig UMAP visualization of latent representations generated by scTrans, scSemiGAN and scDeepSort for mouse brain and mouse pancreas datasets.
(**A–F**) UMAP visualization about latent representation in MCA Pancreas, MCA Brain, TMS Brain, TM Pancreas, Baron and Romanov datasets.
(DOCX)

S13 Fig ASW and NMI of clustering results in mouse Brain and mouse Pancreas datasets.
(DOCX)

S14 Fig UMAP visualization of latent representations generated by scDeepCluster, DESC and scDCC for mouse brain and mouse pancreas datasets.
(**A–C**) UMAP visualization of latent representation in mouse Brain and mouse Pancreas datasets generated by scDeepCluster, DESC and scDCC.
(DOCX)

S15 Fig UMAP visualization of T cell development analysis results.
(**A–C**) UMAP visualization of latent representations generated by scTrans, scVI and trVAE for T cell development datasets, including clustering results, dpt pseudotime inference results, and expression of DEG in development stage. (**D**) The expression variations of four specific genes based on the pseudo time results inferred from scTrans, scVI, and trVAE.
(DOCX)

S16 Fig UMAP visualization of mouse DC cell development analysis results.
(**A–C**) UMAP visualization results of latent representation generated by scTrans, scDeepCluster and DESC, including cell type, inferred pseudo time result, two gene expression, Ly6c2 and Siglec-H.
(DOCX)

S17 Fig Analysis of batch correction and cell subtype annotation results in PBMC45k dataset.
(**A**) Cell type ASW and batch ASW results computed based on scTrans, scVI and trVAE. (**B**) Annotation heatmap results of scTrans, scSemiGAN and scDeepSort in PBMC45k by using PBMC160k dataset as reference. (**C**) Expression of top 10 critical genes in B cell subtypes identified by scTrans. (**D**) Expression of top 10 critical genes in dendritic cell subtypes identified by scTrans. (**E**) Expression of marker genes in dendritic cell subtypes.
(DOCX)

S1 Table The number of cells and cell types of 31 tissues datasets in MCA datasets.
(DOCX)

S2 Table Accuracy of annotation results for 31 tissues in MCA datasets.
(XLSX)

S3 Table F1-macro score of annotation results for 31 tissues in MCA datasets.
(XLSX)

S4 Table The mean and standard deviation of accuracy and f1-macro based on 31 tissues in MCA datasets at different train rate.
(DOCX)

S5 Table Performance evaluation of scTrans trained with different levels of cell type annotations.
(DOCX)

S6 Table Comparison results with large models.
(DOCX)

S7 Table Comparison results of PCA embedding initialization and large model embedding initialization.
(DOCX)

S8 Table Details of PBMC45k.
(DOCX)

S9 Table Cell type details of three mouse brain and three mouse pancreas datasets.
(DOCX)

S10 Table Accuracy and fi-macro of annotation results in single reference and multi reference task on PBMC45K datasets.
(DOCX)

S11 Table Accuracy and fi-macro of annotation results in single reference and multi reference task on mouse brain and mouse pancreas datasets.
(DOCX)

S12 Table Clustering performance results in mouse brain and mouse pancreas datasets.
(DOCX)

S13 Table Expression of differentially expressed genes in T cell development stages.
(DOCX)

S1 Text This file contains experimental details, parameter settings for comparison methods, ablation experiment analysis on the model structure, and simulating experiments details.
(DOCX)

## References

[1] Zhao S, Hong CK, Myers CA, Granas DM, White MA, Corbo JC, et al. A single-cell massively parallel reporter assay detects cell-type-specific gene regulation. Nat Genet 2023;55(2):346–54. doi: 10.1038/s41588-022-01278-7 36635387 PMC9931678

[2] Wang Y, Lian B, Zhang H, Zhong Y, He J, Wu F, et al. A multi-view latent variable model reveals cellular heterogeneity in complex tissues for paired multimodal single-cell data. Bioinformatics. 2023;39(1):btad005. doi: 10.1093/bioinformatics/btad005 PMC9857983 36622018

[3] Jia Q, Chu H, Jin Z, Long H, Zhu B. High-throughput single-cell sequencing in cancer research. Signal Transduct Target Ther 2022;7(1):145. doi: 10.1038/s41392-022-00990-4 35504878 PMC9065032

[4] Evrony GD, Hinch AG, Luo C. Applications of single-cell DNA sequencing. Annu Rev Genomics Hum Genet. 2021;22:171–97 doi: 10.1146/annurev-genom-111320-090436 PMC8410678 33722077

[5] Clarke ZA, Andrews TS, Atif J, Pouyabahar D, Innes BT, MacParland SA, et al. Tutorial: guidelines for annotating single-cell transcriptomic maps using automated and manual methods. Nat Protoc 2021;16(6):2749–64. doi: 10.1038/s41596-021-00534-0 34031612

[6] Kiselev VY, Yiu A, Hemberg M. scmap: projection of single-cell RNA-seq data across data sets. Nat Methods 2018;15(5):359–62. doi: 10.1038/nmeth.4644 29608555

[7] de Kanter JK, Lijnzaad P, Candelli T, Margaritis T, Holstege FCP. CHETAH: a selective, hierarchical cell type identification method for single-cell RNA sequencing. Nucleic Acids Res 2019;47(16):e95. doi: 10.1093/nar/gkz543 31226206 PMC6895264

[8] Lieberman Y, Rokach L, Shay T. CaSTLe - Classification of single cells by transfer learning: Harnessing the power of publicly available single cell RNA sequencing experiments to annotate new experiments. PLoS ONE 2018;13(10):e0205499. doi: 10.1371/journal.pone.0205499 30304022 PMC6179251

[9] Ma Q, Xu D. Deep learning shapes single-cell data analysis. Nat Rev Mol Cell Biol 2022;23(5):303–4. doi: 10.1038/s41580-022-00466-x 35197610 PMC8864973

[10] Yang F, Wang W, Wang F, Fang Y, Tang D, Huang J, et al. scBERT as a large-scale pretrained deep language model for cell type annotation of single-cell RNA-seq data. Nat Mach Intell 2022;4(10):852–66. doi: 10.1038/s42256-022-00534-z

[11] Cui H, Wang C, Maan H, Pang K, Luo F, Duan N, et al. scGPT: toward building a foundation model for single-cell multi-omics using generative AI. Nat Methods 2024;21(8):1470–80. doi: 10.1038/s41592-024-02201-0 38409223

[12] Hao M, Gong J, Zeng X, Liu C, Guo Y, Cheng X, et al. Large-scale foundation model on single-cell transcriptomics. Nat Methods 2024;21(8):1481–91. doi: 10.1038/s41592-024-02305-7 38844628

[13] Wen H, Tang W, Dai X, Ding J, Jin W, Xie Y, et al. CellPLM: pre-training of cell language model beyond single cells. bioRxiv. preprint. 2023. doi: 10.1101/2023.10.03.560734

[14] Theodoris CV, Xiao L, Chopra A, Chaffin MD, Al Sayed ZR, Hill MC, et al. Transfer learning enables predictions in network biology. Nature 2023;618(7965):616–24. doi: 10.1038/s41586-023-06139-9 37258680 PMC10949956

[15] Chen Y, Zou J. GenePT: a simple but effective foundation model for genes and cells built from ChatGPT. bioRxiv. preprint. 2024. doi: 10.1101/2023.10.16.562533

[16] Hou W, Ji Z. Assessing GPT-4 for cell type annotation in single-cell RNA-seq analysis. Nat Methods 2024;21(8):1462–5. doi: 10.1038/s41592-024-02235-4 38528186 PMC11310073

[17] Yang M, Yang Y, Xie C, Ni M, Liu J, Yang H, et al. Contrastive learning enables rapid mapping to multimodal single-cell atlas of multimillion scale. Nat Mach Intell 2022;4(8):696–709. doi: 10.1038/s42256-022-00518-z

[18] Wolf FA, Angerer P, Theis FJ. SCANPY: large-scale single-cell gene expression data analysis. Genome Biol 2018;19(1):15. doi: 10.1186/s13059-017-1382-0 29409532 PMC5802054

[19] Satija R, Farrell JA, Gennert D, Schier AF, Regev A. Spatial reconstruction of single-cell gene expression data. Nat Biotechnol 2015;33(5):495–502. doi: 10.1038/nbt.3192 25867923 PMC4430369

[20] Li H, Brouwer CR, Luo W. A universal deep neural network for in-depth cleaning of single-cell RNA-Seq data. Nat Commun 2022;13(1):1901. doi: 10.1038/s41467-022-29576-y 35393428 PMC8990021

[21] Wang D, Hou S, Zhang L, Wang X, Liu P, Zhang Z. iMAP: integration of multiple single-cell datasets by adversarial paired transfer networks. Genome Biol 2021;22(1):63. doi: 10.1186/s13059-021-02280-8 33602306 PMC7891139

[22] Wang X, Wang J, Zhang H, Huang S, Yin Y. HDMC: a novel deep learning-based framework for removing batch effects in single-cell RNA-seq data. Bioinformatics 2021;38(5):1295–303. doi: 10.1093/bioinformatics/btab821 34864918

[23] Hu J, Li X, Hu G, Lyu Y, Susztak K, Li M. Iterative transfer learning with neural network for clustering and cell type classification in single-cell RNA-seq analysis. Nat Mach Intell 2020;2(10):607–18. doi: 10.1038/s42256-020-00233-7 33817554 PMC8009055

[24] Xu Z, Luo J, Xiong Z. scSemiGAN: a single-cell semi-supervised annotation and dimensionality reduction framework based on generative adversarial network. Bioinformatics 2022;38(22):5042–8. doi: 10.1093/bioinformatics/btac652 36193998

[25] Han X, Wang R, Zhou Y, Fei L, Sun H, Lai S, et al. Mapping the mouse cell atlas by microwell-seq. Cell. 2018;172(5):1091–1107.e17. doi: 10.1016/j.cell.2018.02.001 29474909

[26] Ding J, Adiconis X, Simmons SK, Kowalczyk MS, Hession CC, Marjanovic ND, et al. Systematic comparison of single-cell and single-nucleus RNA-sequencing methods. Nat Biotechnol 2020;38(6):737–46. doi: 10.1038/s41587-020-0465-8 32341560 PMC7289686

[27] Hao Y, Hao S, Andersen-Nissen E, Mauck WM, 3rd, Zheng S, Butler A, et al. Integrated analysis of multimodal single-cell data. Cell. 2021;184(13):3573–87.e29. doi: 10.1016/j.cell.2021.04.048 PMC8238499 34062119

[28] Oelen R, de Vries DH, Brugge H, Gordon MG, Vochteloo M, Ye CJ, et al. Single-cell RNA-sequencing of peripheral blood mononuclear cells reveals widespread, context-specific gene expression regulation upon pathogenic exposure. Nat Commun 2022;13(1):3267. doi: 10.1038/s41467-022-30893-5 35672358 PMC9174272

[29] Le J, Park JE, Ha VL, Luong A, Branciamore S, Rodin AS, et al. Single-cell RNA-seq mapping of human thymopoiesis reveals lineage specification trajectories and a commitment spectrum in T cell development. Immunity. 2020;52(6):1105–18.e9. doi: 10.1016/j.immuni.2020.05.010 PMC7388724 32553173

[30] Schlitzer A, Sivakamasundari V, Chen J, Sumatoh HRB, Schreuder J, Lum J, et al. Identification of cDC1- and cDC2-committed DC progenitors reveals early lineage priming at the common DC progenitor stage in the bone marrow. Nat Immunol 2015;16(7):718–28. doi: 10.1016/j.immuni.2020.05.01010.1038/ni.3200 26054720

[31] Chen T, Kornblith S, Norouzi M, Hinton G A simple framework for contrastive learning of visual representations. In: International Conference on Machine Learning; 2020.

[32] Shao X, Yang H, Zhuang X, Liao J, Yang P, Cheng J, et al. scDeepSort: a pre-trained cell-type annotation method for single-cell transcriptomics using deep learning with a weighted graph neural network. Nucleic Acids Res 2021;49(21):e122. doi: 10.1093/nar/gkab775 34500471 PMC8643674

[33] Chen J, Xu H, Tao W, Chen Z, Zhao Y, Han J-DJ. Transformer for one stop interpretable cell type annotation. Nat Commun 2023;14(1):223. doi: 10.1038/s41467-023-35923-4 36641532 PMC9840170

[34] Zappia L, Phipson B, Oshlack A. Splatter: simulation of single-cell RNA sequencing data. Genome Biol 2017;18(1):174. doi: 10.1186/s13059-017-1305-0 28899397 PMC5596896

[35] Leek JT, Scharpf RB, Bravo HC, Simcha D, Langmead B, Johnson WE, et al. Tackling the widespread and critical impact of batch effects in high-throughput data. Nat Rev Genet 2010;11(10):733–9. doi: 10.1038/nrg2825 20838408 PMC3880143

[36] Hicks SC, Townes FW, Teng M, Irizarry RA. Missing data and technical variability in single-cell RNA-sequencing experiments. Biostatistics 2017;19(4):562–78. doi: 10.1093/biostatistics/kxx053 PMC6215955 29121214

[37] Schaum N, Karkanias J, Neff NF, May AP, Quake SR, Wyss-Coray T, et al. Single-cell transcriptomics of 20 mouse organs creates a Tabula Muris. Nature 2018;562(7727):367–72. doi: 10.1038/s41586-018-0590-4 30283141 PMC6642641

[38] Romanov RA, Zeisel A, Bakker J, Girach F, Hellysaz A, Tomer R, et al. Molecular interrogation of hypothalamic organization reveals distinct dopamine neuronal subtypes. Nat Neurosci 2017;20(2):176–88. doi: https://doi.org/10.1038/nn.4462 27991900 10.1038/nn.4462 PMC7615022

[39] Baron M, Veres A, Wolock Samuel L, Faust Aubrey L, Gaujoux R, Vetere A, et al. A single-cell transcriptomic map of the human and mouse pancreas reveals inter- and intra-cell population structure. Cell Syst. 2016;3(4):346–60.e4. doi: 10.1016/j.cels.2016.08.011 PMC5228327 27667365

[40] Cheng FY, Fleming JT, Chiang C. Bergmann glial Sonic hedgehog signaling activity is required for proper cerebellar cortical expansion and architecture. Dev Biol 2018;440(2):152–66. doi: 10.1016/j.ydbio.2018.05.015 29792854 PMC6014626

[41] Yuasa S. Bergmann glial development in the mouse cerebellum as revealed by tenascin expression. Anat Embryol 1996;194(3):223–34. doi: 10.1007/BF00187133 8849669

[42] Allam A, Thomsen AR, Gothwal M, Saha D, Maurer J, Brunner TB. Pancreatic stellate cells in pancreatic cancer: in focus. Pancreatology 2017;17(4):514–22. doi: 10.1016/j.pan.2017.05.390 28601475

[43] Wang Z, Dong S, Zhou W. Pancreatic stellate cells: key players in pancreatic health and diseases (Review). Mol Med Rep 2024;30(1):109. doi: 10.3892/mmr.2024.13233 38695254 PMC11082724

[44] Paik DT, Tian L, Williams IM, Rhee S, Zhang H, Liu C, et al. Single-cell RNA sequencing unveils unique transcriptomic signatures of organ-specific endothelial cells. Circulation 2020;142(19):1848–62. doi: 10.1161/CIRCULATIONAHA.119.041433 32929989 PMC7658053

[45] Lassalle P, Molet S, Janin A, Van der Heyden J, Tavernier J, Fiers W, et al. ESM-1 is a novel human endothelial cell-specific molecule expressed in lung and regulated by cytokines. J Biol Chem 1996;271(34):20458–64. doi: 10.1074/jbc.271.34.20458 8702785

[46] Hong L, Li F, Tang C, Li L, Sun L, Li X, et al. Semaphorin 7A promotes endothelial to mesenchymal transition through ATF3 mediated TGF-*β*2/Smad signaling. Cell Death Dis. 2020;11(8):695. doi: 10.1038/s41419-020-02818-x PMC7442651 32826874

[47] Ruter DL, Liu Z, Ngo KM, X S, Marvin A, Buglak DB, et al. SMAD6 transduces endothelial cell flow responses required for blood vessel homeostasis. Angiogenesis 2021;24(2):387–98. doi: 10.1007/s10456-021-09777-7 33779885 PMC8206051

[48] Pannekoek WJ, Post A, Bos JL. Rap1 signaling in endothelial barrier control. Cell Adh Migr 2014;8(2):100–7. doi: 10.4161/cam.27352 24714377 PMC4049856

[49] Chen P-Y, Qin L, Li G, Wang Z, Dahlman JE, Malagon-Lopez J, et al. Endothelial TGF-*β* signalling drives vascular inflammation and atherosclerosis. Nat Metab. 2019;1(9):912–26. doi: 10.1038/s42255-019-0102-3 PMC6767930 31572976

[50] Souilhol C, Serbanovic-Canic J, Fragiadaki M, Chico TJ, Ridger V, Roddie H, et al. Endothelial responses to shear stress in atherosclerosis: a novel role for developmental genes. Nat Rev Cardiol 2020;17(1):52–63. doi: 10.1038/s41569-019-0239-5 31366922

[51] Wu MH. Endothelial focal adhesions and barrier function. J Physiol. 2005;569(Pt 2):359–66. doi: 10.1113/jphysiol.2005.096537 PMC1464245 16195317

[52] Dugina VB, Shagieva GS, Shakhov AS, Alieva IB. The cytoplasmic actins in the regulation of endothelial cell function. Int J Mol Sci 2021;22(15):7836. doi: 10.3390/ijms22157836 34360602 PMC8345992

[53] Tian T, Wan J, Song Q, Wei Z. Clustering single-cell RNA-seq data with a model-based deep learning approach. Nat Mach Intell 2019;1(4):191–8. doi: 10.1038/s42256-019-0037-0

[54] Li X, Wang K, Lyu Y, Pan H, Zhang J, Stambolian D, et al. Deep learning enables accurate clustering with batch effect removal in single-cell RNA-seq analysis. Nat Commun 2020;11(1):2338. doi: 10.1038/s41467-020-15851-3 32393754 PMC7214470

[55] Tian T, Zhang J, Lin X, Wei Z, Hakonarson H. Model-based deep embedding for constrained clustering analysis of single cell RNA-seq data. Nat Commun 2021;12(1):1873. doi: 10.1038/s41467-021-22008-3 33767149 PMC7994574

[56] Lopez R, Regier J, Cole MB, Jordan MI, Yosef N. Deep generative modeling for single-cell transcriptomics. Nat Methods 2018;15(12):1053–8. doi: 10.1038/s41592-018-0229-2 30504886 PMC6289068

[57] Lotfollahi M, Naghipourfar M, Theis FJ, Wolf FA. Conditional out-of-distribution generation for unpaired data using transfer VAE. Bioinformatics. 2020;36(Suppl. 2):i610–7. doi: 10.1093/bioinformatics/btaa800 33381839

[58] Sim X, Poncz M, Gadue P, French DL. Understanding platelet generation from megakaryocytes: implications for in vitro-derived platelets. Blood 2016;127(10):1227–33. doi: 10.1182/blood-2015-08-607929 26787738 PMC4786833

[59] Zeng H, Wang L, Li J, Luo S, Han Q, Su F, et al. Single-cell RNA-sequencing reveals distinct immune cell subsets and signaling pathways in IgA nephropathy. Cell Biosci 2021;11(1):203. doi: 10.1186/s13578-021-00706-1 34895340 PMC8665497

[60] Qi F, Zhang W, Huang J, Fu L, Zhao J. Single-cell RNA sequencing analysis of the immunometabolic rewiring and immunopathogenesis of coronavirus disease 2019. 2021;12:651656 10.3389/fimmu.2021.651656 PMC8079812 33936072

[61] Hwang B, Lee DS, Tamaki W, Sun Y, Ogorodnikov A, Hartoularos GC, et al. SCITO-seq: single-cell combinatorial indexed cytometry sequencing. Nat Methods 2021;18(8):903–11. doi: 10.1038/s41592-021-01222-3 34354295 PMC8643207

[62] Collin M, Bigley V. Human dendritic cell subsets: an update. Immunology 2018;154(1):3–20. doi: 10.1111/imm.12888 29313948 PMC5904714

