# Supplementary material for: scTrans: Sparse attention powers fast and accurate cell type annotation in single-cell RNA-seq data
Source: PLoS Comput Biol. 2025 Apr 4;21(4):e1012904. doi: 10.1371/journal.pcbi.1012904 (PMC11970913; doi:10.1371/journal.pcbi.1012904)
Supplement: S1 Fig — The details of encoder architecture. (DOCX) [file pcbi.1012904.s001.docx]

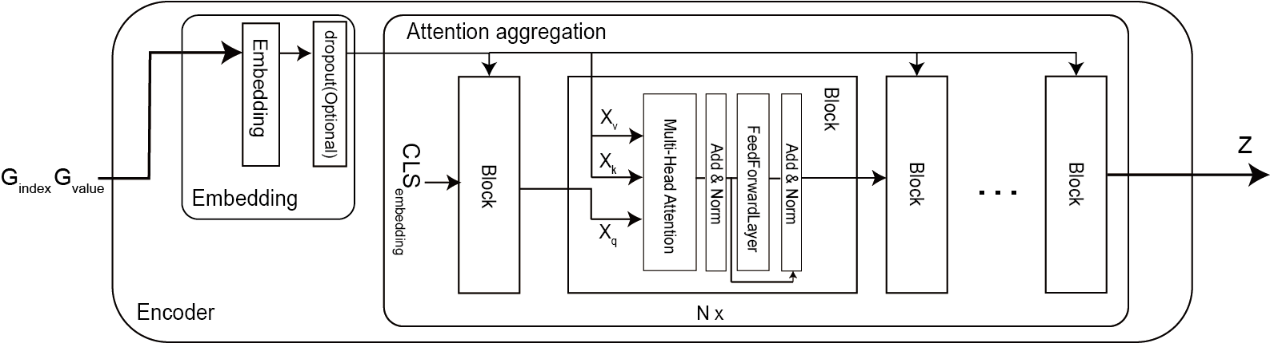


**S1 Fig. The details of encoder architecture.** Encoder of scTrans is a transformer-based encoder, consisting of embedding and attention aggregation module, attention aggregation consists of multiple attention blocks.
