# Supplementary material for: scTrans: Sparse attention powers fast and accurate cell type annotation in single-cell RNA-seq data
Source: PLoS Comput Biol. 2025 Apr 4;21(4):e1012904. doi: 10.1371/journal.pcbi.1012904 (PMC11970913; doi:10.1371/journal.pcbi.1012904)
Supplement: S2 Fig — Accuracy and f1-macro in large scale annotation task at different train rate. (DOCX) [file pcbi.1012904.s002.docx]

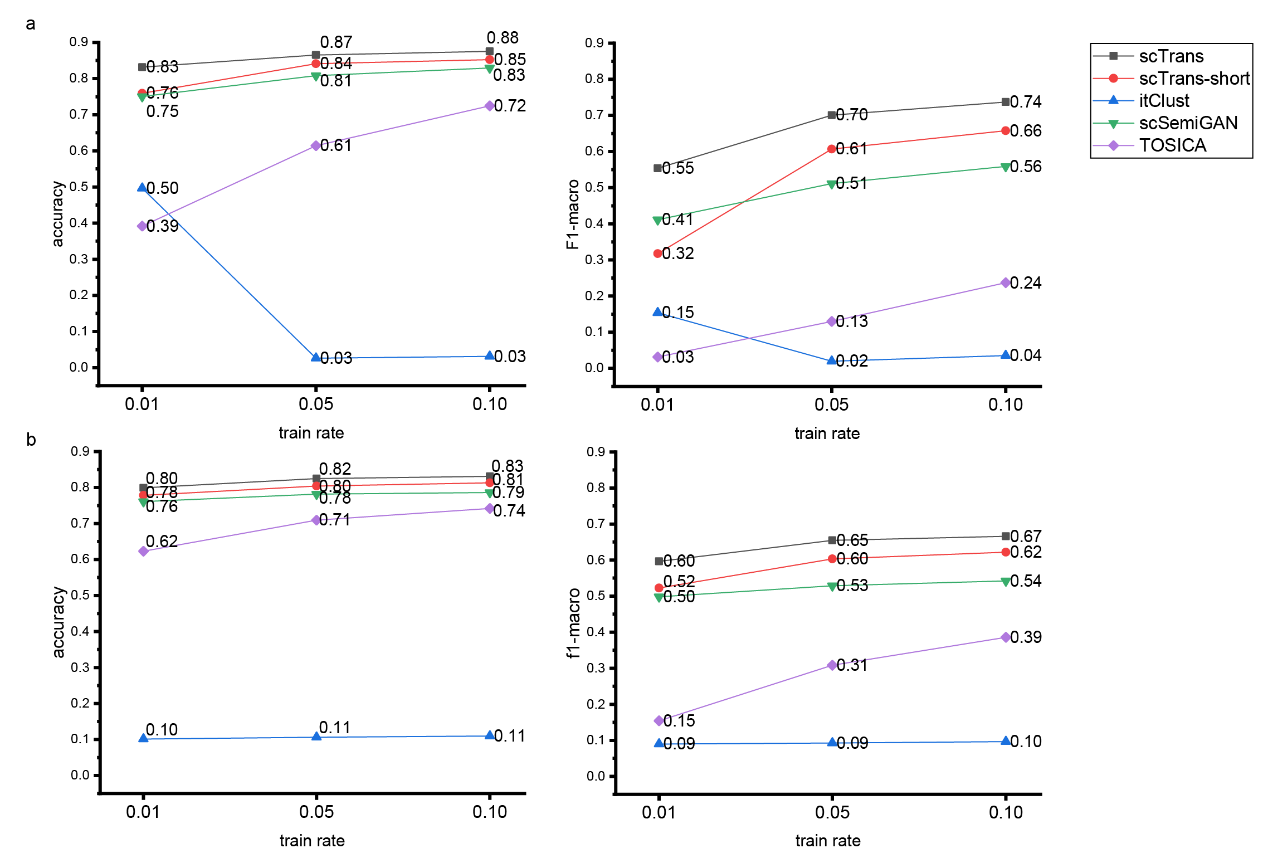


**S2 Fig. Accuracy and f1-macro in large scale annotation task at different train rate.** (a) Accuracy and f1-macro of each methods under 1%, 5% and 10% train rates on the PBMC160k dataset. (b) Accuracy and f1-macro of each method under 1%, 5% and 10% train rates on the scBloodNL dataset.
