# Supplementary material for: scTrans: Sparse attention powers fast and accurate cell type annotation in single-cell RNA-seq data
Source: PLoS Comput Biol. 2025 Apr 4;21(4):e1012904. doi: 10.1371/journal.pcbi.1012904 (PMC11970913; doi:10.1371/journal.pcbi.1012904)
Supplement: S3 Fig — The variation in annotation performance of scTrans across simulated datasets with different sequencing depths. (DOCX) [file pcbi.1012904.s003.docx]

**S3 Fig. The variation in annotation performance of scTrans across simulated datasets with different sequencing depths.** The X-axis represents the parameter "lib_loc" from splatter, which higher values indicate deeper sequencing depths. The Y-axis displays evaluation metrics, including accuracy and f1-macro.


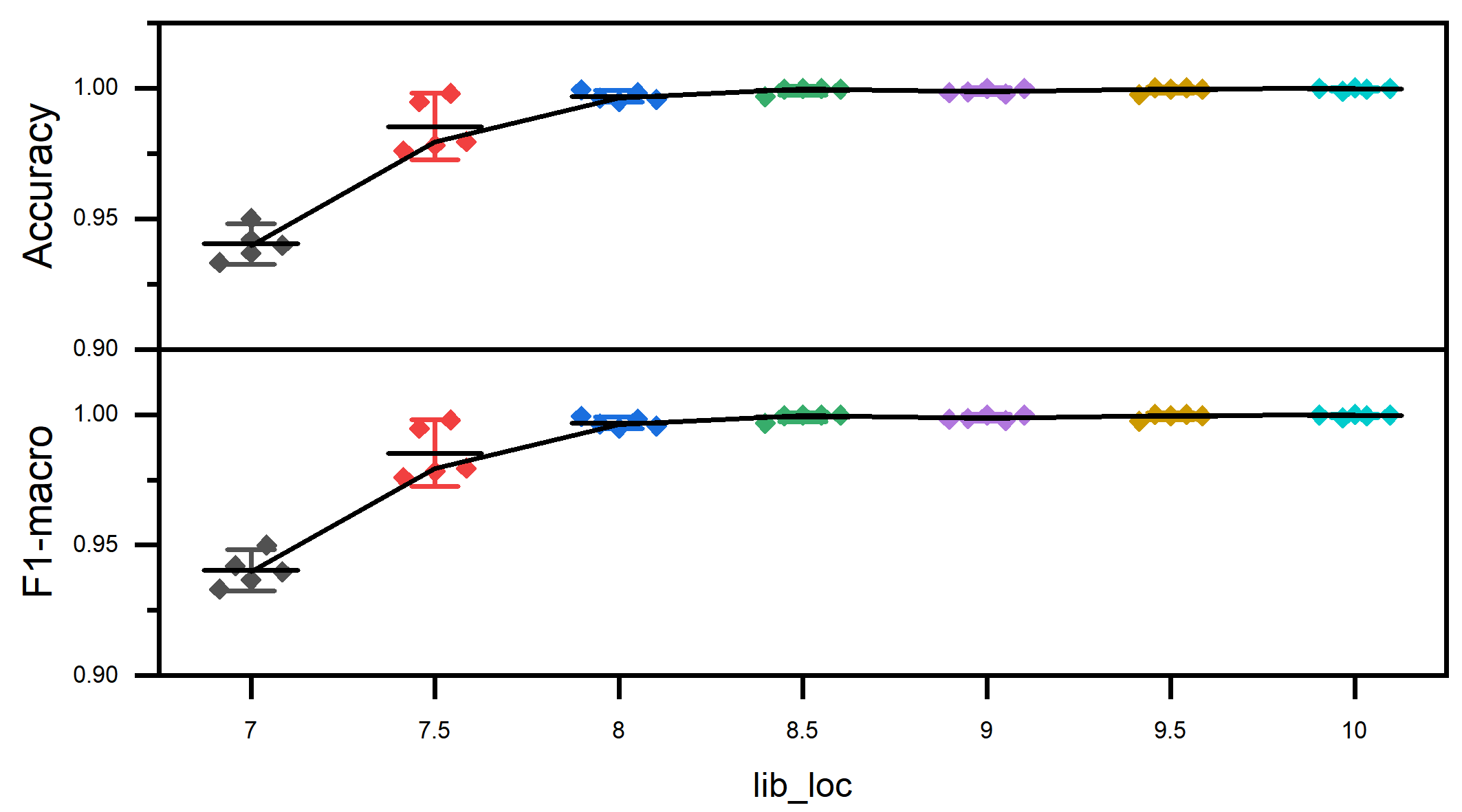


**Fig A.** The annotation results of scTrans for Model Dataset 1 under different sequencing depths. The Splatter simulation parameters are set as follows: cell type number is 5, the number of genes (nGene) is set to 10,000, the number of batch cells (batchCells) is 3,000, the differential expression probability (de.prob) is set to 0.2, and the library size (libloc) ranges from 7 to 10 to simulate different sequencing depths. All other parameters remain at their default values.


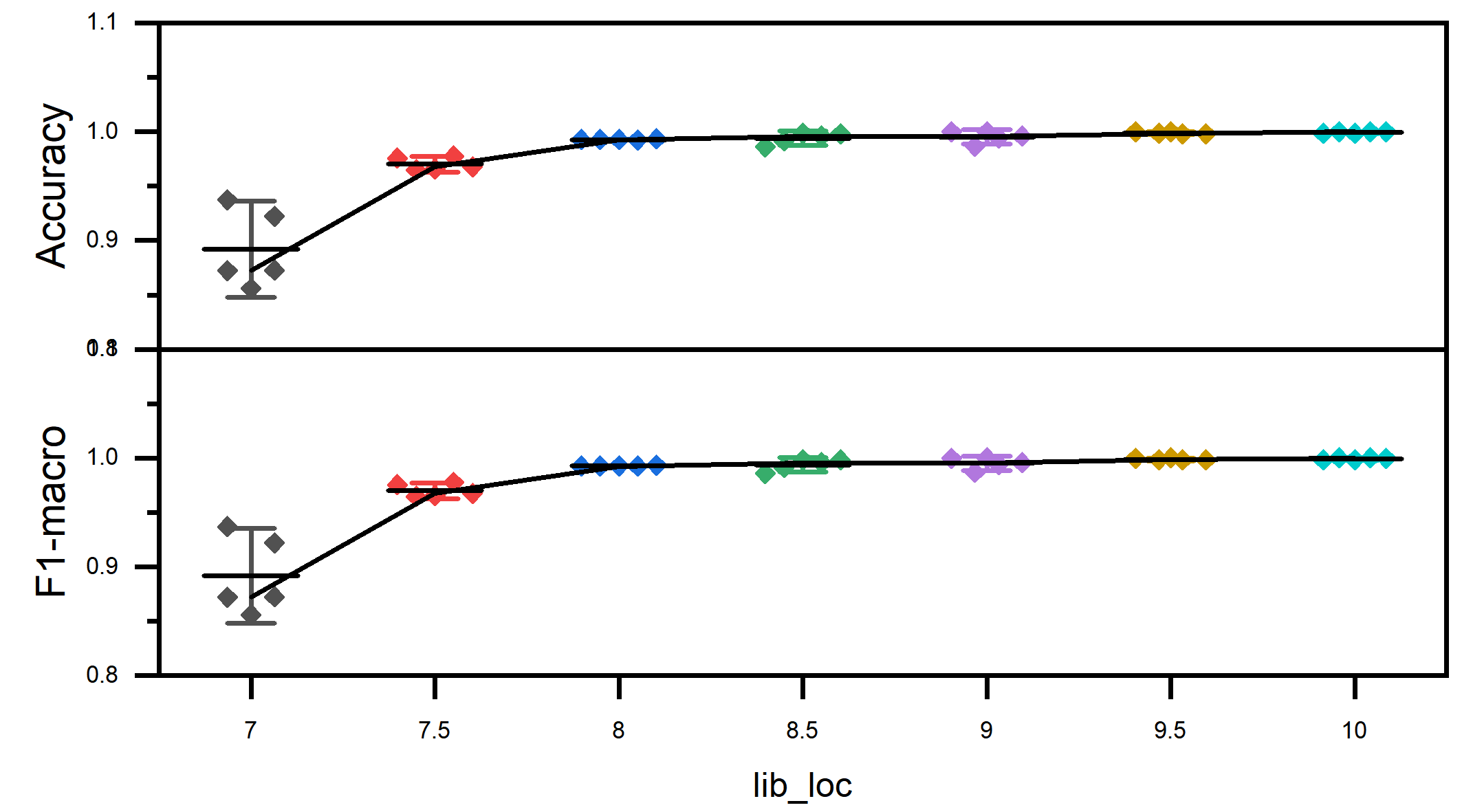


**Fig B.** The annotation results of scTrans for Model Dataset 1 under different sequencing depths. The Splatter simulation parameters are set as follows: cell type number is 10, the number of genes (nGene) is set to 10,000, the number of batch cells (batchCells) is 3,000, the differential expression probability (de.prob) is set to 0.2, and the library size (libloc) ranges from 7 to 10 to simulate different sequencing depths. All other parameters remain at their default values.


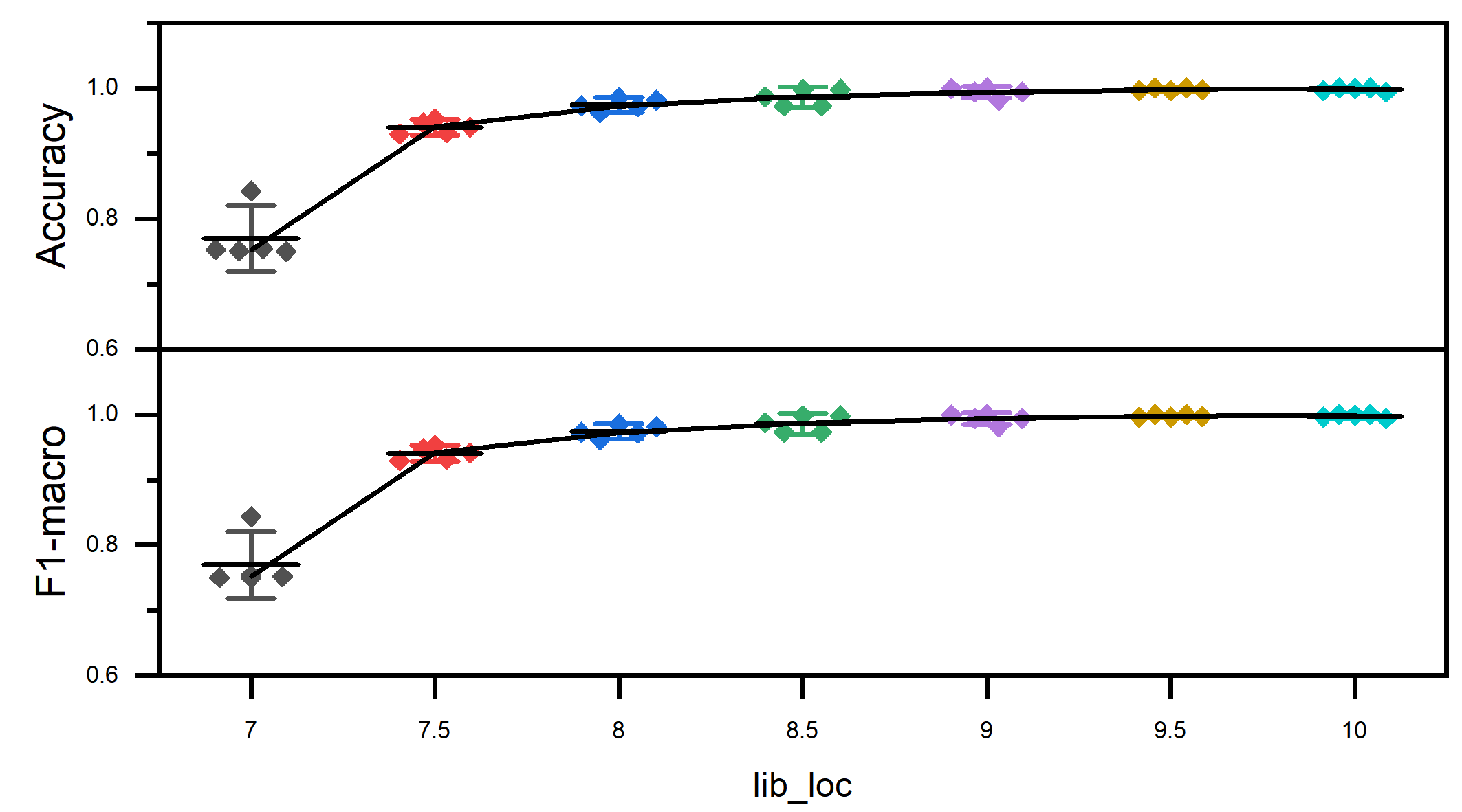


**Fig C.** The annotation results of scTrans for Model Dataset 1 under different sequencing depths. The Splatter simulation parameters are set as follows: cell type number is 15, the number of genes (nGene) is set to 10,000, the number of batch cells (batchCells) is 3,000, the differential expression probability (de.prob) is set to 0.2, and the library size (libloc) ranges from 7 to 10 to simulate different sequencing depths. All other parameters remain at their default values.
