# Supplementary material for: scTrans: Sparse attention powers fast and accurate cell type annotation in single-cell RNA-seq data
Source: PLoS Comput Biol. 2025 Apr 4;21(4):e1012904. doi: 10.1371/journal.pcbi.1012904 (PMC11970913; doi:10.1371/journal.pcbi.1012904)
Supplement: S4 Fig — Batch effects between two donors and cross technology annotation task results in PBMC45k datasets. (DOCX) [file pcbi.1012904.s004.docx]

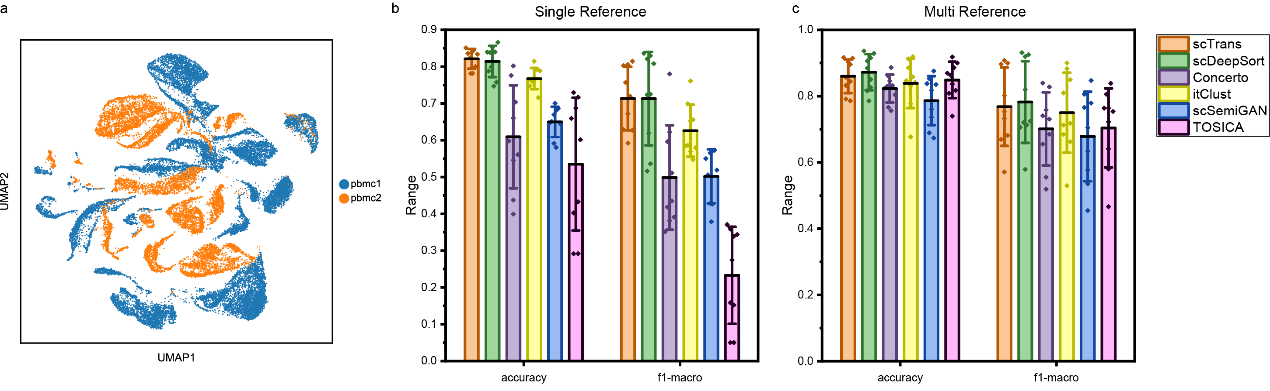


**S4 Fig. Batch effects between two donors and cross technology annotation task results in PBMC45k datasets.** (a) UMAP visualization results about 64-dimensional latent representation obtained from PCA of PBMC45k, indicating batch effects between different donors. (b-c) The accuracy and f1-macro results of bar plot on single reference and multi reference annotation task on PBMC45k dataset. Each point represents the average accuracy and f1 macro of a technical batch. All error bars are based on mean and 95% confidence.
