# Supplementary material for: scTrans: Sparse attention powers fast and accurate cell type annotation in single-cell RNA-seq data
Source: PLoS Comput Biol. 2025 Apr 4;21(4):e1012904. doi: 10.1371/journal.pcbi.1012904 (PMC11970913; doi:10.1371/journal.pcbi.1012904)
Supplement: S5 Fig — F1-macro of cross batch annotation results in mouse brain and mouse pancreas datasets. (DOCX) [file pcbi.1012904.s005.docx]

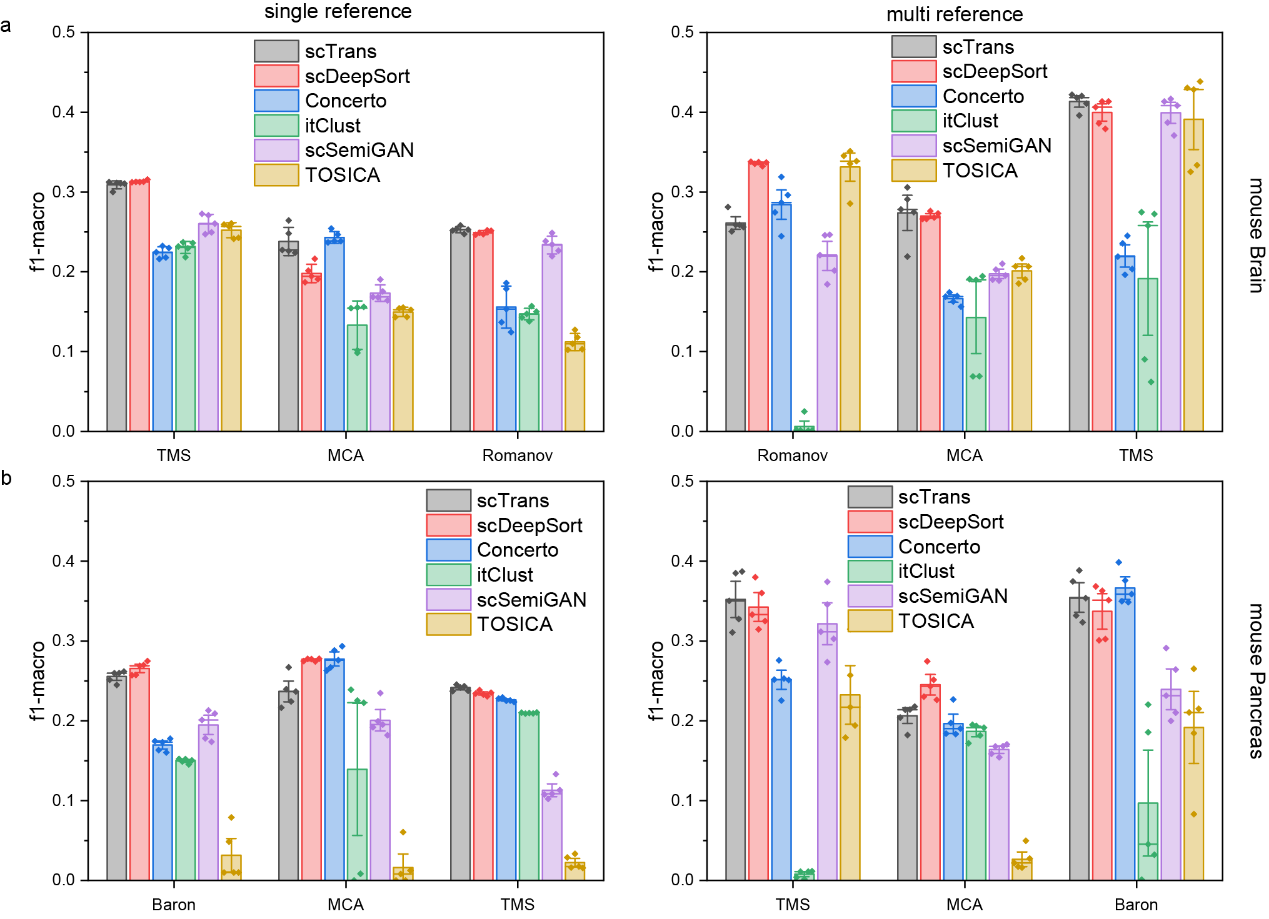


**S5 Fig. F1-macro of cross batch annotation results in mouse brain and mouse pancreas datasets.** (a) F1-macro results for cross batch annotation on mouse brains. On the left is single reference, and on the right is multi reference. (b) F1-macro results for cross batch annotation on mouse pancreas. On the left is single reference, and on the right is multi reference. All error bars are based on mean and 95% confidence
