# Supplementary material for: scTrans: Sparse attention powers fast and accurate cell type annotation in single-cell RNA-seq data
Source: PLoS Comput Biol. 2025 Apr 4;21(4):e1012904. doi: 10.1371/journal.pcbi.1012904 (PMC11970913; doi:10.1371/journal.pcbi.1012904)
Supplement: S6 Fig — Sankey diagram of annotation results in cross datasets multi reference annotation task. (DOCX) [file pcbi.1012904.s006.docx]

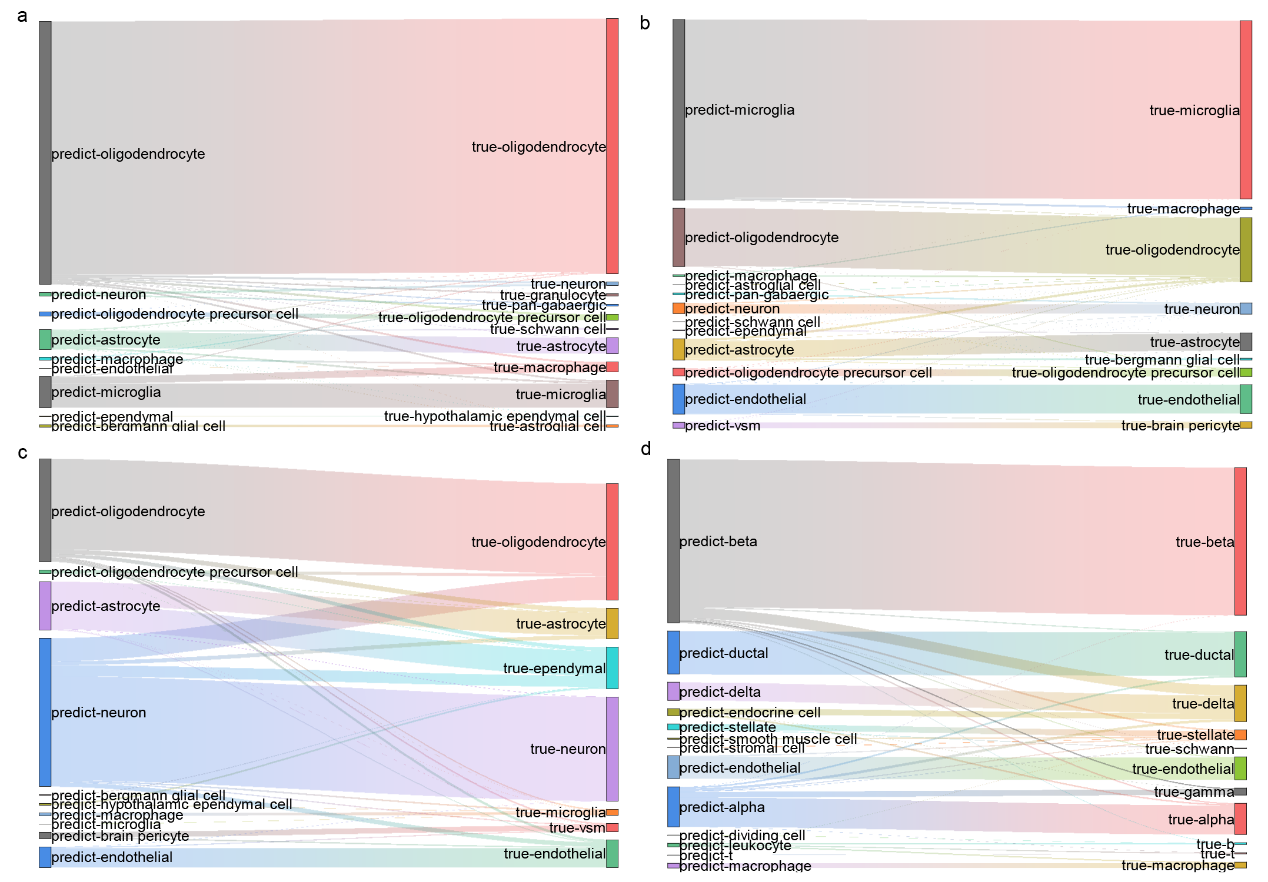


**S6 Fig. Sankey diagram of annotation results in cross datasets multi reference annotation task. On the left is the scTrans prediction result, and on the right is the ground truth.** (a) Results of multi reference annotation in the MCA Brain dataset. (b) Results of multi reference annotation in the TMS Brain dataset. (c) Results of multi reference annotation in the Romanov dataset. (d) Results of multi reference annotation in the Baron dataset.
