# Supplementary material for: scTrans: Sparse attention powers fast and accurate cell type annotation in single-cell RNA-seq data
Source: PLoS Comput Biol. 2025 Apr 4;21(4):e1012904. doi: 10.1371/journal.pcbi.1012904 (PMC11970913; doi:10.1371/journal.pcbi.1012904)
Supplement: S7 Fig — UMAP visualization results of Cell types and Tnc gene expression in MCA Brain dataset. (DOCX) [file pcbi.1012904.s007.docx]

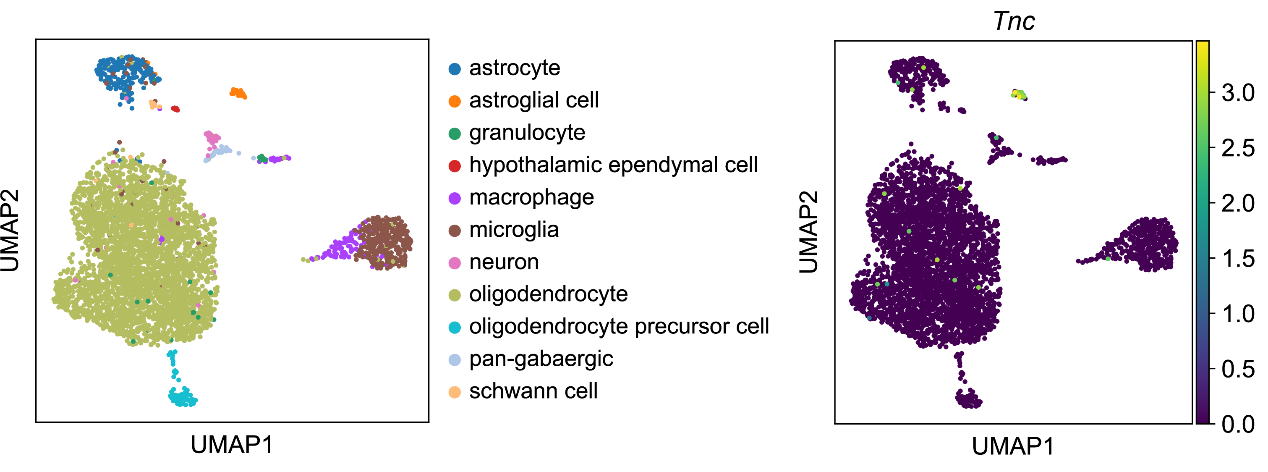


**S7 Fig. UMAP visualization results of Cell types and TNC gene expression in MCA Brain dataset.** Tnc is a marker gene for Bergmann glial cells, and its distribution is like astroglial cell in MCA Brain datasets.
