# Supplementary material for: scTrans: Sparse attention powers fast and accurate cell type annotation in single-cell RNA-seq data
Source: PLoS Comput Biol. 2025 Apr 4;21(4):e1012904. doi: 10.1371/journal.pcbi.1012904 (PMC11970913; doi:10.1371/journal.pcbi.1012904)
Supplement: S8 Fig — Critical gene analysis results. (A) Critical genes heatmap of MCA Brain, TMS Brain, Romanov, MCA Pancreas and TMS Pancreas datasets. (B) KEGG analysis results of endothelial cells in MCA Pancreas and TMS Pancreas datasets. (C) Venn diagrams illustrating the enrichment analysis results of endothelial cells across three datasets. (DOCX) [file pcbi.1012904.s008.docx]

**S8 Fig. Critical gene analysis results. Fig A. Critical genes heatmap of MCA Brain, TMS Brain, Romanov, MCA Pancreas and TMS Pancreas datasets. Fig B. KEGG analysis results of endothelial cells in Romanov and TMS Pancreas datasets. Fig C. Venn diagrams illustrating the enrichment analysis results of endothelial cells across three datasets.**


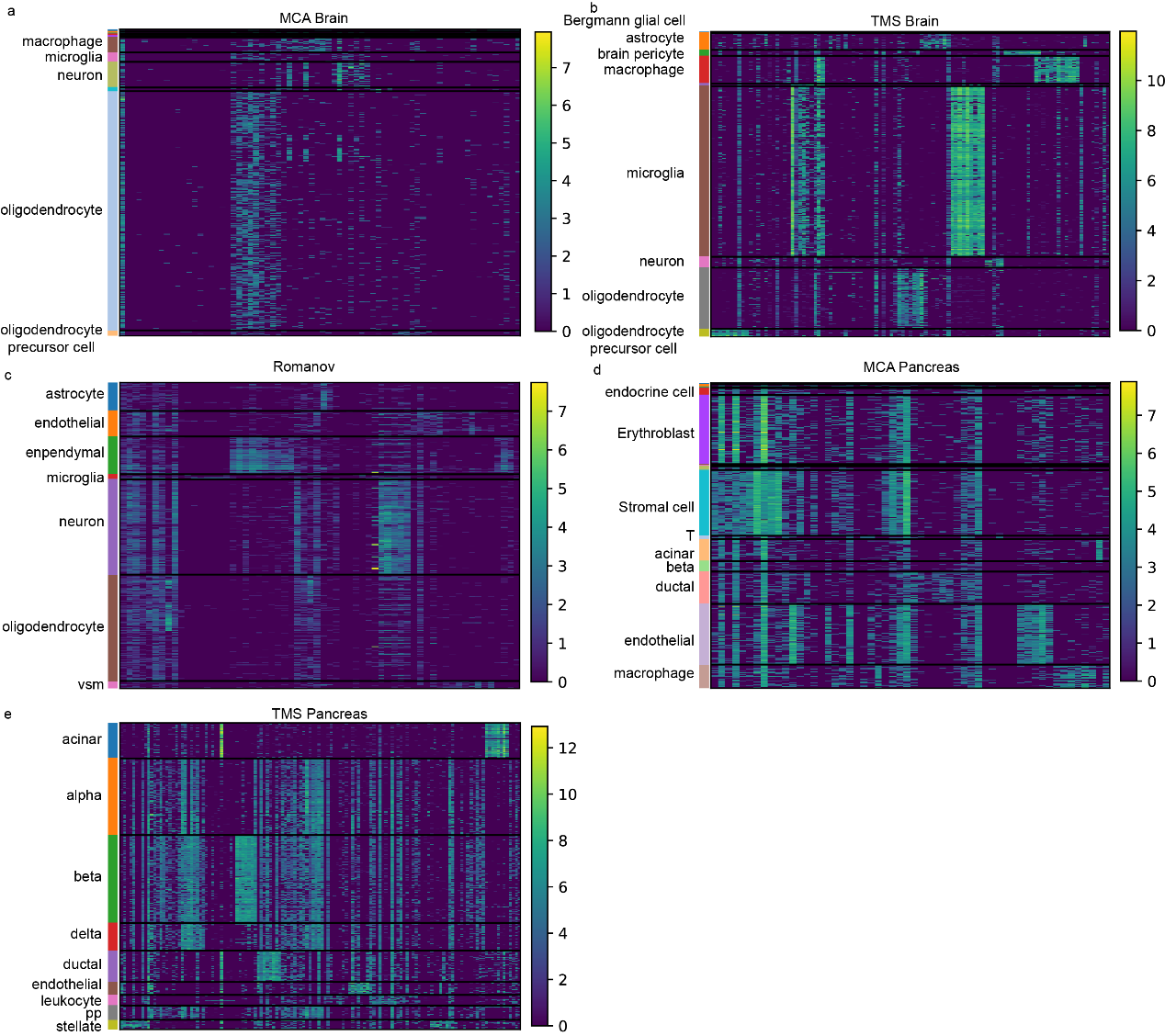


**Fig A. Critical genes heatmap of MCA Brain, TMS Brain, Romanov, MCA Pancreas and TMS Pancreas datasets.** a-e The top 10 highest attention weights critical genes selected based on each predict results, and heatmap visualization was performed on these genes.


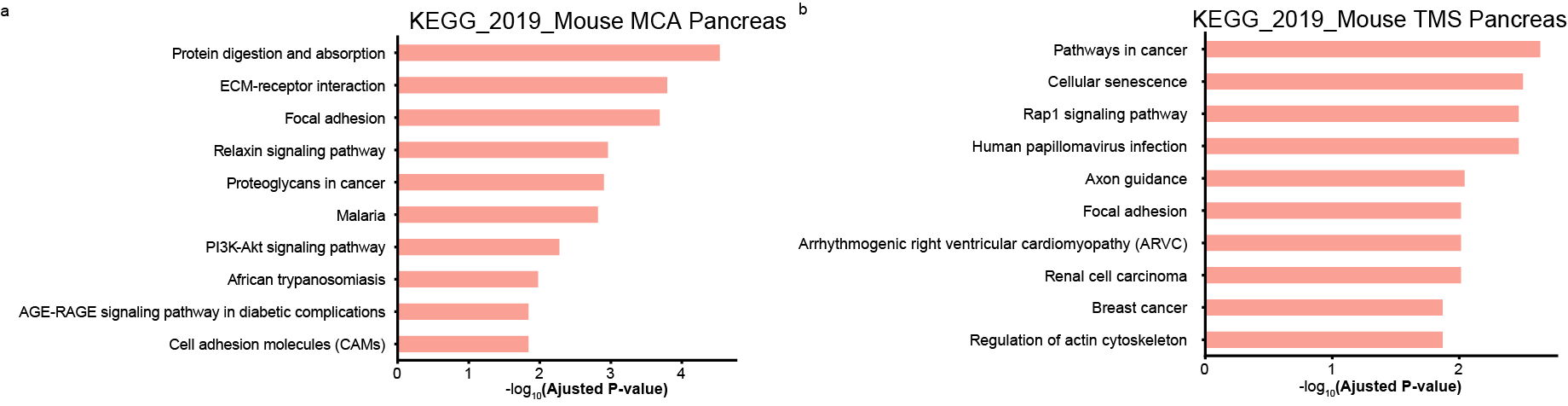


**Fig B. KEGG analysis results of endothelial cells in Romanov and TMS Pancreas datasets.** (a-b) Top 10 KEGG analysis results of endothelial cells in Romanov and TMS Pancreas datasets.


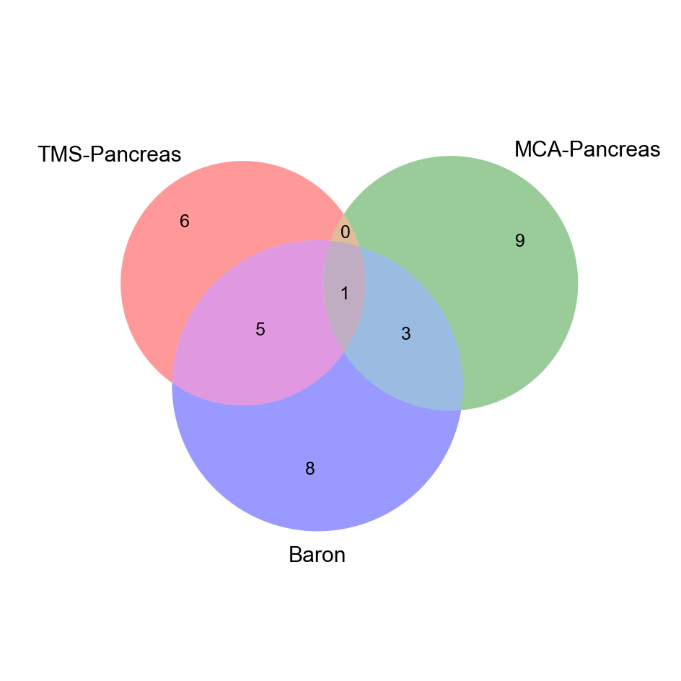


**Fig C. Venn diagrams illustrating the enrichment analysis results of endothelial cells across three datasets.**
