# Supplementary material for: scTrans: Sparse attention powers fast and accurate cell type annotation in single-cell RNA-seq data
Source: PLoS Comput Biol. 2025 Apr 4;21(4):e1012904. doi: 10.1371/journal.pcbi.1012904 (PMC11970913; doi:10.1371/journal.pcbi.1012904)
Supplement: S9 Fig — This figure presents the stacked violin plots of the top 10 critical genes for six cell types in the Baron dataset. (A–C) Respectively, the stacked violin plots for alpha, beta, delta. (DOCX) [file pcbi.1012904.s009.docx]

**S9 Fig.** **This figure presents the stacked violin plots of the top 10 critical genes for six cell types in the Baron dataset. Fig A-C.** **Respectively, the stacked violin plots for alpha, beta, delta.**


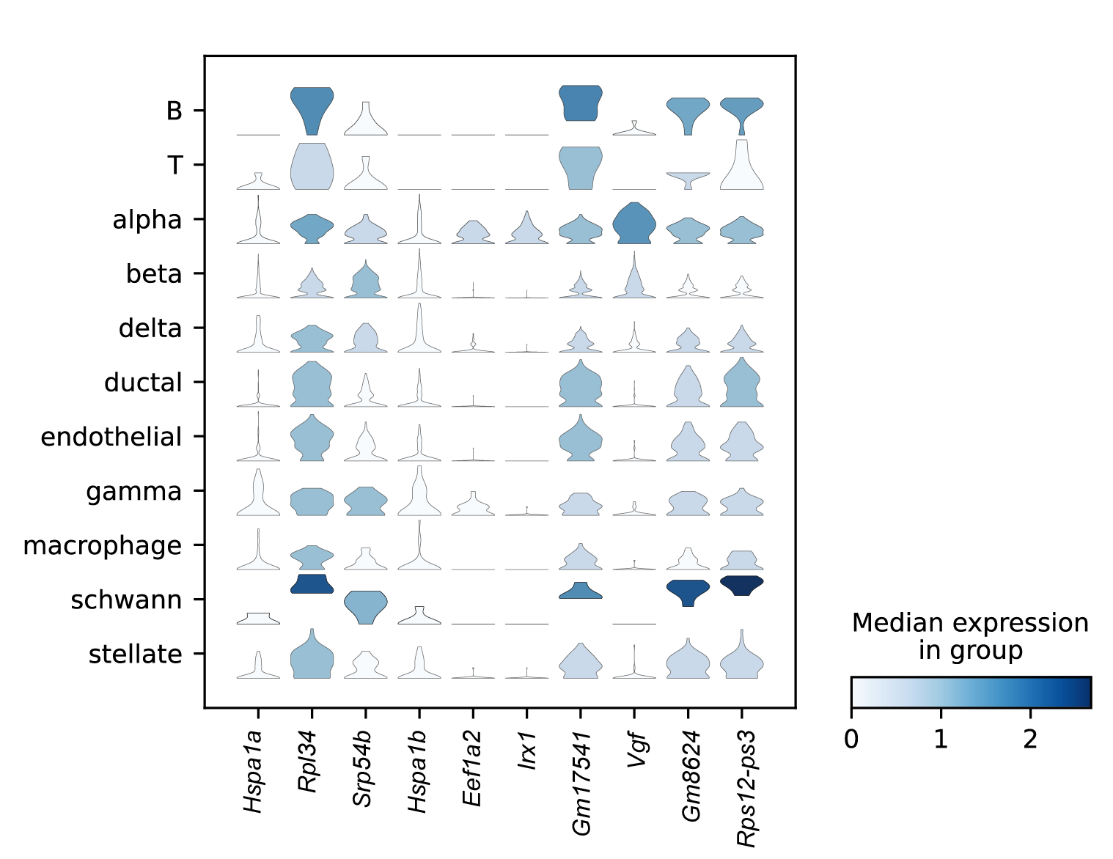


**Fig A. The figure displays the stacked violin plots of gene expression for the top 10 critical genes of alpha cells in the Baron dataset.**


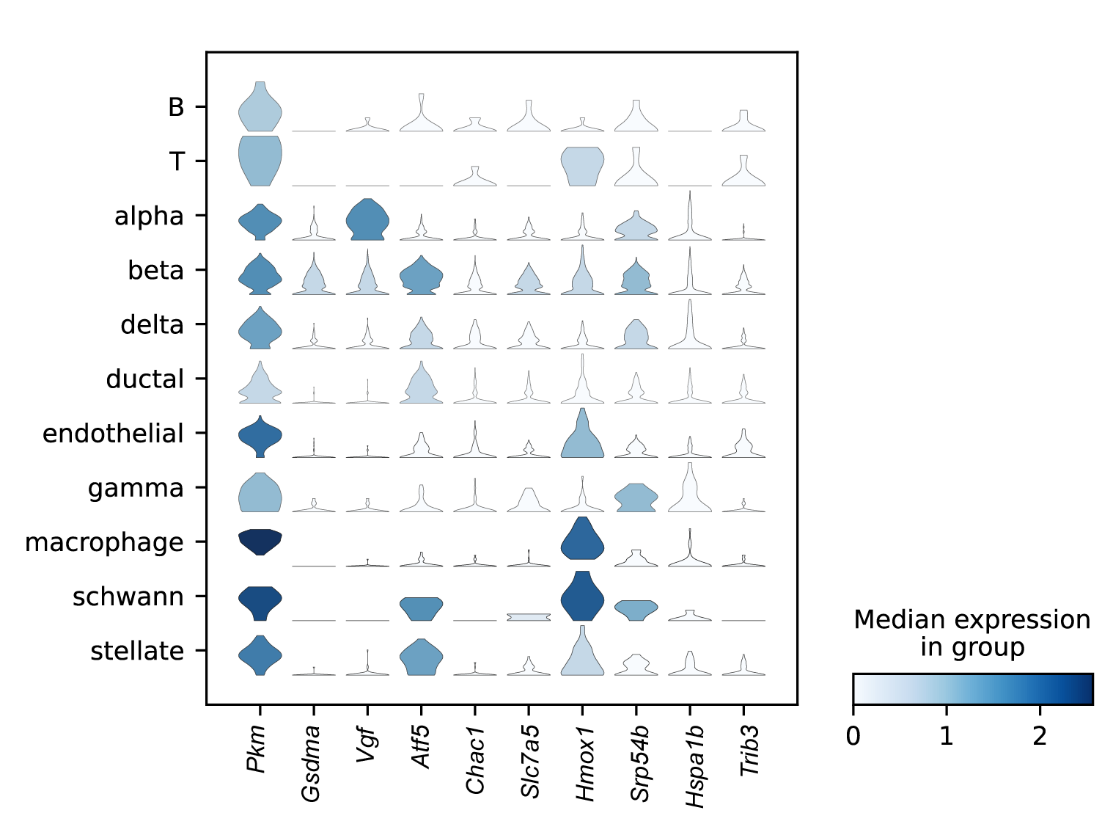


**Fig B. The figure displays the stacked violin plots of gene expression for the top 10 critical genes of beta cells in the Baron dataset.**


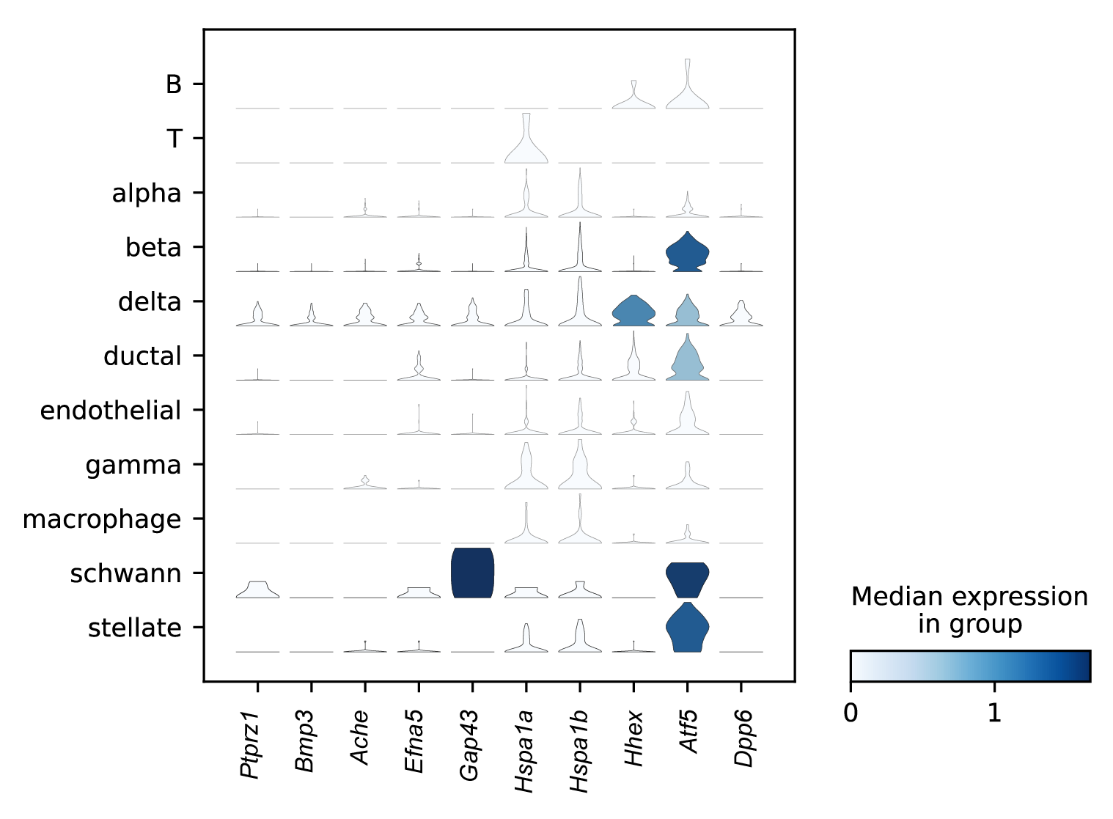


**Fig C. The figure displays the stacked violin plots of gene expression for the top 10 critical genes of delta cells in the Baron dataset.**
