# Supplementary material for: scTrans: Sparse attention powers fast and accurate cell type annotation in single-cell RNA-seq data
Source: PLoS Comput Biol. 2025 Apr 4;21(4):e1012904. doi: 10.1371/journal.pcbi.1012904 (PMC11970913; doi:10.1371/journal.pcbi.1012904)

**S10 Fig.** **This figure presents a comparison of marker weight ranking scores extracted by scTrans versus those generated randomly through randomization testing, highlighting the significance of the weights extracted by scTrans.**


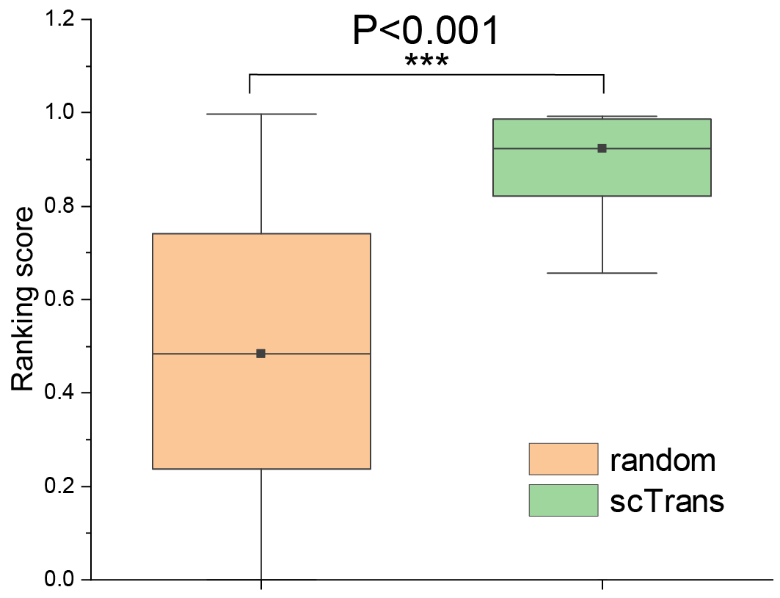

Supplement: S10 Fig — This figure presents a comparison of marker weight ranking scores extracted by scTrans versus those generated randomly through randomization testing. (DOCX) [file pcbi.1012904.s010.docx]
