# Supplementary material for: scTrans: Sparse attention powers fast and accurate cell type annotation in single-cell RNA-seq data
Source: PLoS Comput Biol. 2025 Apr 4;21(4):e1012904. doi: 10.1371/journal.pcbi.1012904 (PMC11970913; doi:10.1371/journal.pcbi.1012904)
Supplement: S11 Fig — Reactome and Go enrichment analysis results of top 100 critical genes of endothelial cells in Baron dataset. (DOCX) [file pcbi.1012904.s011.docx]

**S11 Fig.** **Reactome and Go enrichment analysis results of top 100 critical genes of endothelial cells in Baron dataset. Fig A. The results of Reactome enrichment analysis results. Fig B-D.** **The results of GO enrichment analysis of biological process, cellular component and molecular function are presented respectively.**


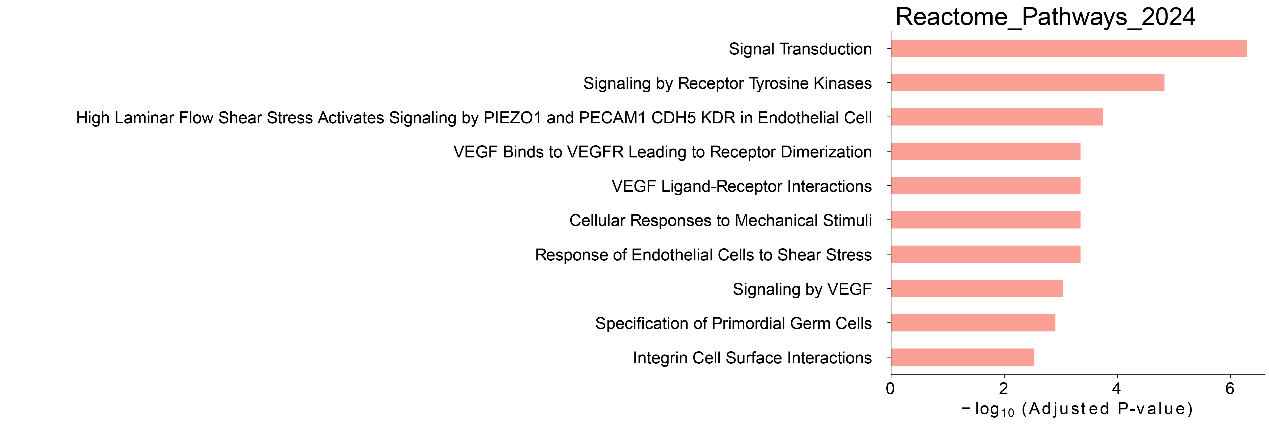


**Fig A. Reactome enrichment analysis results.**


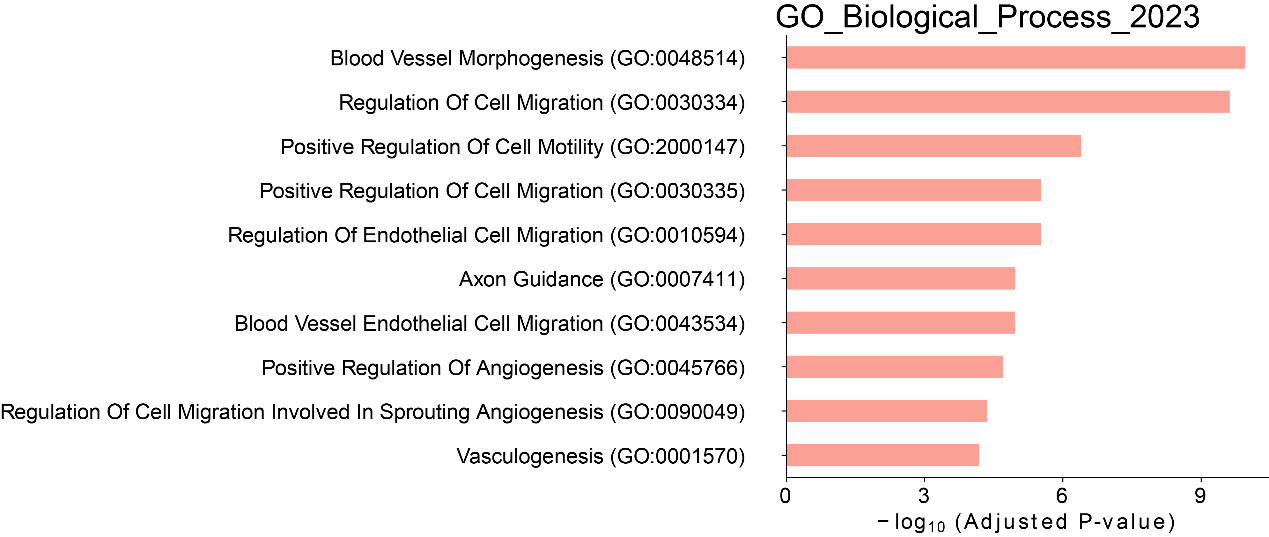


**Fig B. GO enrichment analysis results of biological process.**


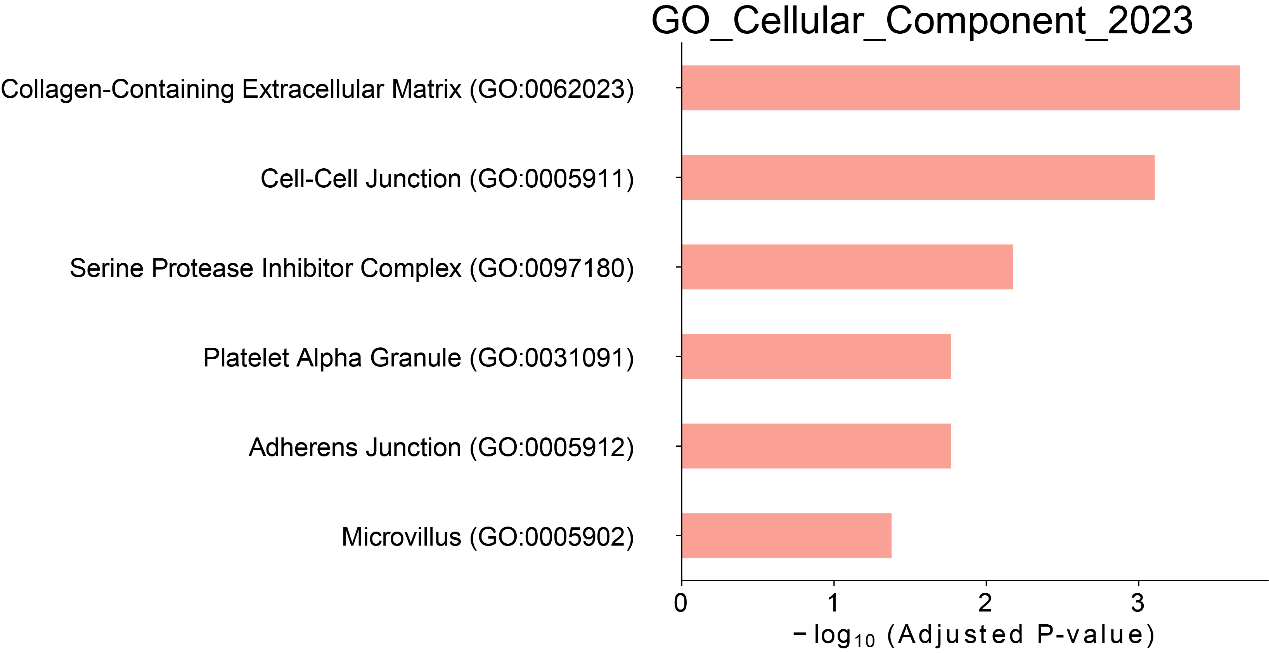


**Fig C. GO enrichment analysis results of cellular process.**


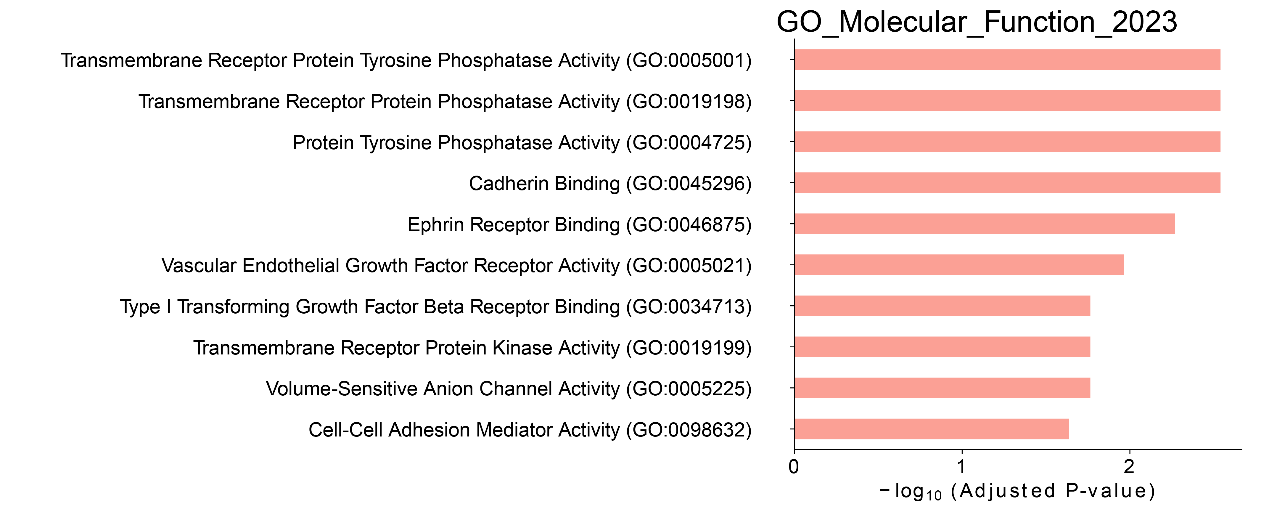


**Fig D. GO enrichment analysis results of molecular function.**
