# Supplementary material for: scTrans: Sparse attention powers fast and accurate cell type annotation in single-cell RNA-seq data
Source: PLoS Comput Biol. 2025 Apr 4;21(4):e1012904. doi: 10.1371/journal.pcbi.1012904 (PMC11970913; doi:10.1371/journal.pcbi.1012904)
Supplement: S12 Fig — UMAP visualization of latent representations generated by scTrans, scSemiGAN and scDeepSort for mouse brain and mouse pancreas datasets. (A–F) UMAP visualization about latent representation in MCA Pancreas, MCA Brain, TMS Brain, TM Pancreas, Baron and Romanov datasets. (DOCX) [file pcbi.1012904.s012.docx]

**S12 Fig. UMAP visualization of latent representations generated by scTrans, scSemiGAN and scDeepSort for mouse brain and mouse pancreas datasets. Fig A-F. UMAP visualization about latent representation in MCA Pancreas, MCA Brain, TMS Brain, TM Pancreas, Baron and Romanov datasets.** All methods trained on reference datasets in multi reference task, then generate the latent representation of query datasets. Visualized clustering results, cell type prediction results, and truly cell type.


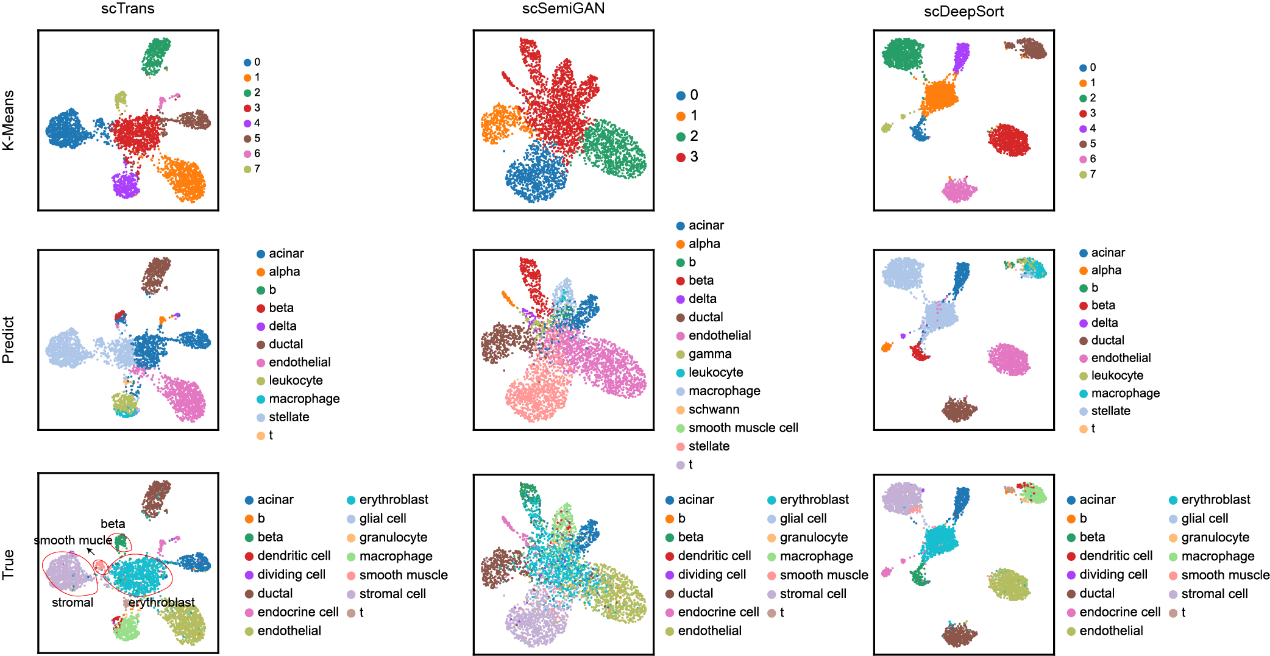


**Fig A. UMAP visualization about latent representation in MCA Pancreas datasets.**


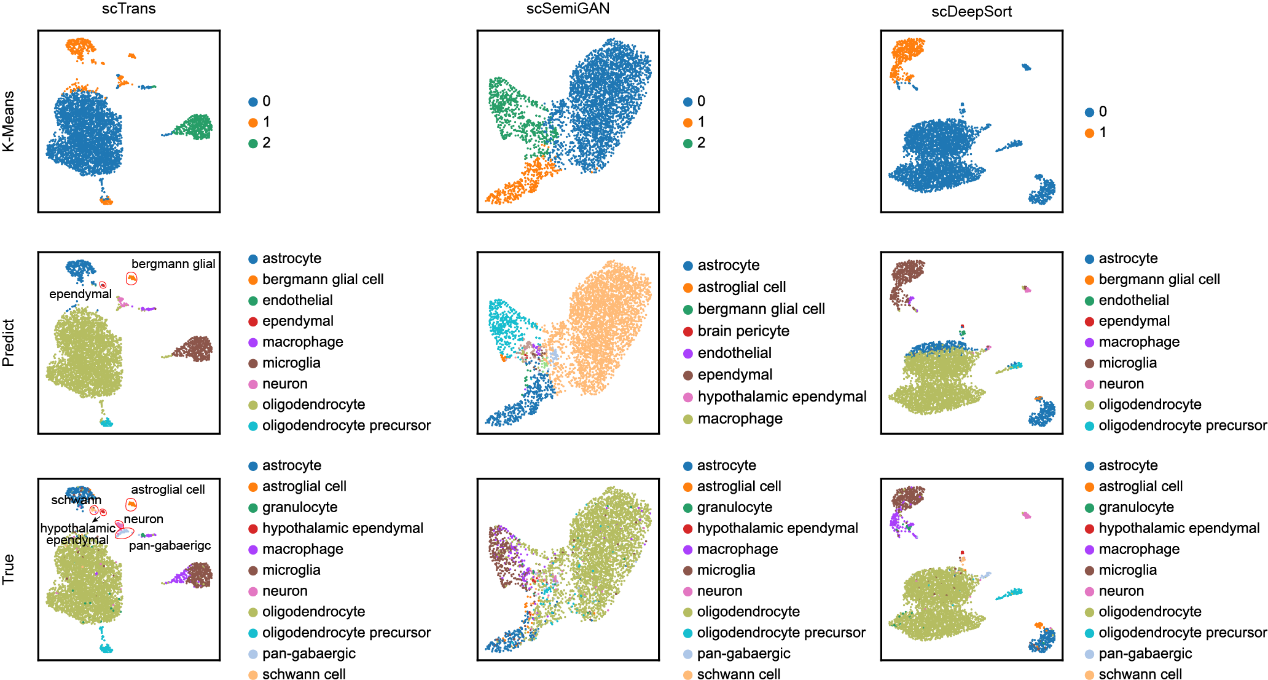


**Fig B. UMAP visualization about latent representation in MCA Brain datasets.**


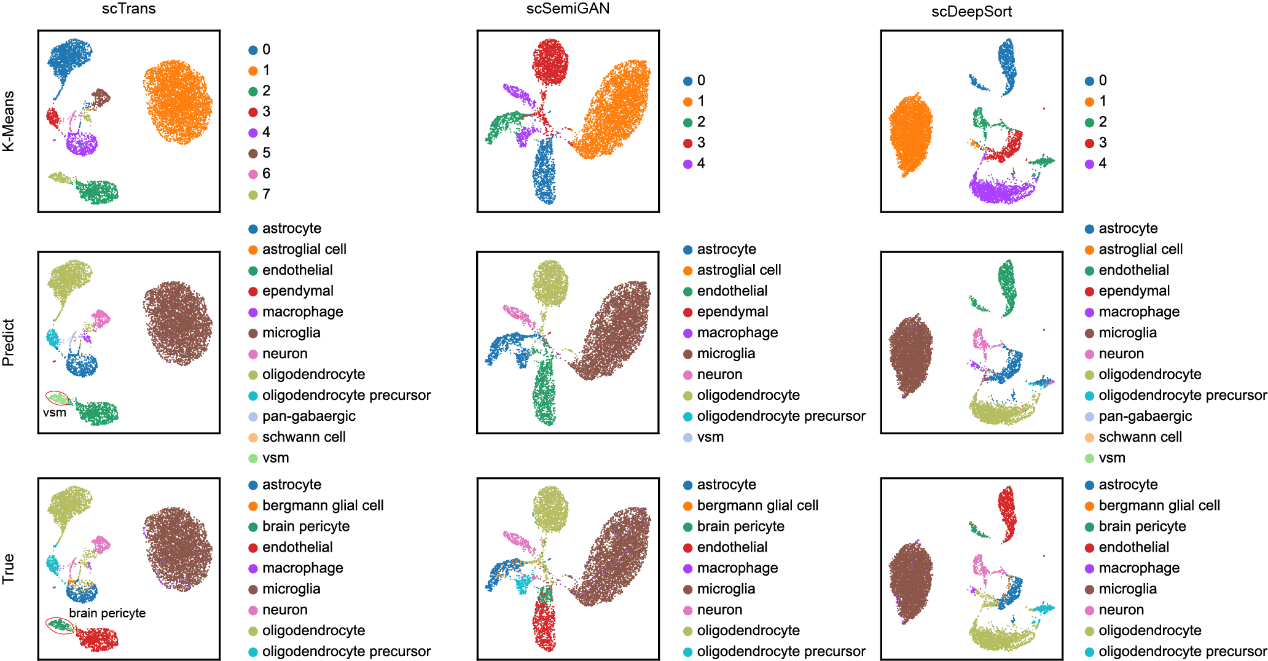


**Fig C. UMAP visualization about latent representation in TMS Brain datasets.**


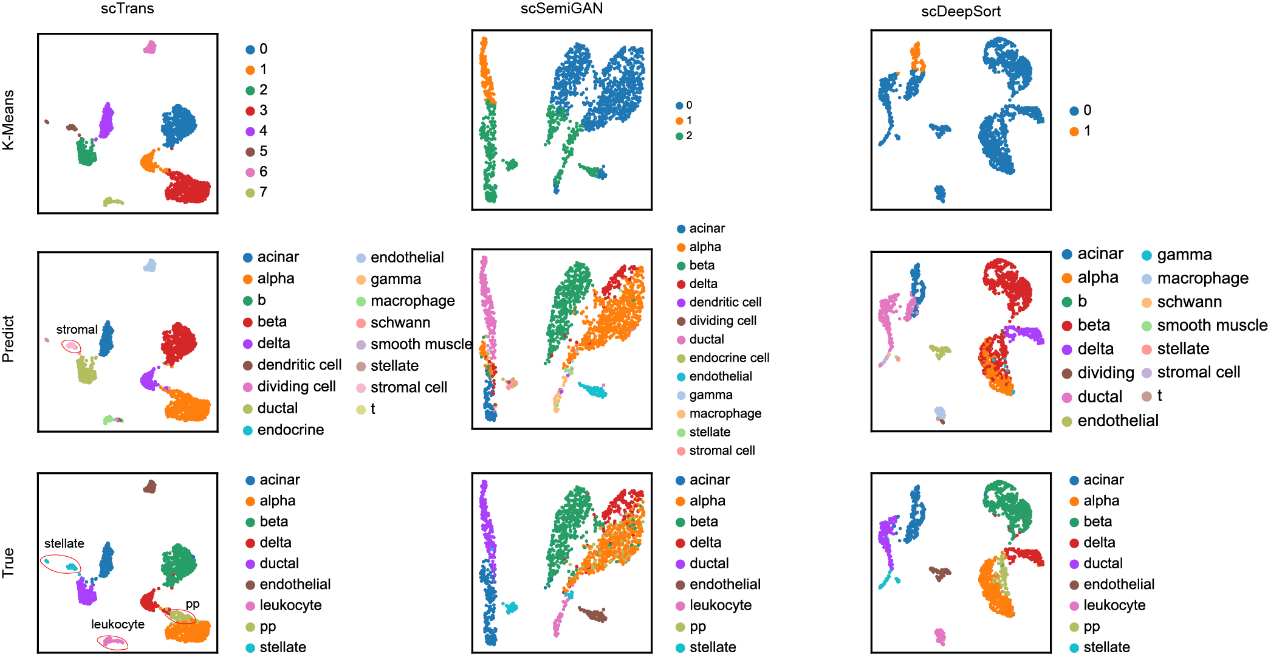


**Fig D. UMAP visualization about latent representation in TMS Pancreas datasets.**


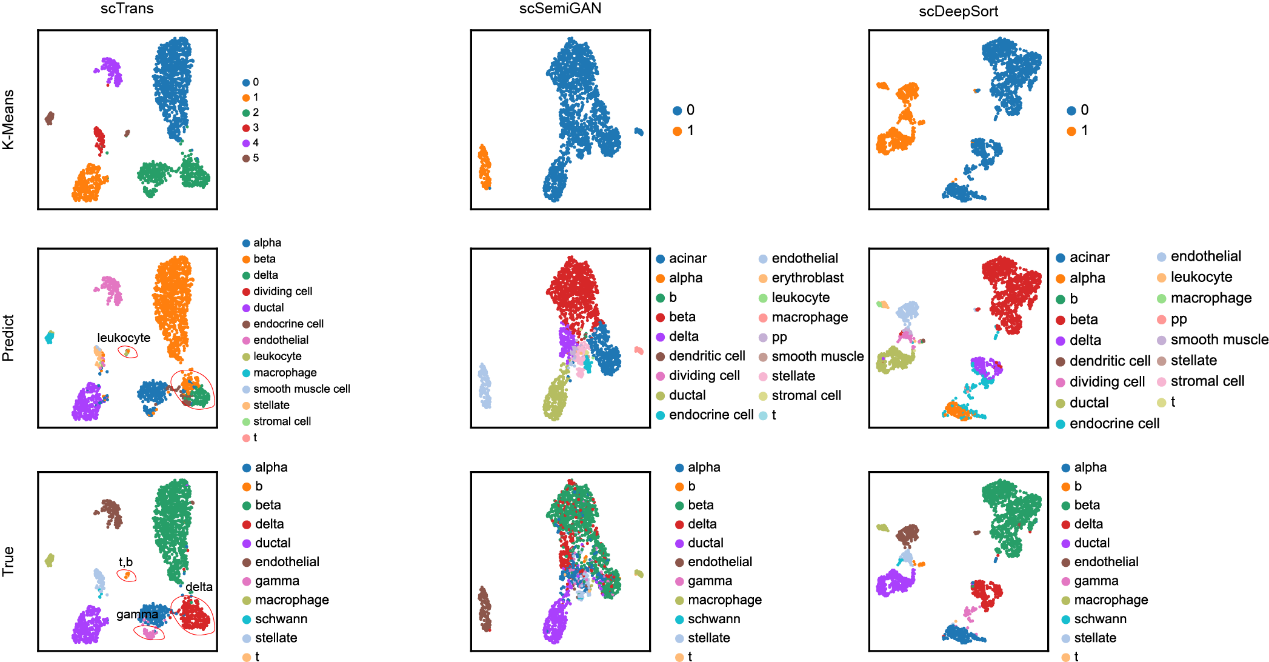


**Fig E. UMAP visualization about latent representation in Baron datasets.**


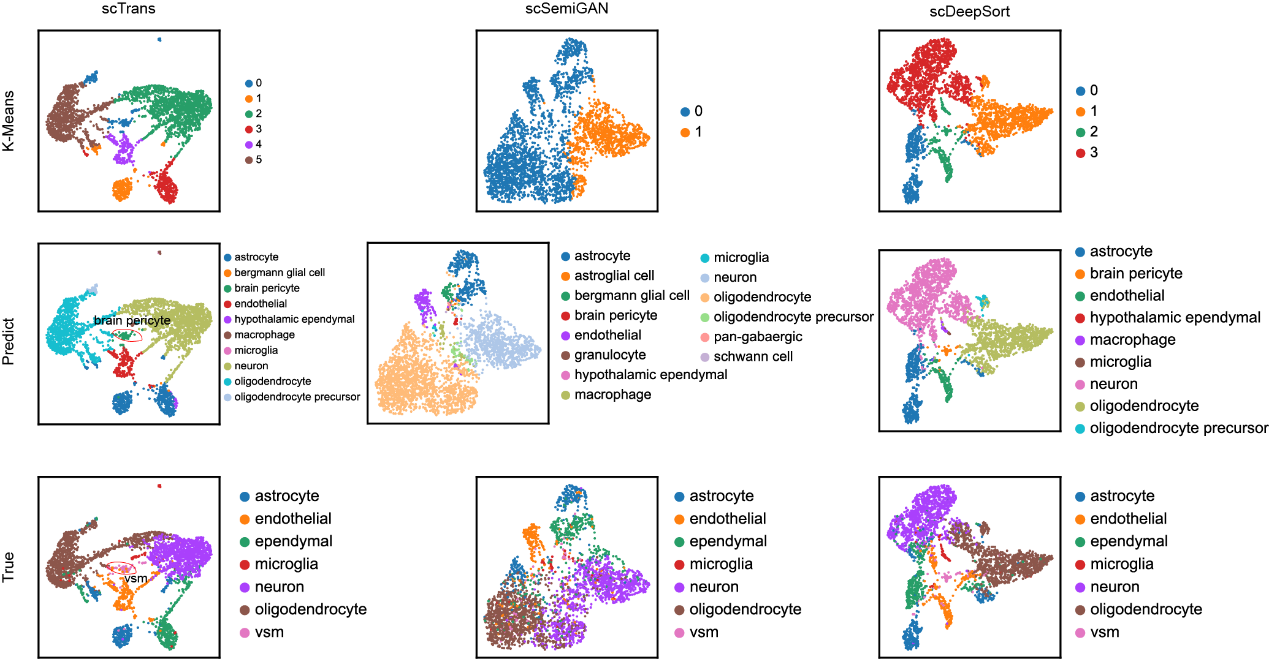


**Fig F. UMAP visualization about latent representation in Romanov datasets.**
