# Supplementary material for: scTrans: Sparse attention powers fast and accurate cell type annotation in single-cell RNA-seq data
Source: PLoS Comput Biol. 2025 Apr 4;21(4):e1012904. doi: 10.1371/journal.pcbi.1012904 (PMC11970913; doi:10.1371/journal.pcbi.1012904)
Supplement: S13 Fig — ASW and NMI of clustering results in mouse Brain and mouse Pancreas datasets. (DOCX) [file pcbi.1012904.s013.docx]

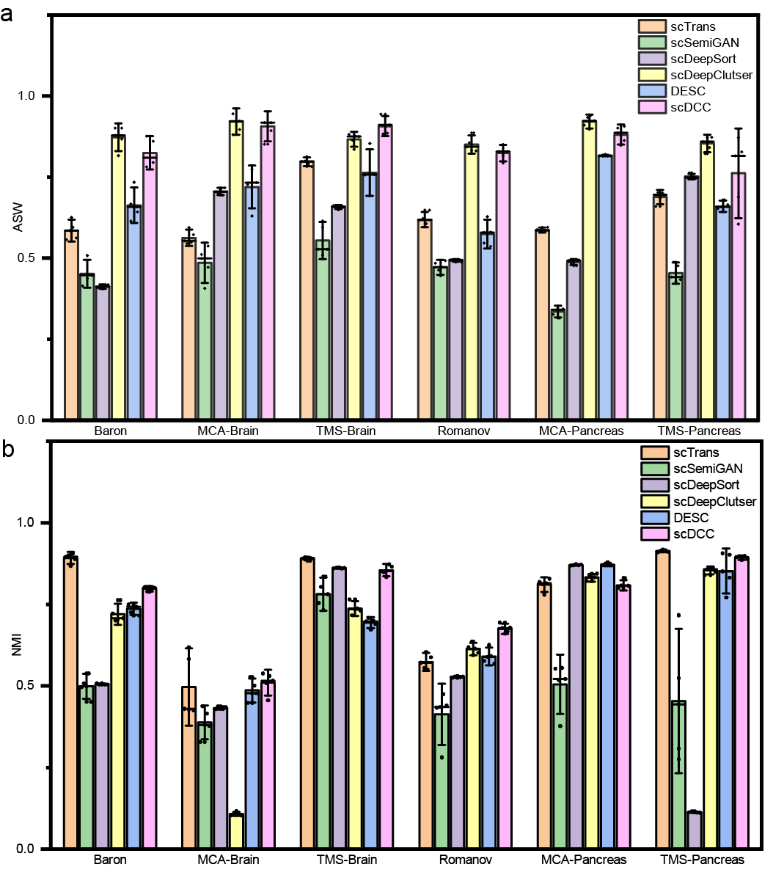


**S13 Fig. ASW and NMI of clustering results in mouse Brain and mouse Pancreas datasets.** (a-b) Two evaluation metrics that measure clustering results: ASW and NMI. Comparing scTrans with two cell type annotation methods scSemiGAN and scDeepSort, two unsupervised clustering methods scDeepCluster and DESC, and one semi supervised clustering method scDCC. All error bars are based on mean and 95% confidence.
