# Supplementary material for: scTrans: Sparse attention powers fast and accurate cell type annotation in single-cell RNA-seq data
Source: PLoS Comput Biol. 2025 Apr 4;21(4):e1012904. doi: 10.1371/journal.pcbi.1012904 (PMC11970913; doi:10.1371/journal.pcbi.1012904)
Supplement: S14 Fig — UMAP visualization of latent representations generated by scDeepCluster, DESC and scDCC for mouse brain and mouse pancreas datasets. (A–C) UMAP visualization of latent representation in mouse Brain and mouse Pancreas datasets generated by scDeepCluster, DESC and scDCC. (DOCX) [file pcbi.1012904.s014.docx]

**S14 Fig. UMAP visualization of latent representations generated by scDeepCluster, DESC and scDCC for mouse brain and mouse pancreas datasets. Fig A-C. UMAP visualization of latent representation in mouse Brain and mouse Pancreas datasets generated by scDeepCluster, DESC and scDCC.**


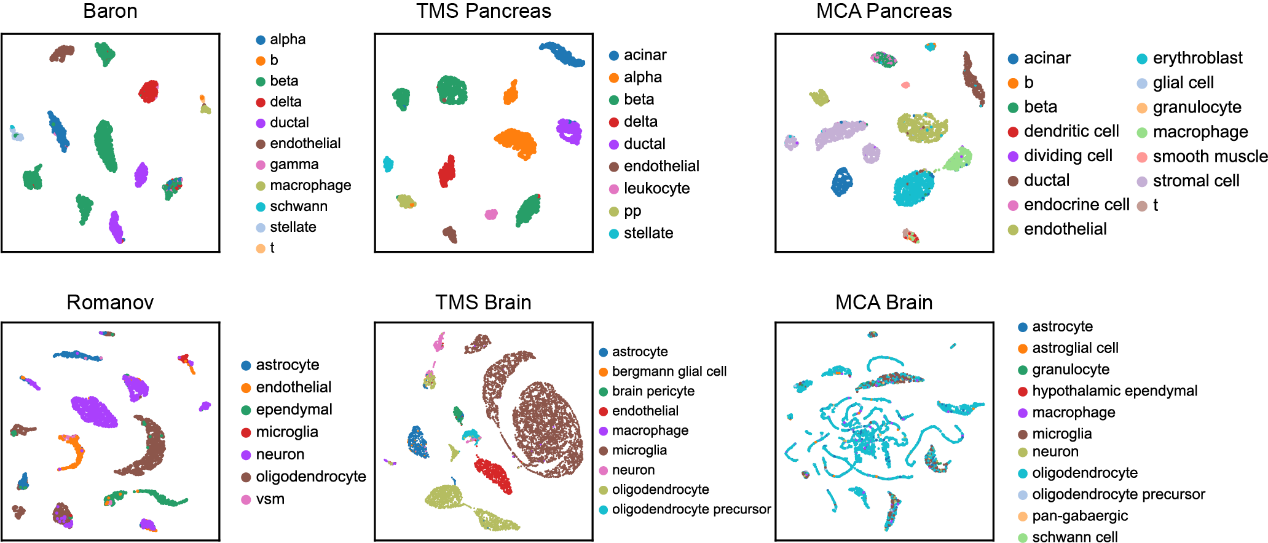


**Fig A. UMAP visualization of latent representation in mouse Brain and mouse Pancreas datasets generated by scDeepCluster.**


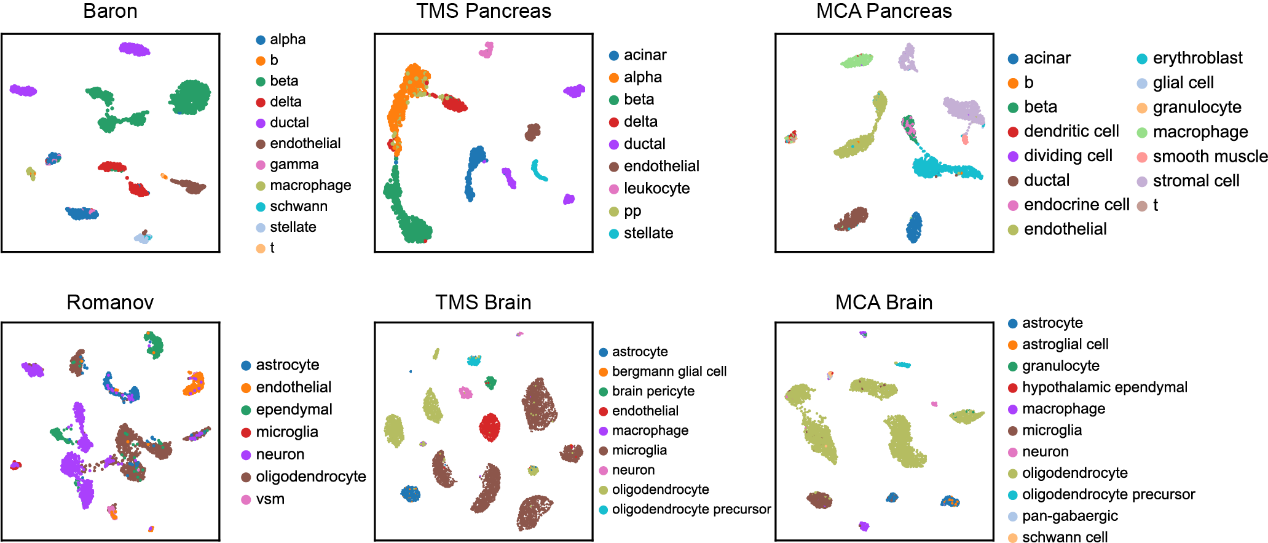


**Fig B. UMAP visualization of latent representation in mouse Brain and mouse Pancreas datasets generated by DESC.**


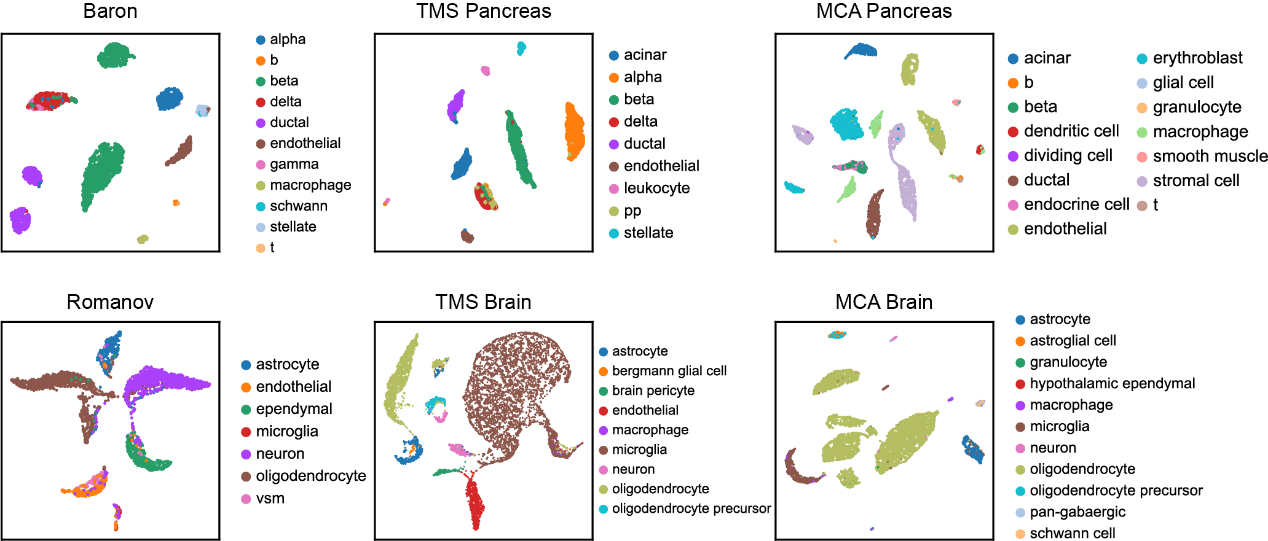


**Fig C. UMAP visualization of latent representation in mouse Brain and mouse Pancreas datasets generated by scDCC.**
