# Supplementary material for: scTrans: Sparse attention powers fast and accurate cell type annotation in single-cell RNA-seq data
Source: PLoS Comput Biol. 2025 Apr 4;21(4):e1012904. doi: 10.1371/journal.pcbi.1012904 (PMC11970913; doi:10.1371/journal.pcbi.1012904)
Supplement: S15 Fig — UMAP visualization of T cell development analysis results. (A–C) UMAP visualization of latent representations generated by scTrans, scVI and trVAE for T cell development datasets, including clustering results, dpt pseudotime inference results, and expression of DEG in development stage. (D) The expression variations of four specific genes based on the pseudo time results inferred from scTrans, scVI, and trVAE. (DOCX) [file pcbi.1012904.s015.docx]

**S15 Fig. UMAP visualization of T cell development analysis results. Fig A-C. UMAP visualization of latent representations generated by scTrans, scVI and trVAE for T cell development datasets, including clustering results, dpt pseudotime inference results, and expression of DEG in development stage. Fig D. The expression variations of four specific genes based on the pseudo time results inferred from scTrans, scVI, and trVAE.s**


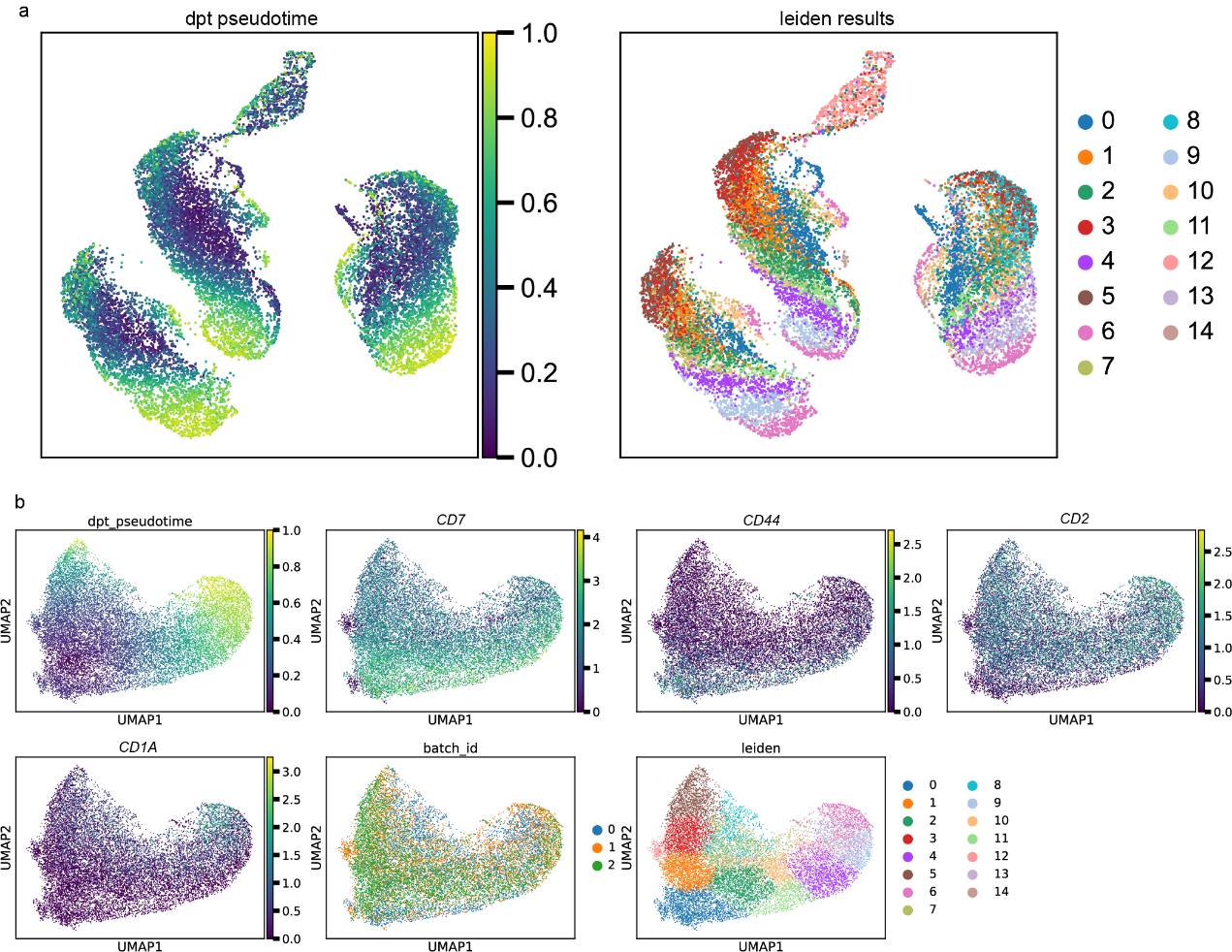


**Fig A. Leiden clustering results, dpt pseudotime inferred results and specific gene expression of the T cell development dataset based on latent representation generated by scTrans.** (a) UMAP visualization based on PCA embedding of T cell development datasets, including dpt pseudotime and Leiden clustering results inferred by scTrans. (b) UMAP visualization based on latent representation generated by scTrans, including dpt pseudotime, the expression of DEG, batch information, and Leiden clustering results.


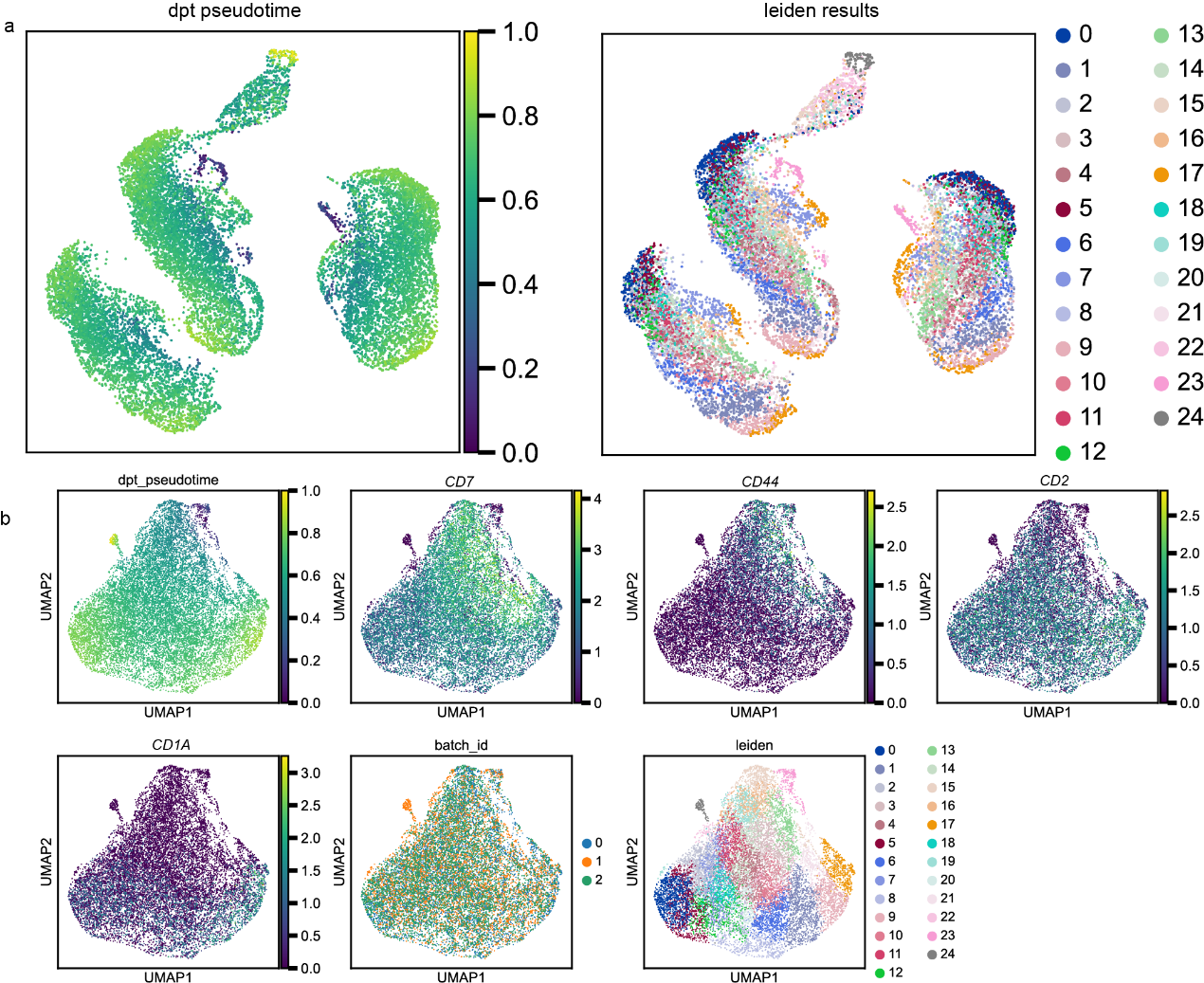


**Fig B. Leiden clustering results, dpt pseudotime inferred results and specific gene expression of the T cell development dataset based on latent representation generated by trVAE.** (a) UMAP visualization based on PCA embedding of T cell development datasets, including dpt pseudotime and Leiden clustering results inferred by trVAE. (b) UMAP visualization based on latent representation generated by trVAE, including dpt pseudotime, the expression of DEG, batch information, and Leiden clustering results.


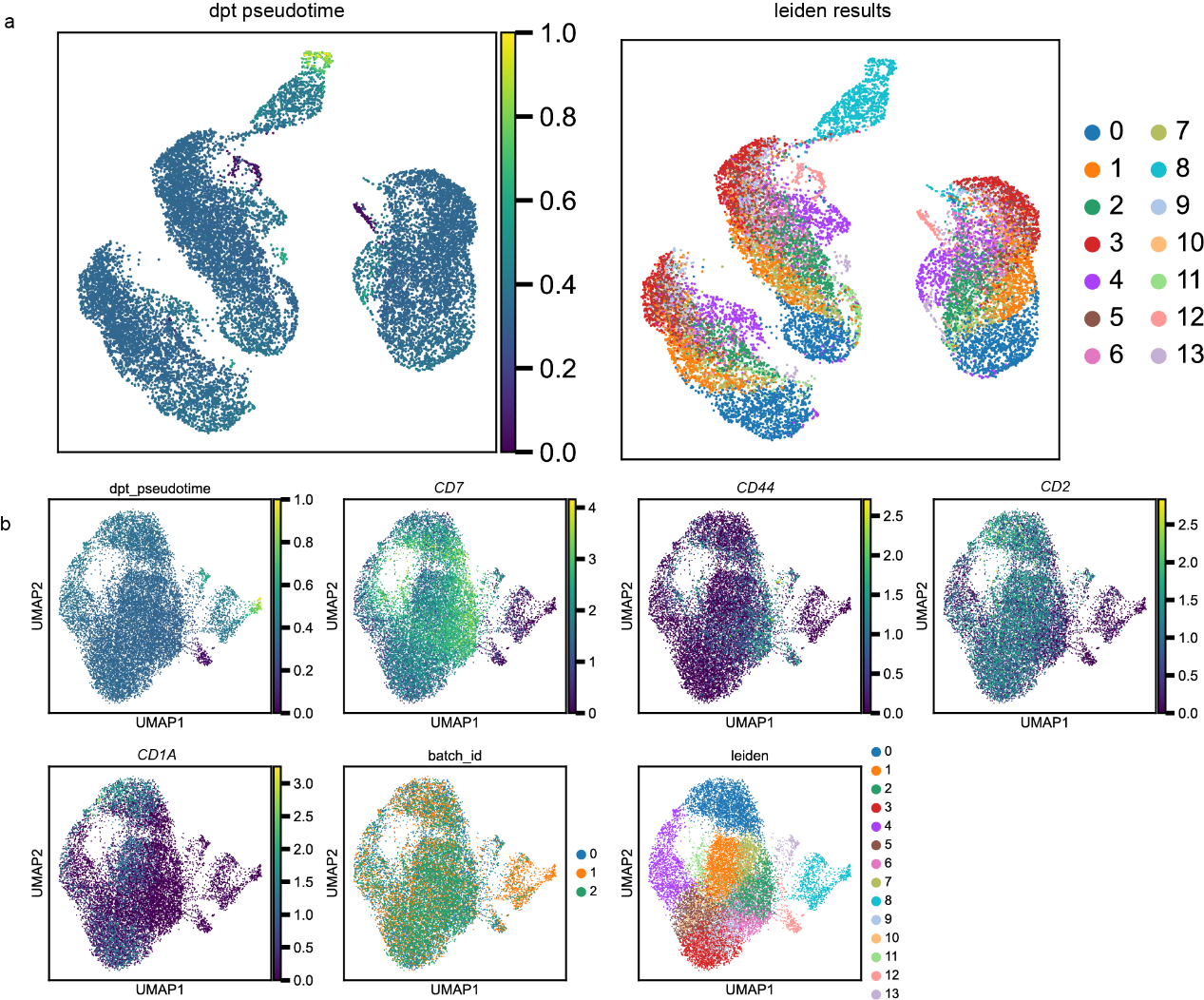


**Fig C. Leiden clustering results, dpt pseudotime inferred results and specific gene expression of the T cell development dataset based on latent representation generated by scVI.** (a) UMAP visualization based on PCA embedding of T cell development datasets, including dpt pseudotime and Leiden clustering results inferred by scVI. (b) UMAP visualization based on latent representation generated by scVI, including dpt pseudotime, the expression of DEG, batch information, and Leiden clustering results.


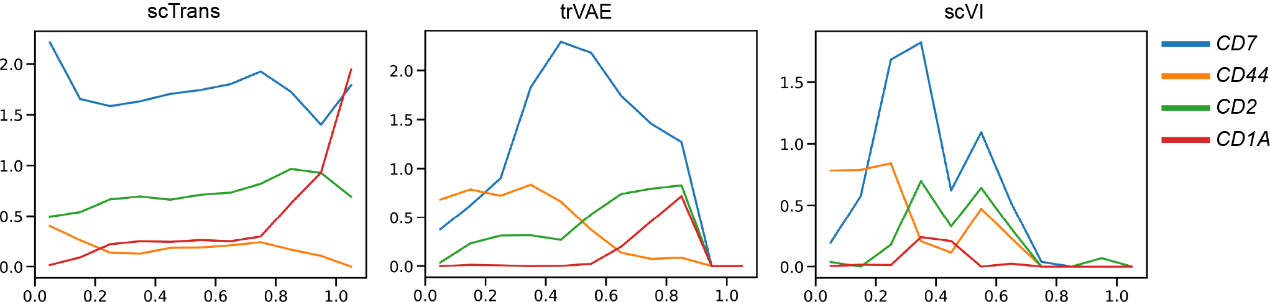


**Fig D. The expression variations of four specific genes based on the pseudo time results.** The x-axis is the pseudo time, while the y-axis is the average gene expression of all cells at the current pseudo time. Ten points were taken from 0 to 1 on the x-axis, with a step size of 0.1. To obtain a smoother gene expression curve, we averaged the gene expression over a pseudo time period for display. For example, for the 0.1 point, we selected the pseudo time between 0.05 and 0.15 for gene expression and took the average for drawing curve.
