# Supplementary material for: scTrans: Sparse attention powers fast and accurate cell type annotation in single-cell RNA-seq data
Source: PLoS Comput Biol. 2025 Apr 4;21(4):e1012904. doi: 10.1371/journal.pcbi.1012904 (PMC11970913; doi:10.1371/journal.pcbi.1012904)
Supplement: S16 Fig — UMAP visualization of mouse DC cell development analysis results. (A–C) UMAP visualization results of latent representation generated by scTrans, scDeepCluster and DESC, including cell type, inferred pseudo time result, two gene expression, Ly6c2 and Siglec-H. (DOCX) [file pcbi.1012904.s016.docx]

**S16 Fig. UMAP visualization of mouse DC cell development analysis results. Fig A-C. UMAP visualization results of latent representation generated by scTrans, scDeepCluster and DESC, including cell type, inferred pseudo time result, two gene expression, Ly6c2 and Siglec-H.**


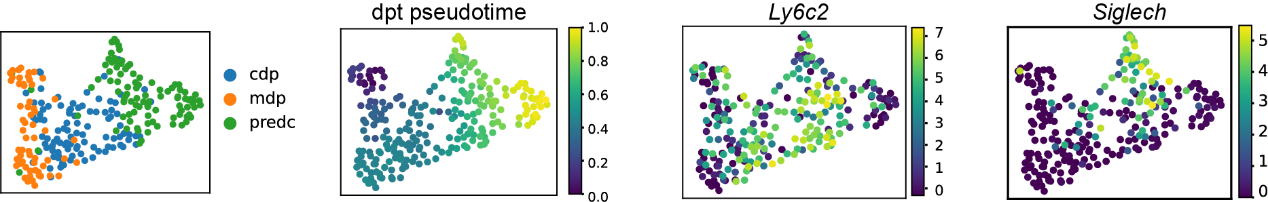


**Fig A. UMAP visualization results of latent representation generated by scTrans, including cell type, inferred pseudo time result, two gene expression, Ly6c2 and Siglec-****H.**


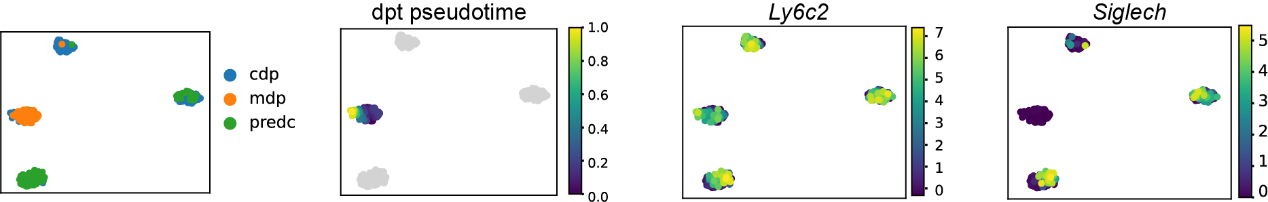


**Fig B. UMAP visualization results of latent representation generated by scDeepCluster, including cell type, inferred pseudo time result, two gene expression, Ly6c2 and Siglec-H.**


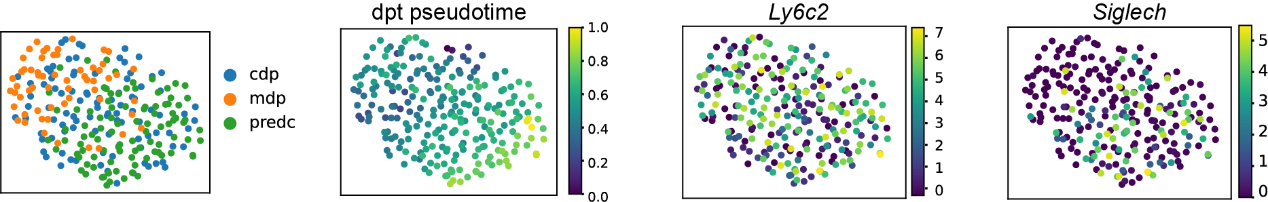


**Fig C. UMAP visualization results of latent representation generated by DESC, including cell type, inferred pseudo time result, two gene expression, Ly6c2 and Siglec-H.**
