# Supplementary material for: scTrans: Sparse attention powers fast and accurate cell type annotation in single-cell RNA-seq data
Source: PLoS Comput Biol. 2025 Apr 4;21(4):e1012904. doi: 10.1371/journal.pcbi.1012904 (PMC11970913; doi:10.1371/journal.pcbi.1012904)
Supplement: S17 Fig — Analysis of batch correction and cell subtype annotation results in PBMC45k dataset. (A) Cell type ASW and batch ASW results computed based on scTrans, scVI and trVAE. (B) Annotation heatmap results of scTrans, scSemiGAN and scDeepSort in PBMC45k by using PBMC160k dataset as reference. (C) Expression of top 10 critical genes in B cell subtypes identified by scTrans. (D) Expression of top 10 critical genes in dendritic cell subtypes identified by scTrans. (E) Expression of marker genes in dendritic cell subtypes. (DOCX) [file pcbi.1012904.s017.docx]

**S17 Fig.** **Analysis of Batch Correction and Cell Subtype Annotation Results in PBMC45k Dataset. Fig A. Cell type ASW and batch ASW results computed based on scTrans, scVI and trVAE. Fig.B Annotation heatmap results of scTrans, scSemiGAN and scDeepSort in PBMC45k by using PBMC160k dataset as reference. Fig.C Expression of top 10 critical genes in B cell subtypes identified by scTrans. Fig D. Expression of top 10 critical genes in dendritic cell subtypes identified by scTrans. Fig E. Expression of marker genes in dendritic cell subtypes.**


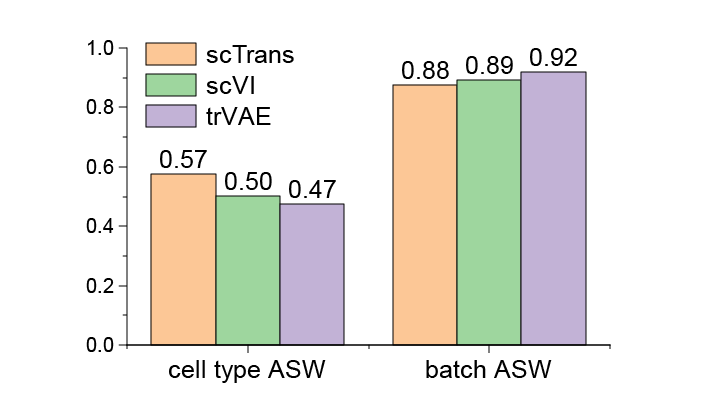


**Fig A. Cell type ASW and batch ASW results of PBMC45k.** scVI and trVAE use PBMC45k technology type as batch information for batch correct, and corrected representation of cells are used to calculate cell type ASW and batch ASW. scTrans trained on the PBMC160K dataset, then extract the representations of PBMC45K for computing cell type ASW and batch ASW.


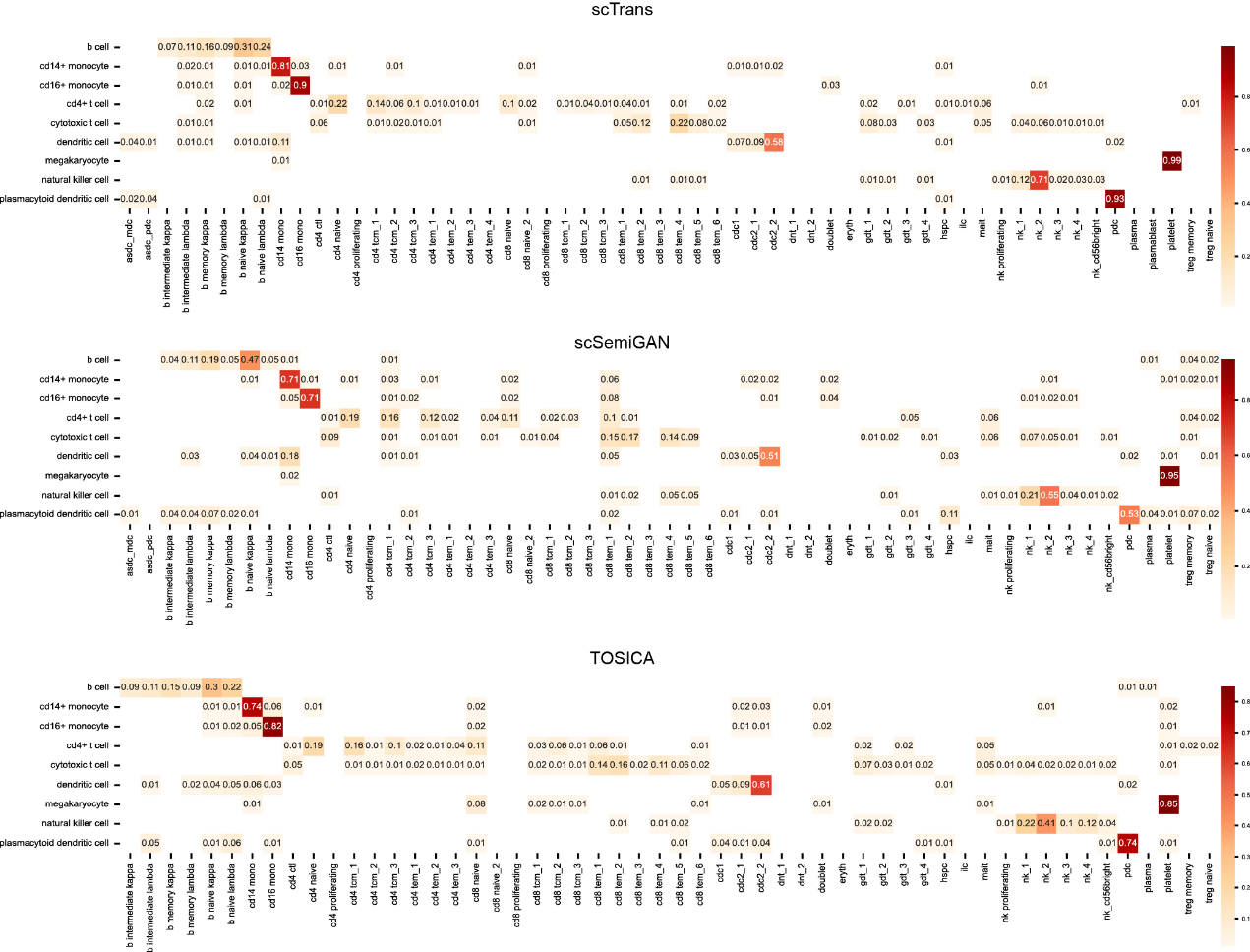


**Fig B. Annotation heatmap results of PBMC45k by using PBMC160k dataset as reference.** scTrans, scSemiGAN, and TOSICA use PBMC160k as a reference to annotate the PBMC45K dataset. The x-axis is the predicted cell type, and the y-axis is the real cell type. Each row represents the percentage of real cell types mapped to which predicted cell types, values less than 0.01 are filtered.Fig C. Leiden clustering results, dpt pseudotime inferred results and specific gene expression of the T cell development dataset based on latent representation generated by scVI. (a) UMAP visualization based on PCA embedding of T cell development datasets, including dpt pseudotime and Leiden clustering results inferred by scVI. (b) UMAP visualization based on latent representation generated by scVI, including dpt pseudotime, the expression of DEG, batch information, and Leiden clustering results.


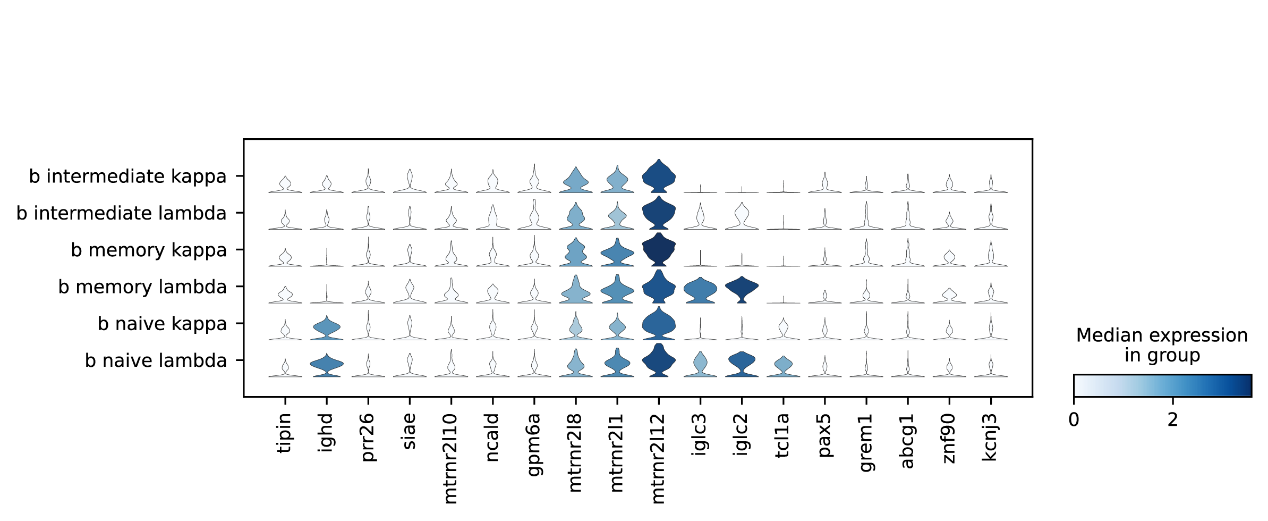


**Fig C. Expression of top 10 critical genes in B cell subtypes identified by scTrans.** The x-axis is the critical genes, and the y-axis is B cell subtypes. The color of the violin represents the median expression of genes in cell subtypes, while the shape of the violin represents the distribution of genes in cell subtypes.


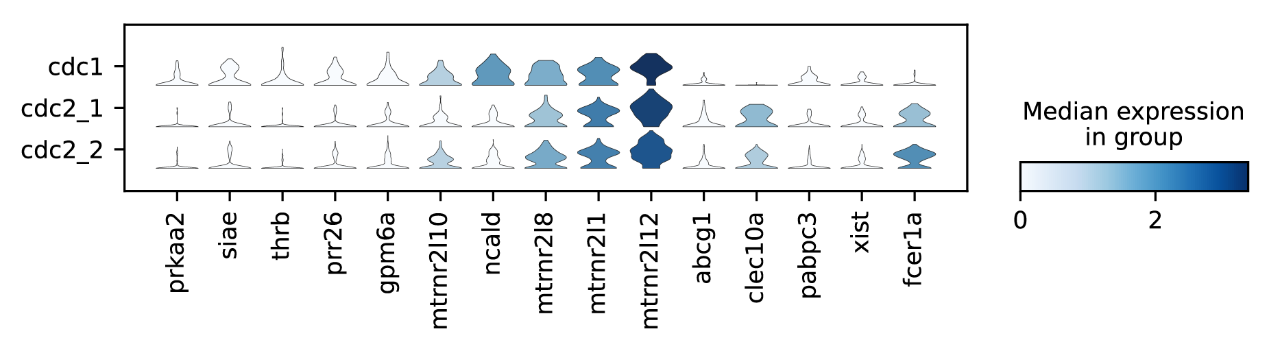


**Fig D. Expression of top 10 critical genes in dendritic cell subtypes identified by scTrans.** The x-axis is the critical genes, and the y-axis is dendritic cell subtypes. The color of the violin represents the median expression of genes in cell subtypes, while the shape of the violin represents the distribution of genes in cell subtypes.


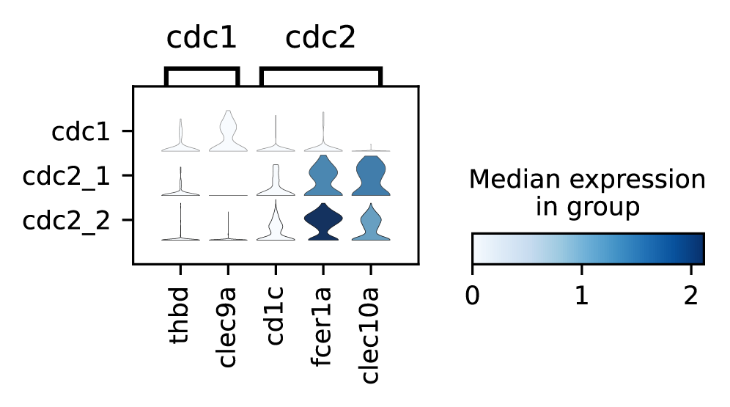


**Fig E. Expression of marker genes in dendritic cell subtypes.** The x-axis is two marker genes of cdc1 and cdc2 cells, the y-axis is dendritic cell subtypes. The color of the violin represents the median expression of genes in cell subtypes, while the shape of the violin represents the distribution of genes in cell subtypes.
