# Supplementary material for: scTrans: Sparse attention powers fast and accurate cell type annotation in single-cell RNA-seq data
Source: PLoS Comput Biol. 2025 Apr 4;21(4):e1012904. doi: 10.1371/journal.pcbi.1012904 (PMC11970913; doi:10.1371/journal.pcbi.1012904)
Supplement: S1 Table — The number of cells and cell types of 31 tissues datasets in MCA datasets. (DOCX) [file pcbi.1012904.s018.docx]

**S1 Table: The number of cells and cell types of 31 tissues datasets in MCA datasets.**

| **Tissue** | **cell number** | **cell type number** |
| --- | --- | --- |
| Muscle | 1102 | 11 |
| Spleen | 1970 | 10 |
| Stomach | 2389 | 12 |
| Prostate | 2505 | 6 |
| Fetal liver | 2699 | 9 |
| Bladder | 2746 | 13 |
| Embryonic mesenchyme | 2771 | 15 |
| Neonatal skin | 3392 | 17 |
| Pancreas | 3610 | 15 |
| Uterus | 3739 | 12 |
| Neonatal heart | 3948 | 13 |
| Brain | 4038 | 11 |
| Thymus | 4289 | 7 |
| Placenta | 4346 | 23 |
| Ovary | 4363 | 10 |
| Fetal brain | 4369 | 16 |
| Neonatal pancreas | 4571 | 15 |
| Kidney | 4682 | 20 |
| Liver | 4685 | 14 |
| Neonatal muscle | 4873 | 20 |
| Fetal intestine | 6076 | 11 |
| Fetal stomach | 6192 | 15 |
| Neonatal rib | 6262 | 14 |
| Fetal lung | 6453 | 6 |
| Small intestine | 6684 | 12 |
| Lung | 6940 | 24 |
| Peripheral blood | 7095 | 9 |
| Neonatal calvaria | 7964 | 11 |
| Testis | 14005 | 10 |
| Bone marrow | 26993 | 17 |
| Mammary gland | 28648 | 19 |
