# Supplementary material for: scTrans: Sparse attention powers fast and accurate cell type annotation in single-cell RNA-seq data
Source: PLoS Comput Biol. 2025 Apr 4;21(4):e1012904. doi: 10.1371/journal.pcbi.1012904 (PMC11970913; doi:10.1371/journal.pcbi.1012904)
Supplement: S4 Table — The mean and standard deviation of accuracy and f1-macro based on 31 tissues in MCA datasets at different train rate. (DOCX) [file pcbi.1012904.s021.docx]

**S4 Table. The mean and standard deviation of accuracy and f1-macro based on 31 tissues in MCA datasets at different train rate.** Different training rates indicate the use of labeled data with different proportions for training. For example, 0.1 represents training using 10% of labeled data from each organizational dataset, with the remaining 90% used for prediction. All methods were run five times with random seeds, and the model performance was represented using the mean and standard deviation. Best result is displayed in bold, and the second-best result is underlined.

**Table A. The mean and standard deviation of accuracy based on 31 tissues in MCA datasets at different train rate.**

| **Methods** | **Train Rate** | | |
| --- | --- | --- | --- |
|  | 0.01 | 0.05 | 0.1 |
| scTrans | **81.32%±9.3%** | **87.22%±8.57%** | **89.15%±7.79%** |
| scDeepSort | 65.14%±14.01% | 79.03%±10.14% | 83.39%±8.81% |
| Concerto | 32.11%±20.99% | 63.69%±18.68% | 78.25%±14.03% |
| itclust | 60.39%±14.75% | 65.05%±20.15% | 65.95%±20.99% |
| scSemiGAN | 68.8%±12.19% | 79.14%±9.55% | 81.80%±8.66% |
| TOSICA | 38.86%±18.08% | 39.57%±18.09% | 39.85%±18.56% |

**Table B. The mean and standard deviation of f1-macro based on 31 tissues in MCA datasets at different train rate.**

| **Methods** | **Train Rate** | | |
| --- | --- | --- | --- |
|  | 0.01 | 0.05 | 0.1 |
| scTrans | **50.67%±12.66%** | **69.44%±14.44%** | **77.5%±12.13%** |
| scDeepSort | 26.97%±9.84% | 49.72%±14.15% | 60.72%±13.97% |
| Concerto | 5.87%±5.64% | 33.03%±20.33% | 58.53%±20.87% |
| itclust | 32.5%±13.23% | 43.2%±17.19% | 46.77%±18.37% |
| scSemiGAN | 34.05%±9.33% | 48.58%±11.17% | 53.75%±11.85% |
| TOSICA | 4.85%±3.19% | 5.09%±3.26% | 5.3%±3.76% |
