# Supplementary material for: scTrans: Sparse attention powers fast and accurate cell type annotation in single-cell RNA-seq data
Source: PLoS Comput Biol. 2025 Apr 4;21(4):e1012904. doi: 10.1371/journal.pcbi.1012904 (PMC11970913; doi:10.1371/journal.pcbi.1012904)
Supplement: S5 Table — Performance evaluation of scTrans trained with different levels of cell type annotations. (DOCX) [file pcbi.1012904.s022.docx]

**S5. Performance evaluation of scTrans trained with different levels of cell type annotations.**

|  | Level1 | Level2 | Level3 |
| --- | --- | --- | --- |
| **Accuracy** | 98.17%±0.07% | 93.67%±0.28% | 87.56%±0.10% |
