# Supplementary material for: scTrans: Sparse attention powers fast and accurate cell type annotation in single-cell RNA-seq data
Source: PLoS Comput Biol. 2025 Apr 4;21(4):e1012904. doi: 10.1371/journal.pcbi.1012904 (PMC11970913; doi:10.1371/journal.pcbi.1012904)
Supplement: S6 Table — Comparison results with large models. (DOCX) [file pcbi.1012904.s023.docx]

**S6 Table: Comparison results with large models**

|  | scTrans | scGPT-zero-shot | scGPT-fine-tune | CellPLM-zero-shot | CellPLM-fine-tune |
| --- | --- | --- | --- | --- | --- |
| Accuracy | **87.55%** | 71.02% | 81.29% | 69.89% | 85.65% |

**Accuracy of annotation results on PBMC160k datasets.**

| Technology | scTrans | CellPLM- zero-shot | CellPLM- fine-tune | scGPT-zero-shot | scGPT- fine-tune |
| --- | --- | --- | --- | --- | --- |
| 10x (v2) A | 83.87% | **87.85%** | 86.79% | 85.99% | 82.56% |
| inDrops | 85.07% | 86.11% | **87.11%** | 87.04% | 84.44% |
| Seq-Well | 78.11% | **80.30%** | 77.95% | 80.04% | 78.78% |
| 10x (v2) | 84.48% | 84.72% | **84.95%** | 83.38% | 81.29% |
| CEL-Seq2 | 83.57% | 80.51% | **84.61%** | 77.71% | 35.03% |
| 10x (v3) | 78.16% | 82.95% | 84.39% | **85.36%** | 81.78% |
| Smart-seq2 | **83.37%** | 81.91% | 83.21% | 74.97% | 29.15% |
| 10x (v2) B | 82.34% | 85.54% | 84.84% | **85.47%** | 81.59% |
| Drop-seq | 80.01% | **84.38%** | 80.75% | 73.54% | 71.59% |

**Accuracy of annotation results in single reference task on PBMC45K datasets.**

| **Technology** | **scTrans** | **CellPLM- zero-shot** | **CellPLM- fine-tune** | **scGPT-zero-shot** | **scGPT- fine-tune** |
| --- | --- | --- | --- | --- | --- |
| 10x (v2) A | 90.25% | 91.99% | **92.45%** | 91.96% | 91.03% |
| inDrops | 83.24% | 86.33% | **86.52%** | 85.92% | 86.33% |
| Seq-Well | 79.13% | 79.26% | 78.45% | **80.12%** | 79.50% |
| 10x (v2) | 91.7% | **95.57%** | 89.58% | 92.12% | 93.99% |
| CEL-Seq2 | 78.59% | 69.96% | **79.27%** | 78.33% | 80.03% |
| 10x (v3) | 91.46% | 92.21% | **93.35%** | 92.15% | 90.28% |
| Smart-seq2 | 85.67% | 84.03% | **85.93%** | 85.17% | 85.74% |
| 10x (v2) B | 89.59% | 89.20% | 90.00% | 89.94% | **90.25%** |
| Drop-seq | 84.46% | **86.95%** | 77.76% | 83.67% | 83.93% |

**Accuracy of annotation results in multi reference task on PBMC45K datasets.**
