# Supplementary material for: scTrans: Sparse attention powers fast and accurate cell type annotation in single-cell RNA-seq data
Source: PLoS Comput Biol. 2025 Apr 4;21(4):e1012904. doi: 10.1371/journal.pcbi.1012904 (PMC11970913; doi:10.1371/journal.pcbi.1012904)
Supplement: S7 Table — Comparison results of PCA embedding initialization and large model embedding initialization. (DOCX) [file pcbi.1012904.s024.docx]

**S7 Table: Comparison results of PCA embedding initialization and large model embedding initialization.**

|  | scTrans-pca | scTrans-cellPLM | scTrans-scGPT |
| --- | --- | --- | --- |
| Accuracy | **87.55%** | 83.54% | 83.28% |

**Table A. Comparison results of embedding initialization on the PBMC160k dataset**

| Batch | scTrans-pca | scTrans-cellPLM | scTrans-scGPT |
| --- | --- | --- | --- |
| 10x (v2) A | 83.87% | **84.75%** | 82.47% |
| inDrops | 85.07% | **85.61%** | 85.20% |
| Seq-Well | 78.11% | 80.93% | **82.78%** |
| 10x (v2) | **84.48%** | 84.35% | 79.62% |
| CEL-Seq2 | **83.57%** | 77.34% | 73.10% |
| 10x (v3) | 78.16% | **84.28%** | 81.65% |
| Smart-seq2 | **83.37%** | 71.45% | 64.08% |
| 10x (v2) B | 82.34% | **85.15%** | 84.79% |
| Drop-seq | 80.01% | **83.40%** | 77.19% |

**Table B. Comparison results of embedding initialization on the single reference task of the PBMC45k dataset**

| Batch | scTrans-pca | scTrans-cellPLM | scTrans-scGPT |
| --- | --- | --- | --- |
| 10x (v2) A | 90.25% | **93.85%** | 91.49**%** |
| inDrops | 83.24% | 86.45% | **86.92%** |
| Seq-Well | 79.13% | 81.16% | **81.83%** |
| 10x (v2) | 91.7% | **94.05%** | 88.22**%** |
| CEL-Seq2 | 78.59% | **82.88%** | 71.48**%** |
| 10x (v3) | 91.46% | **92.33%** | 91.58**%** |
| Smart-seq2 | 85.67% | **85.73%** | 73.00**%** |
| 10x (v2) B | 89.59% | **91.58%** | 89.29**%** |
| Drop-seq | 84.46% | 87.06% | **87.10%** |

**Table C. Comparison results of embedding initialization on the multi reference task of the PBMC45k dataset**
