# Supplementary material for: scTrans: Sparse attention powers fast and accurate cell type annotation in single-cell RNA-seq data
Source: PLoS Comput Biol. 2025 Apr 4;21(4):e1012904. doi: 10.1371/journal.pcbi.1012904 (PMC11970913; doi:10.1371/journal.pcbi.1012904)
Supplement: S8 Table — Details of PBMC45k. (DOCX) [file pcbi.1012904.s025.docx]

**S8 Table. Details of PBMC45k.** PBMC45k dataset consists of 9 batches, 7 techniques, and 2 experiments.

| **Technique** | **Experiment** | **Cell number** |
| --- | --- | --- |
| 10x Chromium (v2) | pbmc2 | 3362 |
| 10x Chromium (v2) A | pbmc1 | 3222 |
| 10x Chromium (v2) B | pbmc1 | 3222 |
| 10x Chromium (v3) | pbmc1 | 3222 |
| CEL-Seq2 | pbmc1/pbmc2 | 526 |
| Drop-seq | pbmc1/pbmc2 | 6584 |
| inDrops | pbmc1/pbmc2 | 6584 |
| Seq-Well | pbmc1/pbmc2 | 3727 |
| Smart-seq2 | pbmc1/pbmc2 | 526 |
