# Supplementary material for: scTrans: Sparse attention powers fast and accurate cell type annotation in single-cell RNA-seq data
Source: PLoS Comput Biol. 2025 Apr 4;21(4):e1012904. doi: 10.1371/journal.pcbi.1012904 (PMC11970913; doi:10.1371/journal.pcbi.1012904)
Supplement: S9 Table — Cell type details of three mouse brain and three mouse pancreas datasets. (DOCX) [file pcbi.1012904.s026.docx]

**S9 Table. Cell type details of three mouse brain and three mouse pancreas datasets.** The table displays the composition of cell types and the number of cells in different datasets.

**Table A. Cell type details of three mouse brain datasets.** These datasets from different laboratories, there are differences in cell type composition. The first column is the cell type name, and the remaining columns are the number of cell types in the corresponding dataset

| **Cell type** | **TMS-Brain** | **MCA-Brain** | **Romanov** |
| --- | --- | --- | --- |
| endothelial | 715 | 0 | 240 |
| Pan-GABAergic | 0 | 26 | 0 |
| brain pericyte | 156 | 0 | 0 |
| microglia | 4394 | 338 | 48 |
| Bergmann glial cell | 40 | 0 | 0 |
| Hypothalamic ependymal | 0 | 9 | 0 |
| vsm | 0 | 0 | 71 |
| oligodendrocyte | 1574 | 3150 | 1001 |
| astrocyte | 432 | 199 | 267 |
| Granulocyte | 0 | 26 | 0 |
| oligodendrocyte precursor | 203 | 72 | 0 |
| ependymal | 0 | 0 | 356 |
| neuron | 281 | 48 | 898 |
| Schwann cell | 0 | 16 | 0 |
| Astroglial cell | 0 | 30 | 0 |
| macrophage | 61 | 124 | 0 |

**Table B. Cell type details of three mouse pancreas datasets.** These datasets from different laboratories, there are differences in cell type composition. The first column is the cell type name, and the remaining columns are the number of cell types in the corresponding dataset

| **Cell type** | **TMS-Pancreas** | **MCA-Pancreas** | **Baron** |
| --- | --- | --- | --- |
| Dendritic cell | 0 | 34 | 0 |
| Erythroblast | 0 | 817 | 0 |
| beta | 449 | 125 | 894 |
| Glial cell | 0 | 11 | 0 |
| Granulocyte | 0 | 13 | 0 |
| stellate | 49 | 0 | 61 |
| T | 0 | 39 | 7 |
| delta | 140 | 0 | 218 |
| acinar | 182 | 260 | 0 |
| leukocyte | 54 | 0 | 0 |
| Stromal cell | 0 | 778 | 0 |
| B | 0 | 16 | 10 |
| schwann | 0 | 0 | 6 |
| Dividing cell | 0 | 12 | 0 |
| Endocrine cell | 0 | 82 | 0 |
| endothelial | 66 | 719 | 139 |
| Smooth muscle cell | 0 | 45 | 0 |
| macrophage | 0 | 278 | 36 |
| alpha | 390 | 0 | 191 |
| pp | 73 | 0 | 0 |
| gamma | 0 | 0 | 41 |
| ductal | 161 | 381 | 275 |
