# Supplementary material for: scTrans: Sparse attention powers fast and accurate cell type annotation in single-cell RNA-seq data
Source: PLoS Comput Biol. 2025 Apr 4;21(4):e1012904. doi: 10.1371/journal.pcbi.1012904 (PMC11970913; doi:10.1371/journal.pcbi.1012904)
Supplement: S10 Table — Accuracy and fi-macro of annotation results in single reference and multi reference task on PBMC45K datasets. (DOCX) [file pcbi.1012904.s027.docx]

**S10 Table. Accuracy and fi-macro of annotation results in single reference and multi reference task on PBMC45K datasets.** All methods were run five times with random seeds, and the model performance was represented using the mean and standard deviation. Average is the average performance results of all technologies. Best result is displayed in bold, and the second-best result is underlined.

**Table A. Accuracy of annotation results in single reference task on PBMC45K datasets.**

| **Technology** | **scTrans** | **scDeepSort** | **Concerto** | **itclust** | **scSemiGAN** | **TOSICA** |
| --- | --- | --- | --- | --- | --- | --- |
| 10x (v2) A | 83.87%±1.57% | **85.15%±0.14%** | 43.78%±5.19% | 81.44%±0.24% | 68.06%±0.7% | 68.75%±1.38% |
| 10x (v2) B | 82.34%±0.51% | **83.81%±0.32%** | 39.95%±5.27% | 76.64%±1.74% | 66.93%±0.48% | 43.26%±17.4% |
| 10x (v2) | **84.48%±0.38%** | 83.96%±0.14% | 81.71%±0.79% | 75.84%±0.24% | 68.67%±0.7% | 40.27%±19.3% |
| 10x (v3) | 78.16%±1.44% | **83.94%±0.19%** | 54.53%±3.22% | 79.23%±0.57% | 65.08%±1.27% | 65.94%±2.32% |
| CEL-Seq2 | **83.57%±0.56%** | 74.69%±1.17% | 59.77%±2.81% | 76.06%±2.17% | 58.02%±2.52% | 29.22%±0% |
| Drop-seq | **80.01%±1.93%** | 79.93%±0.59% | 56.08%±2.74% | 71.56%±0.96% | 64.4%±1.82% | 71.69%±5.13% |
| inDrops | 85.07%±0.98% | **86.57%±0.17%** | 70.35%±1.99% | 74.74%±0.69% | 70.04%±0.64% | 72.97%±1.51% |
| Seq-Well | 78.11%±1.34% | 75.77%±0.29% | 77.61%±0.61% | **78.87%±0.33%** | 64.68%±0.85% | 60.14%±1.29% |
| Smart-seq2 | **83.37%±1.1%** | 78.79%±0.92% | 66.04%±2.3% | 76.45%±1.9% | 59.14%±1.69% | 29.16%±0% |
| Average | **82.11%±2.52%** | 81.4%±4.02% | 60.9%±13.99% | 76.76%±2.69% | 65%±3.88% | 53.49%±17.0% |

**Table B. F1-macro of annotation results in single reference task on PBMC45K datasets.**

| **Technology** | **scTrans** | **scDeepSort** | **Concerto** | **itclust** | **scSemiGAN** | **TOSICA** |
| --- | --- | --- | --- | --- | --- | --- |
| 10x (v2) A | 80.58%±1.49% | **82.98%±0.19%** | 38.16%±5.17% | 76.08%±0.29% | 57.09%±1.36% | 33.91%±1.97% |
| 10x (v2) B | 80.27%±0.85% | **82.1%±0.29%** | 35.15%±3.93% | 65.26%±3.17% | 56.58%±0.7% | 15.2%±12.55% |
| 10x (v2) | **81.46%±0.15%** | 80.81%±0.25% | 79.4%±0.7% | 69.93%±0.44% | 57.32%±0.92% | 15.85%±12.2% |
| 10x (v3) | 67.23%±0.8% | **72.53%±0.2%** | 43.49%±1.13% | 65.54%±0.75% | 50.59%±2.55% | 35.89%±1.66% |
| CEL-Seq2 | **59.21%±1.55%** | 53.59%±0.62% | 41.83%±2.28% | 56.94%±1.69% | 37.85%±2.42% | 5.03%±0% |
| Drop-seq | 71.95%±2.43% | **72.37%±1.11%** | 51.12%±3.93% | 58.59%±2.01% | 51.88%±1.48% | 34.35%±3.09% |
| inDrops | 75.68%±2.75% | **83.63%±0.33%** | 59.61%±1.77% | 54.72%±0.55% | 55.04%±1.07% | 37.09%±4.32% |
| Seq-Well | **62.93%±0.97%** | 61.89%±0.44% | 62.17%±0.7% | 58.44%±1.15% | 42.65%±1.36% | 27.46%±1.01% |
| Smart-seq2 | **62.75%±1.11%** | 51.62%±1.26% | 39.13%±1.2% | 57.94%±1.43% | 42.83%±0.94% | 5.02%±0% |
| Average | **71.34%±8.13%** | 71.28%±11.96% | 49.85%±14.2% | 62.6%±6.67% | 50.2%±6.91% | 23.31%±12.4% |

**Table C. Accuracy of annotation results in multi reference task on PBMC45K datasets.**

| **Technology** | **scTrans** | **scDeepSort** | **Concerto** | **itclust** | **scSemiGAN** | **TOSICA** |
| --- | --- | --- | --- | --- | --- | --- |
| 10x (v2) A | 90.25%±1.01% | **91.56%±0.27%** | 90.03%±0.88% | 85.83%±0.33% | 81.76%±0.12% | 88.61%±0.43% |
| 10x (v2) B | 89.59%±0.31% | **90.59%±0.1%** | 82.24%±2.88% | 88.58%±0.26% | 83.76%±0.28% | 86.29%±0.35% |
| 10x (v2) | 91.7%±0.53% | **92.93%±0.47%** | 83.23%±2.17% | 91.9%±0.19% | 83.5%±0.66% | 89.99%±0.91% |
| 10x (v3) | 91.46%±0.54% | **93.63%±0.25%** | 86.82%±1.9% | 91.24%±0.26% | 87.56%±0.16% | 91.83%±0.26% |
| CEL-Seq2 | 78.59%±2.44% | 82.59%±1.44% | 77.66%±1.17% | **83.19%±0.78%** | 76.01%±0.56% | 83.65%±0.34% |
| Drop-seq | 84.46%±0.73% | 81.59%±1.17% | 83.94%±1.31% | **84.67%±0.28%** | 73.63%±0.34% | 80.85%±1.09% |
| inDrops | 83.24%±1.19% | **85.04%±0.37%** | 80.7%±1.54% | 77.71%±0.59% | 67.38%±0.38% | 81.75%±0.56% |
| Seq-Well | **79.13%±0.44%** | 78.59%±0.25% | 73.04%±5.56% | 67.7%±0.32% | 68.9%±1.02% | 73.98%±0.92% |
| Smart-seq2 | 85.67%±1.87% | **88.48%±0.43%** | 80.2%±1.52% | 84.18%±0.74% | 85.32%±0.39% | 86.92%±0.89% |
| Average | 86.01%±4.78% | **87.22%±5.14%** | 81.98%±4.96% | 83.89%±7.04% | 78.65%±6.97% | 84.87%±5.17% |

**Table D. F1-macro of annotation results in multi reference task on PBMC45K datasets.**

| **Technology** | **scTrans** | **scDeepSort** | **Concerto** | **itclust** | **scSemiGAN** | **TOSICA** |
| --- | --- | --- | --- | --- | --- | --- |
| 10x (v2) A | 89.97%±0.61% | **91.97%±0.51%** | 85.83%±2.64% | 84.95%±0.49% | 77.92%±0.84% | 76.35%±5.8% |
| 10x (v2) B | 90.78%±1.35% | **93.13%±0.35%** | 82.45%±4.19% | 88.29%±2.12% | 84.75%±0.7% | 76.38%±6.54% |
| 10x (v2) | 89.65%±0.9% | **92.54%±0.42%** | 71.5%±4.34% | 90.03%±0.63% | 80.57%±1.32% | 75.39%±7.09% |
| 10x (v3) | 80.21%±0.75% | **81.99%±0.21%** | 72.56%±2.85% | 80.97%±0.55% | 76.59%±0.68% | 83.87%±4.37% |
| CEL-Seq2 | 68.16%±1.87% | 71.3%±0.55% | 52.77%±4.78% | **71.81%±0.94%** | 63.41%±3.95% | 72.28%±1.24% |
| Drop-seq | 73.24%±1.27% | 72.06%±1.37% | 73.12%±2.69% | **71.28%±0.63%** | 57.69%±2.9% | 58.04%±6.25% |
| inDrops | **75.34%±1.34%** | 72.49%±0.76% | 72.59%±3.34% | 66.25%±0.23% | 53.57%±0.88% | 64.09%±3.4% |
| Seq-Well | 57.12%±1.9% | **57.89%±0.68%** | 53.36%±3.17% | 52.97%±0.51% | 45.44%±0.84% | 46.64%±1.6% |
| Smart-seq2 | 66.94%±1.63% | 70.61%±0.34% | 61.43%±4.91% | 68.48%±0.38% | 70.49%±3.63% | **80.57%±5.13%** |
| Average | 76.82%±11.1% | **78.22%±11.65%** | 69.81%±11.7% | 75%±11.38% | 67.83%±12.74% | 70.4%±11.26% |
