# Supplementary material for: scTrans: Sparse attention powers fast and accurate cell type annotation in single-cell RNA-seq data
Source: PLoS Comput Biol. 2025 Apr 4;21(4):e1012904. doi: 10.1371/journal.pcbi.1012904 (PMC11970913; doi:10.1371/journal.pcbi.1012904)
Supplement: S11 Table — Accuracy and fi-macro of annotation results in single reference and multi reference task on mouse brain and mouse pancreas datasets. (DOCX) [file pcbi.1012904.s028.docx]

**S11 Table. Accuracy and fi-macro of annotation results in single reference and multi reference task on mouse brain and mouse pancreas datasets.** All methods were run five times with random seeds, and the model performance was represented using the mean and standard deviation. Best result is displayed in bold, and the second-best result is underlined.

**Table A. Accuracy of annotation results in single reference task on mouse brain and mouse pancreas datasets.**

|  | **scTrans** | **scDeepSort** | **Concerto** | **itclust** | **scSemiGAN** | **TOSICA** |
| --- | --- | --- | --- | --- | --- | --- |
| TMS-Brain | 83.4%±0.99% | **85.66%±0.12%** | 77.36%±0.38% | 61.2%±0.95% | 80.2%±1.09% | 79.59%±1.81% |
| MCA-Brain | 47.84%±8.55% | 46.94%±3.21% | **65.51%±5.48%** | 29.93%±0.75% | 45.93%±3.09% | 61.4%±0.95% |
| Romanov | 81.6%±1.67% | **82.64%±0.8%** | 59.24%±10.1% | 45.05%±0.9% | 75.89%±0.58% | 35.46%±3.08% |
| TMS-Pancreas | 54.51%±0.39% | **55.68%±0.26%** | 54.48%±0.88% | 51.85%±0.1% | 25%±3.45% | 18.59%±0.04% |
| MCA-Pancreas | 36.17%±6.87% | **58.99%±1.6%** | 56.09%±2.9% | 27.19%±22.3% | 50.7%±3.69% | 3.58%±2.92% |
| Baron | 50.92%±1.28% | **52.43%±0.43%** | 40.25%±1.02% | 38.47%±0.44% | 42.76%±1.45% | 14.77%±4.8% |

**Table B. F1-macro of annotation results in single reference task on mouse brain and mouse pancreas datasets.**

|  | **scTrans** | **scDeepSort** | **Concerto** | **itclust** | **scSemiGAN** | **TOSICA** |
| --- | --- | --- | --- | --- | --- | --- |
| TMS-Brain | 30.91%±0.46% | **31.33%±0.12%** | 22.43%±0.64% | 23.06%±0.67% | 26.02%±1.06% | 25.23%±0.84% |
| MCA-Brain | 23.81%±1.58% | 19.8%±1.03% | **24.3%±0.66%** | 13.32%±2.71% | 17.34%±0.93% | 14.98%±0.53% |
| Romanov | **25.29%±0.35%** | 24.93%±0.22% | 15.59%±2.36% | 14.71%±0.65% | 23.36%±1% | 11.21%±0.95% |
| TMS-Pancreas | **24.11%±0.32%** | 23.43%±0.26% | 22.62%±0.21% | 20.97%±0.05% | 11.29%±1.07% | 2.22%±0.73% |
| MCA-Pancreas | 23.68%±1.73% | **27.65%±0.15%** | 27.75%±1.19% | 13.91%±11.0% | 20.07%±1.8% | 1.62%±2.26% |
| Baron | 25.55%±0.64% | **26.56%±0.71%** | 16.98%±0.67% | 14.98%±0.25% | 19.49%±1.61% | 3.15%±2.8% |

**Table C. Accuracy of annotation results in multi reference task on mouse brain and mouse pancreas datasets.**

|  | **scTrans** | **scDeepSort** | **Concerto** | **itclust** | **scSemiGAN** | **TOSICA** |
| --- | --- | --- | --- | --- | --- | --- |
| TMS-Brain | **93.7%±0.09%** | 92.91%±0.43% | 65.64%±7.83% | 38.13%±27.6% | 91.02%±0.18% | 86.77%±4.34% |
| MCA-Brain | **90.85%±0.75%** | 90.49%±0.22% | 76.27%±2.54% | 35.87%±22.1% | 80.74%±3.75% | 84%±3.56% |
| Romanov | 70.47%±1.08% | **72.94%±0.19%** | 72.63%±1.26% | 1.53%±2.52% | 68.38%±0.67% | 71.38%±2.53% |
| TMS-Pancreas | **87.07%±0.72%** | 79.72%±1.17% | 60.82%±3.85% | 1.84%±0.93% | 74.67%±8.38% | 48.94%±8.34% |
| MCA-Pancreas | 41.62%±2.08% | **44.24%±1.04%** | 40.07%±0.95% | 42.33%±1.58% | 39.28%±0.61% | 12.04%±3.97% |
| Baron | 82.59%±7.02% | **86.52%±0.77%** | 80.4%±1.64% | 26.95%±32.4% | 67.06%±1% | 54.64%±17.6% |

**Table D. F1-macro of annotation results in multi reference task on mouse brain and mouse pancreas datasets.**

|  | **scTrans** | **scDeepSort** | **Concerto** | **itclust** | **scSemiGAN** | **TOSICA** |
| --- | --- | --- | --- | --- | --- | --- |
| TMS-Brain | **41.36%±0.95%** | 39.97%±1.44% | 22%±1.85% | 19.16%±9.48% | 39.94%±1.76% | 39.13%±5.06% |
| MCA-Brain | **27.39%±2.94%** | 27.01%±0.4% | 16.65%±0.62% | 14.27%±6.02% | 19.75%±0.81% | 20.13%±1.17% |
| Romanov | 26.12%±1.05% | **33.62%±0.2%** | 28.43%±2.45% | 0.61%±0.95% | 21.99%±2.44% | 33.13%±2.34% |
| TMS-Pancreas | **35.21%±3.04%** | 34.25%±2.4% | 25.14%±1.6% | 0.78%±0.42% | 32.15%±3.5% | 23.24%±4.89% |
| MCA-Pancreas | 20.64%±1.3% | **24.52%±1.72%** | 19.64%±1.6% | 18.67%±0.88% | 16.39%±0.63% | 2.65%±1.23% |
| Baron | 35.45%±2.47% | 33.71%±2.95% | **36.63%±1.88%** | 9.69%±8.85% | 23.94%±3.39% | 19.16%±6.02% |
