# Supplementary material for: scTrans: Sparse attention powers fast and accurate cell type annotation in single-cell RNA-seq data
Source: PLoS Comput Biol. 2025 Apr 4;21(4):e1012904. doi: 10.1371/journal.pcbi.1012904 (PMC11970913; doi:10.1371/journal.pcbi.1012904)
Supplement: S12 Table — Clustering performance results in mouse brain and mouse pancreas datasets. (DOCX) [file pcbi.1012904.s029.docx]

**S12 Table. Clustering performance results in mouse brain and mouse pancreas datasets.** All methods were run five times with random seeds, and the model performance was represented using the mean and standard deviation. scTrans, scDeepSort and scDeepSort are trained on reference datasets in multireference task, and extract representation of query datasets for clustering. scDeepCluster and DESC are unsupervised methdos. scDcc is a semi supervised clustering method that uses 10% of labeled data. Best result is displayed in bold, and the second-best result is underlined.

**Table A. ARI of clustering results in mouse brain and mouse pancreas dataset.**

|  | **scTrans** | **scSemiGAN** | **scDeepSort** | **scDeepCluster** | **DESC** | **scDCC** |
| --- | --- | --- | --- | --- | --- | --- |
| TMS-Brain | 0.83±0.04 | 0.42±0.11 | **0.9±0** | 0.77±0.02 | 0.85±0.01 | 0.84±0.02 |
| MCA-Brain | **0.91±0.01** | 0.31±0.18 | 0.03±0 | 0.67±0.02 | 0.77±0.1 | 0.88±0.01 |
| Romanov | **0.93±0.02** | 0.55±0.06 | 0.41±0.00 | 0.45±0.07 | 0.46±0.05 | 0.60±0.02 |
| TMS-Pancreas | **0.62±0.13** | 0.52±0.07 | 0.52±0 | 0.15±0.03 | 0.17±0.04 | 0.25±0.04 |
| MCA-Pancreas | **0.96±0** | 0.9±0.02 | 0.94±0 | 0.58±0.05 | 0.37±0.03 | 0.78±0.04 |
| Baron | 0.57±0.01 | 0.46±0.13 | 0.56±0 | 0.44±0.01 | 0.39±0.06 | **0.63±0.01** |

**Table B. NMI of clustering results in mouse brain and mouse pancreas dataset.**

|  | **scTrans** | **scSemiGAN** | **scDeepSort** | **scDeepCluster** | **DESC** | **scDCC** |
| --- | --- | --- | --- | --- | --- | --- |
| TMS-Brain | 0.81±0.02 | 0.51±0.07 | **0.87±0** | 0.83±0.01 | **0.87±0** | 0.81±0.01 |
| MCA-Brain | **0.91±0** | 0.45±0.16 | 0.11±0 | 0.85±0.01 | 0.85±0.05 | 0.89±0 |
| Romanov | **0.89±0.01** | 0.50±0.03 | 0.51±0.00 | 0.72±0.02 | 0.74±0.01 | 0.80±0.01 |
| TMS-Pancreas | 0.5±0.09 | 0.39±0.04 | 0.43±0 | 0.11±0 | 0.49±0.03 | **0.51±0.03** |
| MCA-Pancreas | **0.89±0** | 0.78±0.04 | 0.86±0 | 0.74±0.02 | 0.69±0.01 | 0.86±0.01 |
| Baron | 0.57±0.02 | 0.41±0.07 | 0.53±0 | 0.61±0.01 | 0.59±0.02 | **0.68±0.01** |

**Table C. ASW of clustering results in mouse brain and mouse pancreas dataset.**

|  | **scTrans** | **scSemiGAN** | **scDeepSort** | **scDeepCluster** | **DESC** | **scDCC** |
| --- | --- | --- | --- | --- | --- | --- |
| TMS-Brain | 0.59±0.01 | 0.34±0.01 | 0.49±0.01 | **0.92±0.02** | 0.82±0 | 0.88±0.02 |
| MCA-Brain | 0.69±0.02 | 0.45±0.02 | 0.75±0.01 | **0.85±0.02** | 0.66±0.01 | 0.76±0.1 |
| Romanov | 0.58±0.02 | 0.45±0.03 | 0.41±0 | **0.87±0.03** | 0.66±0.04 | 0.82±0.04 |
| TMS-Pancreas | 0.56±0.02 | 0.49±0.04 | 0.71±0.01 | **0.92±0.03** | 0.72±0.05 | 0.91±0.03 |
| MCA-Pancreas | 0.8±0.01 | 0.55±0.04 | 0.66±0 | 0.87±0.02 | 0.76±0.05 | **0.91±0.02** |
| Baron | 0.62±0.02 | 0.47±0.02 | 0.49±0 | **0.85±0.02** | 0.57±0.03 | 0.82±0.02 |
