# Supplementary material for: scTrans: Sparse attention powers fast and accurate cell type annotation in single-cell RNA-seq data
Source: PLoS Comput Biol. 2025 Apr 4;21(4):e1012904. doi: 10.1371/journal.pcbi.1012904 (PMC11970913; doi:10.1371/journal.pcbi.1012904)
Supplement: S13 Table — Expression of differentially expressed genes in T cell development stages. (DOCX) [file pcbi.1012904.s030.docx]

**S13 Table: Expression of differentially expressed genes in T cell development stages.**

| Gene | stage 1 | stage 2 | stage 3 | stage 4 | stage 5 |
| --- | --- | --- | --- | --- | --- |
| *CD7* | negative | positive | positive | positive | positive |
| *CD44* | - | positive | positive | negative | negative |
| *CD2* | - | negative | positive | positive | positive |
| *CD1A* | - | negative | negative | negative | positive |
