# Supplementary material for: scTrans: Sparse attention powers fast and accurate cell type annotation in single-cell RNA-seq data
Source: PLoS Comput Biol. 2025 Apr 4;21(4):e1012904. doi: 10.1371/journal.pcbi.1012904 (PMC11970913; doi:10.1371/journal.pcbi.1012904)
Supplement: S1 Text — This file contains experimental details, parameter settings for comparison methods, ablation experiment analysis on the model structure, and simulating experiments details. (DOCX) [file pcbi.1012904.s031.docx]

# Supporting Text

## Experimental details

In the training of MCA, PBMC160k and scBloodNL datasets, scTrans performed pre-training on total datasets and fine-tuned with labeled data. During the fine-tuning, the embedding layer will freeze to prevent overfitting when the label cell count is sparse. When initializing gene embedding through PCA, in 31 tissues of MCA, all data is used to initialize gene embedding, and in two large-scale datasets sampled partial cell data is used to initialize. In cross batch annotation task, scTrans performed pre-training and fine-tuning on reference datasets. If the genes in the query dataset do not appear during the training process, they will be removed during cell type annotation or latent representation generation. In clustering analysis, we trained scTrans on reference datasets, then extract latent representation of query datasets. For example, when we extract latent representation from the MCA Pancreas dataset, we use scTrans trained on the TMS Pancreas and Baron datasets.

## Methods Comparison

Concerto: Concerto is a pre-training fine-tuning method, which use all the genes as inputs. We applied Concerto for annotation tasks on MCA, PBMC45k, mouse brain and mouse pancreas datasets. In the training of MCA datasets, we performed pre-training on total datasets and fine-tuned with labeled data. In cross batch annotation task, we performed pre-training and fine-tuning on reference datasets. All annotation tasks use all genes and parameters as follows: 1 epoch pre-train, 10 epoch fine-tune, 32 batch size, 0.1 dropout rate and 0.0001 learning rate.

scSemiGAN: scSemiGAN is a semi-supervised method that obtains latent representation through Generative Adversarial Network and uses labeled data for clustering optimization to achieve cell type annotation. All model parameters use default, except for the number of clusters, the number of clusters uses the number of cell types in labeled data. Selected 2000 highly variable genes for all tasks.

Itclust: Itclust is a transfer learning method that harnesses pre-training on labeled data to derive low-dimensional representations, subsequently applying these representations to optimize cluster and cell type annotation on the target dataset. All model parameters use default, and follow the tutorial during the training.

scDeepSort: ScDeepSort is a supervised cell type annotation method based on graph neural networks, using all genes for representation learning. On all tasks, all parameters use default. For clustering analysis task, we used the output before the classification layer as the latent representation.

TOSICA: TOSICA is a supervised transformer architecture cell type annotation tool, which performed representation learning based on gene regulatory network level. We used default model parameters and GO pathway network for training, and selected 3000 highly variable genes as inputs.

scDeepCluster: scDeepCluster is an autoencoder-based model that combines deep clustering and ZINB loss to capture low dimensional structures in scRNA seq data. We use default parameters to train scDeepCluster in clustering and mouse dendritic cell trajectory analysis tasks, the number of cell clusters estimated by the Louvain algorithm, 2000 highly variable genes were selected as inputs.

DESC: DESC is an unsupervised clustering method that obtains initialized embedding through autoencoder, balances biological and technical differences between clusters by iteratively optimizing a clustering objective function. We use default parameters to train DESC in clustering and mouse dendritic cell trajectory analysis tasks, 2000 highly variable genes were selected as inputs, other parameters use default.

scDCC: scDCC is a model based on scDeepCluster, which transforms prior knowledge into soft pairwise constraints and utilizes labeled data for semi-supervised clustering. We use default parameters to train scDCC in clustering tasks and cell trajectory analysis tasks, the number of cell clusters used the number of real cell types in the training datasets, 2000 highly variable genes were selected as inputs.

scVI: scVI is a batch correction method, which combing stochastic optimization and deep learning, and considered batch information influence. we used scVI package for batch integration of PBMC45K and human T cell development datasets. All datasets used 2000 highly variable genes as inputs, with n-layer set to 2 and n-latent set to 30, gene likelihood selected NB distribution, and other parameters use default. The output of latent representation was used for comparison.

trVAE: trVAE introduces the maximum mean discrepancy in transfer learning into the conditional variational autoencoder, thereby correcting the differences between different source datasets. We performed trVAE followed the given example, used 3000 highly variable genes as input, encoder layer is (128, 32), decoder layer is (32, 128), 200 training epochs, 512 batch size.

## Simulation experiments:

## We utilized the splatter to create simulated datasets with varying sequencing depths. During the simulation, we used the splatter parameters as follows: nGene was set to 10000, batchCells to 10000, and de.prob to 0.2, lib.loc ranged from 7 to 10 to simulate different sequencing depths(with a non-zero gene expression rate ranged from 5% to 35%), the cell types are 5, 10, and 15, with equal numbers of each type, while other parameters remained at their default settings. For each dataset with a specific sequencing depth, we first pre-trained using all samples, then performed stratified sampling based on cell type, selecting 10% of the labeled data as the training set and the remaining 90% as the testing set. This process was repeated five times for each dataset. To assess the accuracy of the method, we employed two evaluation metrics: accuracy and f1-macro.

## Research on the impact of different levels of cell type

We employed the PBMC160k dataset and conducted pre-training on the entire dataset. Subsequently, we used stratified sampling to select 10% of the cell type data for fine-tuning the model, while the remaining data was used for prediction. During the training process, we employed three distinct levels of cell types for training. To verify the accuracy of the annotation results, we separately evaluated the model's accuracy for annotations at each of these three levels.

## Ablation experiments:

To highlight the importance of each step in scTrans, we conducted ablation experiments to assess the impact of PCA embedding initialization, pre-training, embedding dropout, mean scaling, and model structure. PCA initialization can significantly improve the efficiency of cell type annotation, most datasets exhibit a notable decline in performance upon the removal of PCA initialization **(See in Table A-B).** This performance drop occurs because without PCA initialization, scTrans starts from zero embedding vectors may struggle to converge rapidly to an optimal solution. The gene embeddings derived from PCA capture the correlations between genes and provides a good gene initialization embedding, thereby markedly improving the accuracy of model annotations.

Pre-training significantly enhances the annotation accuracy at low train rate of the model by optimizing gene embeddings, making them more suitable for cell type classification tasks**(See in Table A-B)**. Gene embeddings obtained through PCA initialization may not adequately capture the gene features required for effective cell type classification. Particularly in large-scale datasets, gene embeddings are initialized based on sampled data, which may not represent the entire datasets comprehensively. The pre-training process addresses this limitation by refining the gene embeddings through contrastive learning, which aims to align the latent representation of original and augmented samples. This alignment ensures that the latent representation of the same cell type remains compact, thereby improving classification accuracy. This trend is particularly pronounced as the training rate decreases, both in MCA, PBMC160k and scBloodNL datasets. Pre-training enables scTrans to achieve robust annotation results even with a limited amount of labeled data available for fine-tuning.

Embedding dropout enhances the robustness of gene embedding, thereby improving annotation capabilities across different datasets **(See in Table C-F)**. In mouse brain and mouse pancreas, batch effects cause scTrans to focus on specific genes in reference datasets that may not accurately represent cell types in query datasets. To mitigate this issue and prevent the model from over-relying on certain genes, a dropout layer is added into the embedding layer. This intervention generates more robust gene embedding. In most mouse brain and mouse pancreas datasets, embedding layer dropout enhances the annotation performance of scTrans. Especially on MCA Pancreas datasets in single reference task, after removing embedding dropout the accuracy decreased about 16%.

Mean scaling has the potential to mitigate differences in gene expression distribution for the same cell type across different batches. Theoretically, cell type clusters in different datasets should exhibit similar gene expression distributions; however, batch effects often disrupt this uniformity. Mean scaling converts absolute gene expression into relative expressions, thereby reducing discrepancies in gene expression distributions of the same gene within identical cell type clusters from various batches. Mean scaling enhances the model's annotation accuracy in most datasets **(Table C-F)**. Such as in multi reference task, using mean scaling resulted in a significant improvement for each dataset, especially with about 34%, 37% and 25$\%$ accuracy improvements on TMS Pancreas, Baron and Romanov datasets.

By stacking the number of block layers, scTrans can achieve better performance. In all the above tasks, scTrans utilized the simplest structure: only one layer of blocks and a single attention head. To investigate the impact of more complex architectural designs, we experimented with additional block layers and attention heads in MCA, PBMC160k and scBloodNL datasets, all datasets use 10% labeled cells for fine-tuning. On MCA datasets, our ablation experiments indicate that incorporating more blocks improves the model's average evaluation results, improving accuracy by 1% and f1-macro score by about 2-3% **(Table G)**. Similarly, on PBMC160k and scBloodNL datasets, adding block layers and additional attention heads enhance scTrans's annotation capabilities **(Table H-I)**. Notably, on the nearly one million scBloodNL dataset, the presence of more block layers and attention heads resulted in a more pronounced improvement. These results highlight the potential of scTrans to enhance model performance through the simple operation of stacking blocks and additional attention heads.

**Table A. Accuracy of ablation experiments in MCA, PBMC160K and scBloodNL** **datasets at different train rate.** Different training rates indicate the use of labeled data with different proportions for training. w/o pretrain is the removal of the pretraining step. w/o initial is the removal of embedding initializing. w/o meanscaling is the removal of value scaling in preprocessing. All setting were run five times with random seeds, and the model performance was represented using the mean and standard deviation. Best result is displayed in bold.

|  | scTrans | w/o pretrain | w/o initial | w/o meanscaling |
| --- | --- | --- | --- | --- |
| MCA+10% | 89.15%±7.8% | **89.33%±6.06%** | 79.13%±10.57% | 89.09%±6.88% |
| MCA+5% | **87.22%±8.57%** | 83.40%±10.69% | 76.13%±11.69% | 87.20%±7.45% |
| MCA+1% | **81.33%±9.27%** | 67.12%±16.53% | 69.63%±12.42% | 80.43%±9.03% |
| PBMC160K+10% | **87.56%±0.09%** | 86.99%±0.15% | 77.5%±0.34% | - |
| PBMC160K+5% | **86.53%±0.67%** | 85.95%±0.26% | 75.73%±0.14% | - |
| PBMC160K+1% | **83.16%±0.66%** | 75.71%±10.57% | 66.97%±0.99% | - |
| scboodnl+10% | **83.13%±0.02%** | 82.37%±0.04% | 76.52%±0.02% | - |
| scboodnl+5% | **82.49%±0.05%** | 81.92%±0.07% | 75.75%±0.11% | - |
| scboodnl+1% | **79.92%±0.29%** | 79.65%±0.07% | 73.49%±0.21% | - |

**Table B.** **F1-macro score of ablation experiments in MCA, PBMC160K and scBloodNL datasets at different train rate.** Different training rates indicate the use of labeled data with different proportions for training. w/o pretrain is the removal of the pretraining step. w/o initial is the removal of embedding initializing. w/o meanscaling is the removal of value scaling in preprocessing. All setting were run five times with random seeds, and the model performance was represented using the mean and standard deviation. Best result is displayed in bold.

|  | scTrans | w/o pretrain | w/o initial | w/o meanscaling |
| --- | --- | --- | --- | --- |
| MCA+10% | 77.50%±12.13% | 76.73%±13.15% | 54.41%±10.49% | **77.55%±10.83%** |
| MCA+5% | 69.44%±14.44% | 58.36%±21.08% | 47.58%±11.34% | **70.22%±12.97%** |
| MCA+1% | **50.67%±12.62%** | 28.31%±15.58% | 35.37%±9.88% | 49.89%±12.26% |
| PBMC160K+10% | **73.7%±0.28%** | 72.26%±0.38% | 48.2%±0.69% | - |
| PBMC160K+5% | **70.07%±1.32%** | 68.81%±1.6% | 42.09%±0.79% | - |
| PBMC160K+1% | **55.38%±1.34%** | 40.82%±15.96% | 26.57%±1.15% | - |
| scboodnl+10% | **66.59%±0.24%** | 65.18%±0.13% | 54.49%±0.43% | - |
| scboodnl+5% | **65.5%±0.5%** | 64.58%±0.16% | 52.03%±0.42% | - |
| scboodnl+1% | **59.66%±1.41%** | 59.21%±0.36% | 45.59%±0.5% | - |

**Table C. Accuracy of ablation experiments in mouse brain and pancreas datasets on single reference task.** w/o meanscaling is the removal of value scaling in preprocessing. w/o dropout is the removal of embedding dropout layer. All setting were run five times with random seeds, and the model performance was represented using the mean and standard deviation. Best result is displayed in bold.

|  | scTrans | w/o meanscaling | w/o dropout |
| --- | --- | --- | --- |
| TMS-Pancreas | **54.51%±0.39%** | 24.49%±3.7% | 52.26%±0.66% |
| MCA-Pancreas | **36.17%±6.87%** | 31.13%±10.23% | 19.81%±5.18% |
| Baron | 50.92%±1.28% | **51.78%±0.7%** | 50.48%±0.6% |
| TMS-Brain | 83.4%±0.99% | 79.42%±4.63% | **83.69%±0.36%** |
| MCA-Brain | 47.84%±8.55% | 45.43%±6.46% | **50.95%±14.82%** |
| Romanov | **81.6%±1.67%** | 67.11%±6.24% | 78.88%±2.1% |

**Table D. F1-macro score of ablation experiments in mouse brain and pancreas datasets on single reference task.** w/o meanscaling is the removal of value scaling in preprocessing. w/o dropout is the removal of embedding dropout layer. All setting were run five times with random seeds, and the model performance was represented using the mean and standard deviation. Best result is displayed in bold.

|  | scTrans | w/o meanscaling | w/o dropout |
| --- | --- | --- | --- |
| TMS-Pancreas | **24.11%±0.32%** | 10.27%±2.03% | 23.76%±0.37% |
| MCA-Pancreas | **23.68%±1.73%** | 19.39%±2.22% | 23.09%±3% |
| Baron | 25.55%±0.64% | **25.6%±1.06%** | 25.38%±0.99% |
| TMS-Brain | 30.91%±0.46% | 29.17%±3.17% | **31.07%±0.22%** |
| MCA-Brain | **23.81%±1.58%** | 19.99%±1.47% | 20.52%±1.42% |
| Romanov | **25.29%±0.35%** | 20.07%±1.95% | 24.67%±0.6% |

**Table E. Accuracy of ablation experiments in mouse brain and pancreas datasets on multi reference task.** w/o meanscaling is the removal of value scaling in preprocessing. w/o dropout is the removal of embedding dropout layer. All setting were run five times with random seeds, and the model performance was represented using the mean and standard deviation. Best result is displayed in bold.

|  | scTrans | w/o meanscaling | w/o dropout |
| --- | --- | --- | --- |
| TMS-Pancreas | **87.07%±0.72%** | 53.87%±0.43% | 76.48%±7.48% |
| MCA-Pancreas | **41.62%±2.08%** | 39.5%±1.18% | 39.16%±1.29% |
| Baron | 82.59%±7.02% | 45.87%±7.85% | **86.76%±1.68%** |
| TMS-Brain | **93.7%±0.09%** | 93.24%±0.42% | 93.24%±0.84% |
| MCA-Brain | 90.85%±0.75% | 87.29%±1.71% | **91.16%±0.49%** |
| Romanov | **70.47%±1.08%** | 45.78%±5.48% | 68.07%±0.4% |

**Table F. F1-macro score of ablation experiments in mouse brain and pancreas datasets on multi reference task.** w/o meanscaling is the removal of value scaling in preprocessing. w/o dropout is the removal of embedding dropout layer. All setting were run five times with random seeds, and the model performance was represented using the mean and standard deviation. Best result is displayed in bold.

|  | scTrans | w/o meanscaling | w/o dropout |
| --- | --- | --- | --- |
| TMS-Pancreas | **35.21%±3.04%** | 23.93%±0.85% | 34.61%±4.26% |
| MCA-Pancreas | **20.64%±1.3%** | 17.42%±1.54% | 17.04%±0.91% |
| Baron | 35.45%±2.47% | 14.09%±3.15% | **37.1%±2.58%** |
| TMS-Brain | **41.36%±0.95%** | 38.96%±1.14% | 39.9%±0.59% |
| MCA-Brain | 27.39%±2.94% | 27.57%±1.42% | **29.81%±1.1%** |
| Romanov | **26.12%±1.05%** | 18.44%±2.4% | 24.83%±2.23% |

**Table G. Accuracy and f1-macro of ablation experiments in MCA datasets at different block layer and attention head.** The encoder of scTrans can stack multiple layers of attention blocks and use multi head attention. 31 tissues datasets from the MCA datasets were used, with each tissues using 10% labeled data and the remaining 90% used for prediction. All setting were run five times with random seeds, and the model performance was represented using the mean and standard deviation. Best result is displayed in bold.

|  | 1layer | | 2layer | |
| --- | --- | --- | --- | --- |
|  | accuracy | f1-macro | accuracy | f1-macro |
| 1head | 89.15%±6.99% | 77.7%±10.2% | **90.07%±5.96%** | **80.18%±9.54%** |
| 2head | 89.45%±6.46% | 76.34%±10.6% | 89.84%±6.12% | 78.55%±10.42% |
| 4head | 89.3%±6.29% | 73.91%±11.64% | 88.92%±6.54% | 73.5%±11.74% |

**Table H. Accuracy and f1-macro of ablation experiments in PBMC160K datasets at different block layer and attention head.** The encoder of scTrans can stack multiple layers of attention blocks and use multi head attention. with 10% labeled data in PBMC160K were used and the remaining 90% used for prediction. All setting were run five times with random seeds, and the model performance was represented using the mean and standard deviation. Best result is displayed in bold.

|  | 1layer | | 2layer | |
| --- | --- | --- | --- | --- |
|  | accuracy | f1-macro | accuracy | f1-macro |
| 1head | 87.56%±0.09% | 73.7%±0.28% | 88.14%±0.15% | 75.17%±0.27% |
| 2head | 88.25%±0.07% | 75.09%±0.29% | **89.44%±0.14%** | **77.18%±0.44%** |
| 4head | 89.24%±0.45% | 76.29%±0.72% | 89.42%±0.1% | 76.1%±0.51% |

**Table I. Accuracy and f1-macro of ablation experiments in scBloodNL datasets at different block layer and attention head.** The encoder of scTrans can stack multiple layers of attention blocks and use multi head attention. with 10% labeled data in scBloodNL were used and the remaining 90% used for prediction. All setting were run five times with random seeds, and the model performance was represented using the mean and standard deviation. Best result is displayed in bold.

|  | 1layer | | 2layer | |
| --- | --- | --- | --- | --- |
|  | accuracy | f1-macro | accuracy | f1-macro |
| 1head | 83.13%±0.02% | 66.59%±0.24% | 84.65%±0.27% | 69.25%±0.59% |
| 2head | 84.23%±0.12% | 68.34%±0.13% | 85.51%±0.1% | 70.83%±0.24% |
| 4head | 84.72%±0.09% | 69.06%±0.24% | **85.61%±0.12%** | **71.05%±0.43%** |
